# Glutathione peroxidase‐1 overexpression reduces oxidative stress, and improves pathology and proteome remodeling in the kidneys of old mice

**DOI:** 10.1111/acel.13154

**Published:** 2020-05-13

**Authors:** Yi Chu, Renny S. Lan, Rui Huang, Hao Feng, Rahul Kumar, Sanjana Dayal, Kung‐Sik Chan, Dao‐Fu Dai

**Affiliations:** ^1^ Department of Pathology Carver College of Medicine University of Iowa Iowa City Iowa; ^2^ Proteomics Core Department of Biochemistry and Molecular Biology University of Arkansas Medical Sciences College of Medicine Little Rock Arkansas; ^3^ Department of Statistics and Actuarial Science College of Liberal Arts and Sciences University of Iowa Iowa City Iowa; ^4^ Department of Internal Medicine Carver College of Medicine University of Iowa Iowa City Iowa

**Keywords:** glutathione peroxidase‐1, kidney aging, mitochondria, proteomics, reactive oxygen species

## Abstract

This study investigated the direct roles of hydrogen peroxide (H_2_O_2_) in kidney aging using transgenic mice overexpressing glutathione peroxidase‐1 (GPX1 TG). We demonstrated that kidneys in old mice recapitulated kidneys in elderly humans and were characterized by glomerulosclerosis, tubular atrophy, interstitial fibrosis, and loss of cortical mass. Scavenging H_2_O_2_ by GPX1 TG significantly reduced mitochondrial and total cellular reactive oxygen species (ROS) and mitigated oxidative damage, thus improving these pathologies. The potential mechanisms by which ROS are increased in the aged kidney include a decreased abundance of an anti‐aging hormone, Klotho, in kidney tissue, and decreased expression of nuclear respiratory factor 2 (Nrf2), a master regulator of the stress response. Decreased Klotho or Nrf2 was not improved in the kidneys of old GPX1 TG mice, even though mitochondrial morphology was better preserved. Using laser capture microdissection followed by label‐free shotgun proteomics analysis, we show that the glomerular proteome in old mice was characterized by decreased abundance of cytoskeletal proteins (critical for maintaining normal glomerular function) and heat shock proteins, leading to increased accumulation of apolipoprotein E and inflammatory molecules. Targeted proteomic analysis of kidney tubules from old mice showed decreased abundance of fatty acid oxidation enzymes and antioxidant proteins, as well as increased abundance of glycolytic enzymes and molecular chaperones. GPX1 TG partially attenuated the remodeling of glomerular and tubule proteomes in aged kidneys. In summary, mitochondria from GPX1 TG mice are protected and kidney aging is ameliorated via its antioxidant activities, independent and downstream of Nrf2 or Klotho signaling.

## Graphical abstract

Glutathione Peroxidase‐1 overexpression reduces oxidative stress, preserves mitochondrial cristae structures, and improves kidney pathology and proteome gemodeling in the glomeruli and tubules of old mouse kidneys. These beneficial effects are downstream and independent of Klotho and Nrf2 signaling.

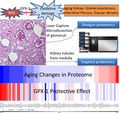

## 1 INTRODUCTION

Kidney aging manifests as a progressive decline in kidney function with age, which increases the risk of chronic kidney diseases (CKD) and end‐stage renal diseases (ESRD). It is characterized by slow, progressive glomerulosclerosis, tubular atrophy, interstitial fibrosis, and arteriosclerosis (Bitzer & Wiggins, 2016). Epidemiological studies show that CKD are present in >30% of the elderly population in the US, and old age per se has the highest correlation with a low glomerular filtration rate (“2018 USRDS annual data report: Volume one: CKD in the General Population,” 2018). CKD increase the risk of all‐cause and cardiovascular deaths by ~2‐fold (Tonelli et al., 2006). In the elderly that do not meet the criteria of CKD, old age increases susceptibility to acute kidney injury and decreases its reparative capacity (Chen et al., 2007; Wang, Bonventre, & Parrish, 2014). These data emphasize the importance of understanding the molecular mechanisms of kidney aging in order to develop therapies to prevent or delay this condition (Bitzer & Wiggins, 2016).

Reactive oxygen species (ROS) signaling pathways are central to several paradigms that modulate aging and healthspan. The mitochondrial free radical theory of aging proposes that mitochondria are both primary sources of ROS and primary targets of ROS‐induced damage (Harman, 1972). Notably, mitochondrial damage is a hallmark of several aging phenotypes, and primary mitochondrial damage due to the accumulation of mitochondrial DNA deletion “accelerates” aging (Vermulst et al., 2008). Protection of mitochondria by mitochondrial‐targeted antioxidants extends lifespans and attenuates changes due to aging in several organ systems (Schriner et al., 2005). These findings suggest a causal link between mitochondrial damage induced by ROS and the aging process. The critical roles of mitochondrial oxidative stress have been demonstrated in murine aging (Schriner et al., 2005) and healthspan, including cardiac (Dai et al., 2009) and skeletal muscle aging (Lee et al., 2010). However, the direct role of ROS has not been investigated in kidney aging.

Glutathione peroxidase‐1 (GPX1) is an intracellular antioxidant enzyme which uses reduced glutathione (GSH) to convert H_2_O_2_ to water to limit its harmful effects. GPX1 is present within mitochondria as well as in the cytosol (Li, Yan, Yang, Oberley, & Oberley, 2000). GPX1 modulates redox‐dependent mitochondrial function (Handy et al., 2009). Overexpression of GPX1 attenuates myocardial ischemia–reperfusion injury (Yoshida et al., 1996) and doxorubicin‐induced cell death (Gouaze et al., 2001), both of which involves mitochondria. In the present study, we tested the hypothesis that ROS and genetic overexpression of GPX1 may play critical roles in kidney aging. We demonstrate that scavenging ROS and preserving mitochondrial function by overexpression of GPX1 improved kidney pathology associated with aging and prevented the remodeling of glomerular and tubular proteomes in old mice.

## 2 RESULTS

### 2.1 Overexpression of GPX1 in transgenic mice

**FIGURE 1:**
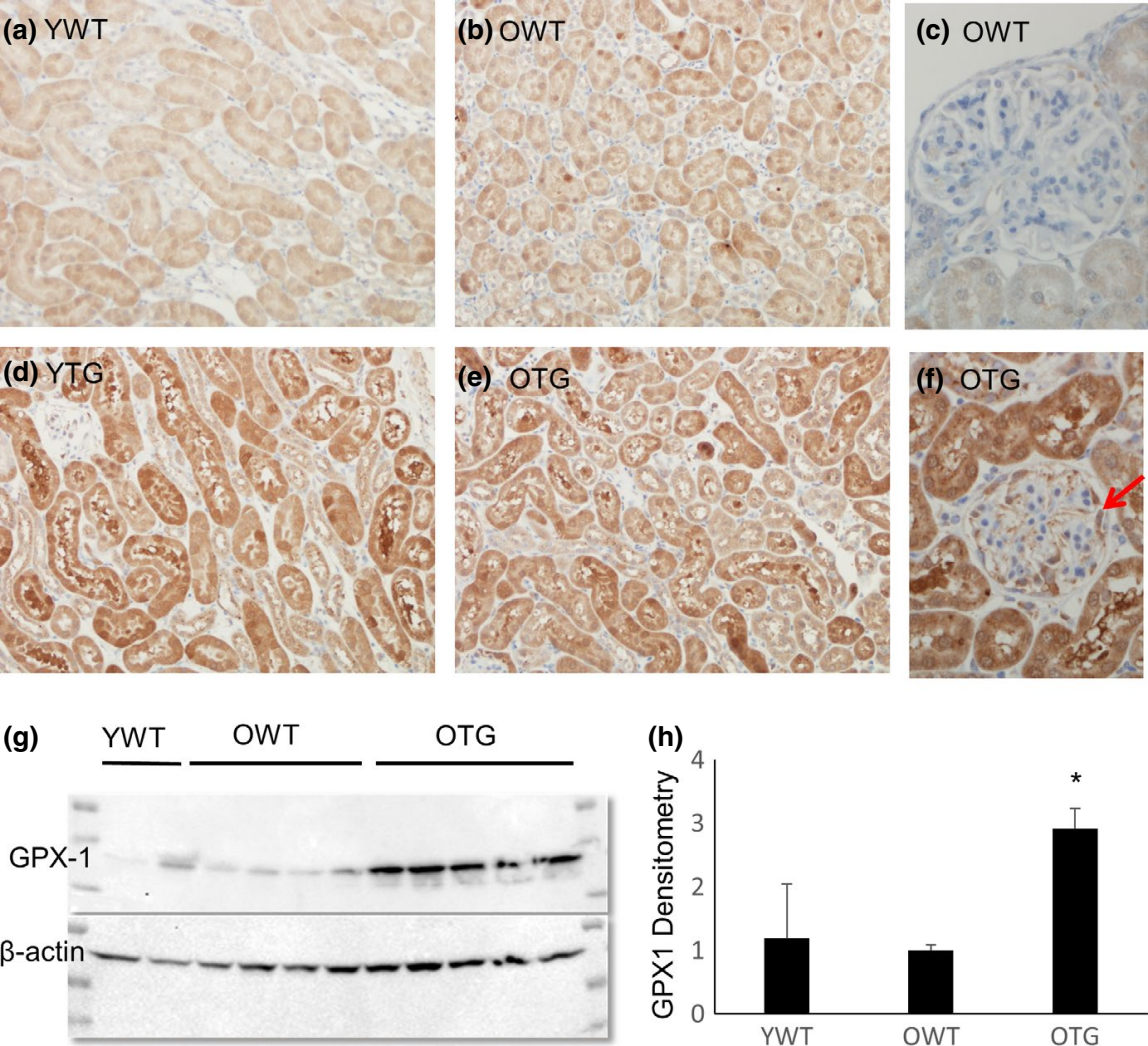
Levels of GPX1 protein in kidneys of wild‐type (WT) and GPX1 transgenic (TG) mice, at young (Y, 4–5 months old) and old (O, 21–23 months old) ages. (a–f) Immunohistochemistry for GPX1. The GPX1 TG was overexpressed in tubular epithelial cells (d, e) and podocytes (f, arrow). (g, h) Immunoblots showing an ~3‐fold increase of GPX1 in kidneys from old TG mice compared with old WT mice. *N* = 4–5. *p < .05

Overexpression of the GPX1 transgene (TG) was confirmed in the kidney. Aging had no effect on GPX1 expression in wild‐type (WT) mice (Figure 1a,b). In TG mice, GPX1 was overexpressed in the cortical tubular epithelial cells (Figure 1d,e), and in the glomerular podocytes (Figure 1f). Immunoblots of kidney lysates showed ~3‐fold overexpression of GPX1 in old TG mice, compared to old WT mice (Figure 1g,h). Immunofluorescence showed that GPX1 staining highly overlapped with the staining of VDAC1, a mitochondrial protein (Figure S1), indicating that GPX1 is highly enriched in the mitochondria. Additionally, scattered, non‐overlapping staining for GPX1 indicated that GPX1 was also present outside mitochondria.

### 2.2 GPX1 overexpression attenuates kidney pathologies in old mice

**FIGURE 2:**
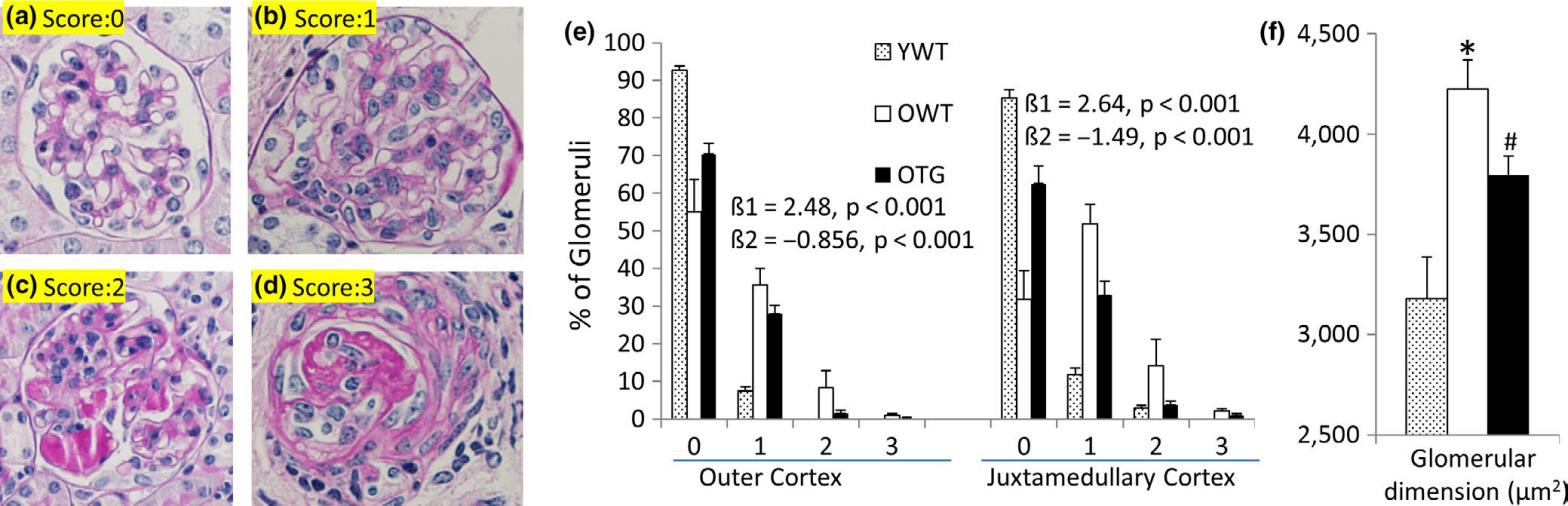
Glomerular injury scores (GS) of young WT (YWT), old WT (OWT) and old TG (OTG) mice. (a–d) Representative images of glomeruli with GS of 0 (normal), 1 (mild mesangial sclerosis), 2 (moderate glomerulosclerosis), and 3 (severe glomerulosclerosis). (e) Quantification of GS in the outer cortex and juxtamedullary cortex of the three groups of mice. Proportional odds linear regression: the log odds of GS that is greater than score *k* is = *β*_1_ × YO *β*_2_ × TG − *ζ_k_*; *β*_1_ is the difference in the log odds of GS between young and old mice (age effects); *β*_2_ is the difference in the log odds of GS between old WT and old TG (effect of GPX1 overexpression) (See text and supplement for details). (f) Quantification of glomerular areas. *N* = 4–6; *p < .05 versus YWT, #*p* < .05 versus OWT

Age‐dependent glomerulosclerosis was evaluated using a glomerular injury score (GS) ranging from normal (score 0) to severe (75%–100%) glomerulosclerosis (score 3, see Section 4 for details) (Figure 2). Proportional odds linear regression modeling was used to analyze the effects of aging (old versus young WT) and GPX1 overexpression (old TG versus old WT) on GS (Figure 2). It is well known that glomerulosclerosis is generally more severe in the juxtamedullary region than the outer cortex (Sweetwyne et al., 2017), which we confirmed in this study. The fitted regression for the outer cortex showed a coefficient of *β*1 = 2.48 and *β*2 = −0.856 (both *p* < .01) for effects of aging and the GPX1 transgene on the severity of glomerular injury, respectively. In addition, the fitted regression for the juxtamedullary cortex showed a coefficient of *β*1 = 2.64 and *β*2 = −1.49, (both *p* < .01). A larger *β*1 (“slope”) in the juxtamedullary cortex relative to the outer cortex indicated a more prominent change in this region due to aging, consistent with a previous report (Sweetwyne et al., 2017). GPX1 overexpression significantly attenuated glomerular injury, providing ~35% and ~56% protection from changes due to aging in the outer and juxtamedullary cortices, respectively (Figure 2e). The average glomerular size increased by ~25% with aging, from 3,179 ± 208 μm^2^ in young wild‐type (YWT) to 4,224 ± 145 μm^2^ in old wild‐type (OWT; *p* < .01), suggesting a compensatory glomerular hypertrophy in response to the loss of glomerular mass. GPX1 overexpression partially attenuated this glomerular hypertrophy (*p* < .05 OTG versus OWT, Figure 2f).

**FIGURE 3:**
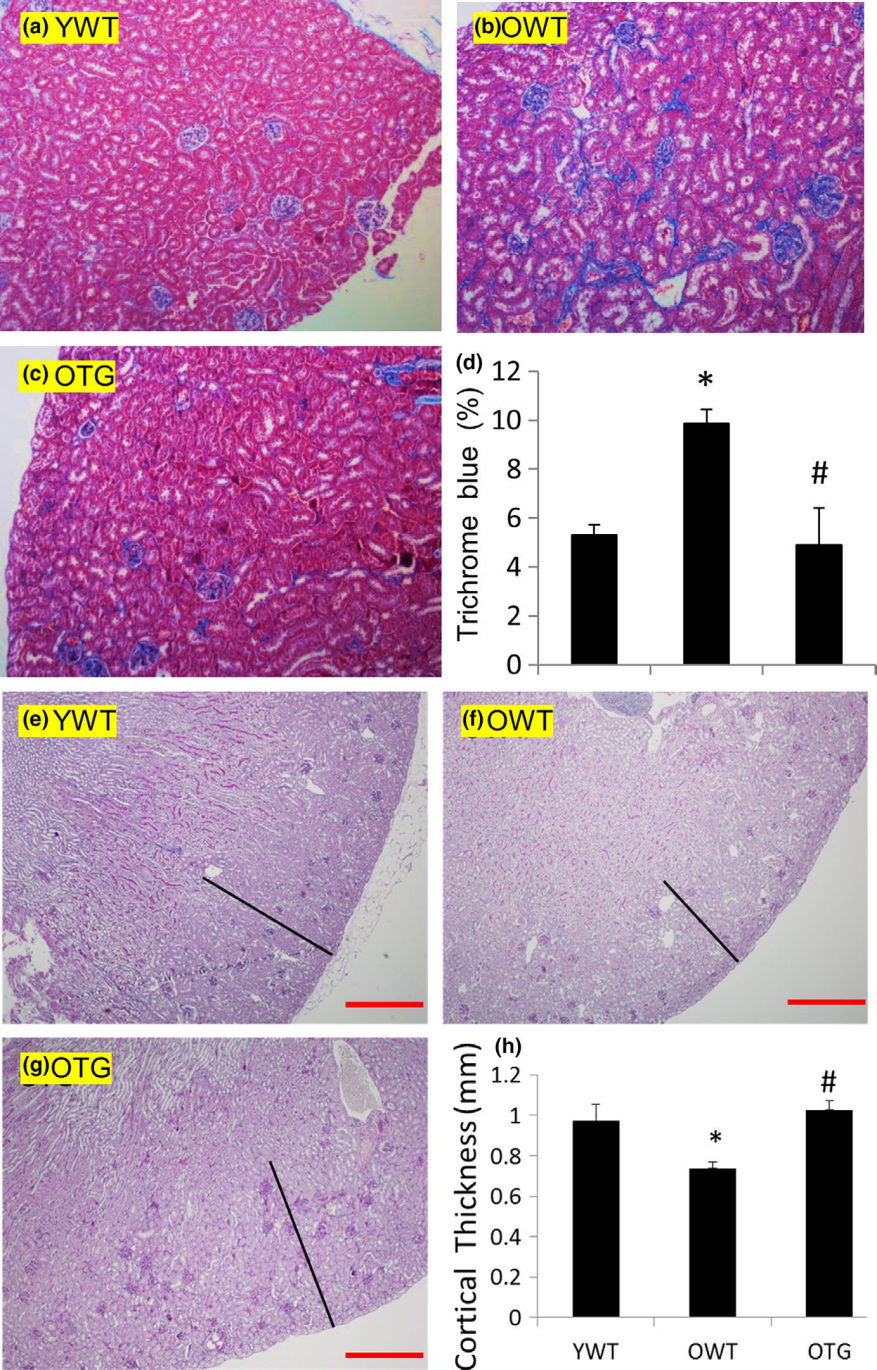
(a–c) Representative images of Masson trichrome staining of the kidney cortex: from YWT, OWT, and OTG mice. Blue indicates fibrosis. (d) Quantification of blue areas. *N* = 4–6. *p < .05 versus YWT, #*p* < .05 versus OWT. (e–g) Representative of the kidney cortex (indicated by black lines) in YWT, OWT, and OTG mice. (h) Quantification of cortical thickness. *N* = 4–6. *p < .05 versus YWT, #*p* < .05 versus OWT. Red scale bar: 0.5 mm

Increased glomerulosclerosis was accompanied by tubular atrophy and interstitial fibrosis in old mice, with cortical fibrosis observed in ~10% of the area in OWT versus ~5% in YWT (Figure 3a,b). In contrast, GPX1 overexpression significantly reduced interstitial fibrosis (Figure 3c,d). Kidney cortical atrophy is well documented in human aging (Glodny et al., 2009), likely due to glomerulosclerosis (Neugarten, Gallo, Silbiger, & Kasiske, 1999), tubular atrophy and interstitial fibrosis. Consistent with this, there was a significant thinning of the kidney cortex in OWT, with an ~25% decrease in cortical thickness (Figure 3h). However, overexpression of GPX1 significantly preserved cortical thickness in old mice (Figure 3h).

### 2.3 GPX1 overexpression, independent of Nrf2, attenuates oxidative damage and preserves mitochondria

**FIGURE 4:**
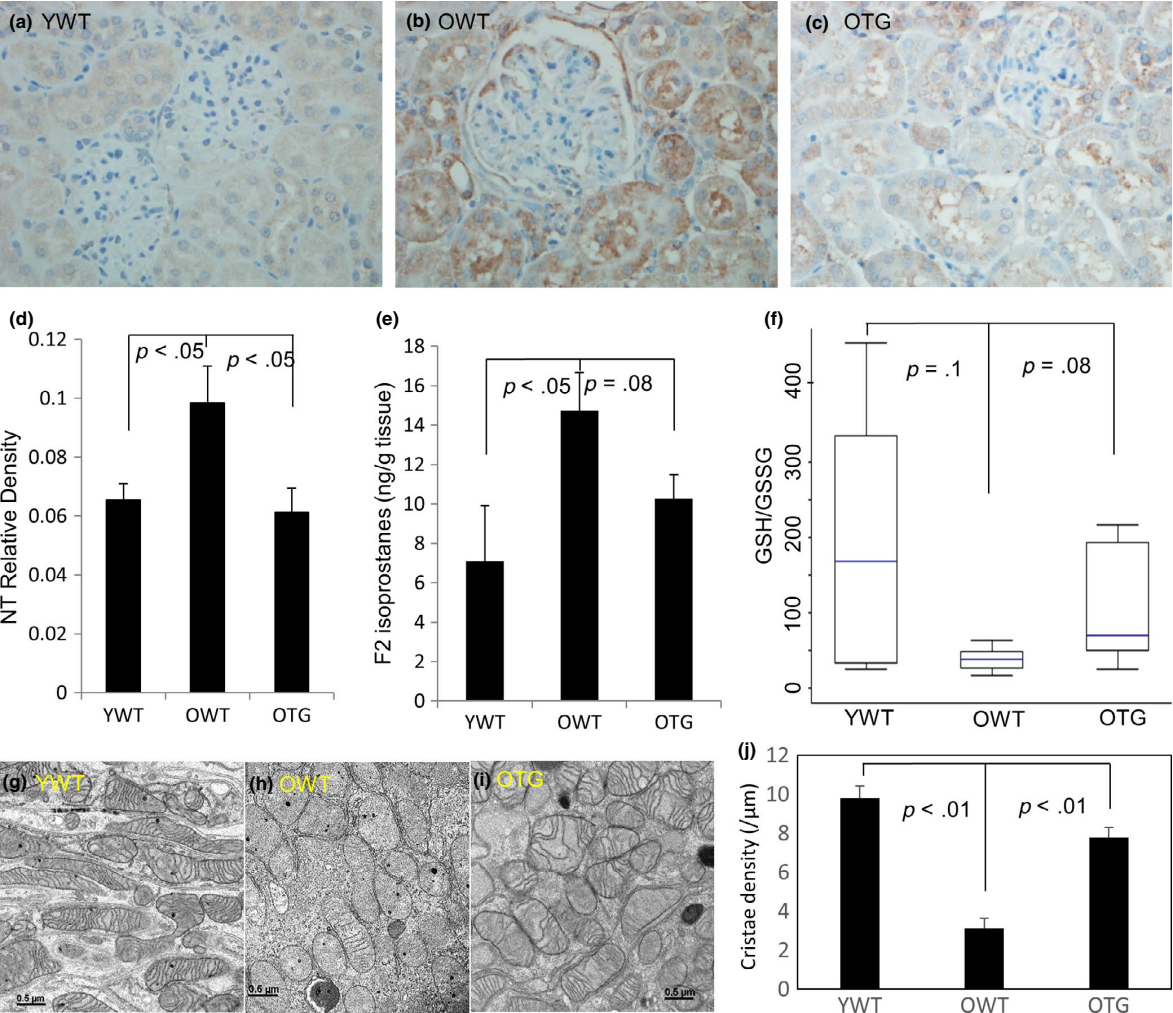
(a–c) Immunohistochemistry for nitrotyrosine (brown color) in YWT, OWT, and OTG, with quantification of staining density (d). (e) Levels of F2 isoprostanes, a marker of lipid oxidative damage, in kidney lysates (ng/g tissue). (f) Ratios of reduced glutathione to oxidized glutathione (GSH/GSSG) in kidney lysates. Nitrotyrosine and F2 isoprostanes were significantly increased in old mice, and GPX1 overexpression significantly decreased (nitrotyrosine) or tended to reduce (F2 isoprostanes) the increases observed in old mice. In contrast, the GSH/GSSG ratio tended to decrease in old mice, and GPX1 overexpression tended to rescue the decrease. *N* = 4–6. (g–i) Representative electron micrographs of mitochondria isolated from kidney cortex of YWT, OWT, and OTG. Scale bar: 0.5 µm. (j). Quantification of mitochondrial cristae density (number of cristae/µm). *N* = 3

Immunohistochemistry was performed to examine nitrotyrosine, a marker of protein oxidative damage. A significant increase in nitrotyrosine was observed in tubular epithelial cells and podocytes of old mice (Figure 4b,d), which was significantly decreased upon GPX1 overexpression (Figure 4c,d). F2 isoprostane is an oxidative product formed by ROS‐induced peroxidation of arachidonic acid and was significantly increased in old mice compared with young mice (*p* < .05). Overexpressing GPX1 tended to decrease F2 isoprostane in old mice (Figure 4e). Furthermore, old mice had a decreased GSH/GSSG ratio (*p* = .1), indicative of a more oxidized “redox status,” and this was largely attenuated upon GPX1 overexpression (*p* = .08; Figure 4f).

We used electron microscopy to examine the mitochondrial ultrastructure in the kidney cortex. Kidneys from old mice had far fewer mitochondrial cristae than young kidneys, suggesting mitochondrial oxidative phosphorylation was less efficient in the kidneys of old mice. Impressively, GPX1 overexpression preserved mitochondrial cristae in kidney cortex of old mice (Figure 4g–j).

**FIGURE 5:**
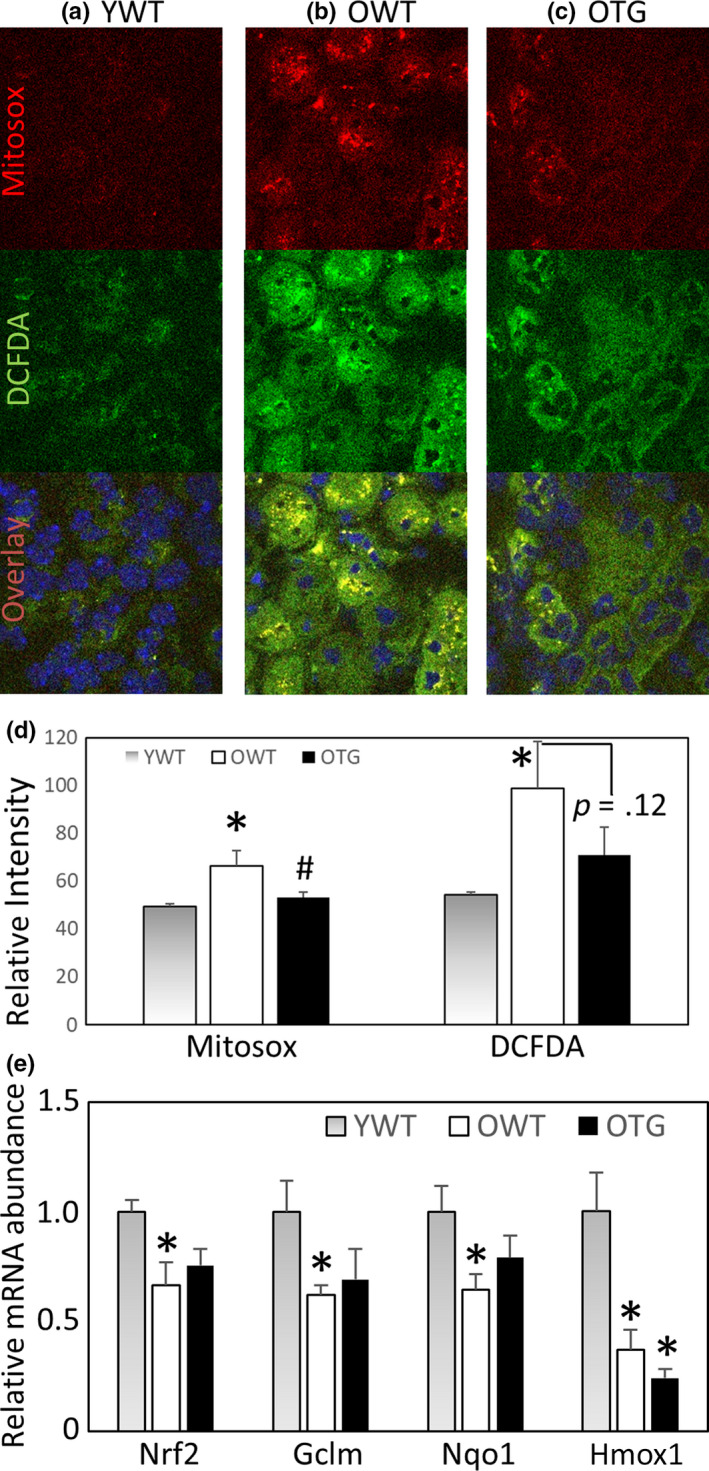
(a–c) Representative images of ex vivo staining with MitoSOX (top row), an indicator of mitochondrial superoxide, DCFDA (middle row), an indicator of total cellular ROS_,_ and the overlay images (bottom row) with Hoechst 33342 nuclear stain. (D) Quantification of relative fluorescence intensity for MitoSOX and DCFDA. *N* = 3. *p < .05 versus YWT, #*p* < .05 versus OWT. (e) Relative mRNA abundance of Nrf2 and its targets Gclm, Nqo1 and Hmox1 in kidney. *N* = 3–5. *p < .05 versus YWT

Decreases in mitochondrial cristae are expected to impair electron transport and increase ROS production in kidneys of old mice (Cogliati et al., 2013). We performed ex vivo staining with MitoSOX and DCFDA to detect mitochondrial superoxide and total cellular ROS levels, respectively, in fresh kidney tissues (Figure 5a–d). Staining of both MitoSOX and DCFDA was increased in old mice (Figure 5b) versus young mice (Figure 5a), and GPX1 TG significantly decreased mitochondrial superoxide and tended to decrease total ROS levels (Figure 5c,d).

To examine the potential mechanisms that lead to increased ROS and oxidative damage in aging, we analyzed the expression of a master regulator of oxidative stress, Nrf2, by quantitative RT‐PCR. We found a significant decrease in Nrf2 expression and its downstream target genes in the kidneys of old mice. Overexpressing GPX1, a downstream target of Nrf2, did not have any effect on Nrf2 expression (Figure 5e).

### 2.4 Glomerular proteomics and effects of GPX1 overexpression in old mice

**FIGURE 6:**
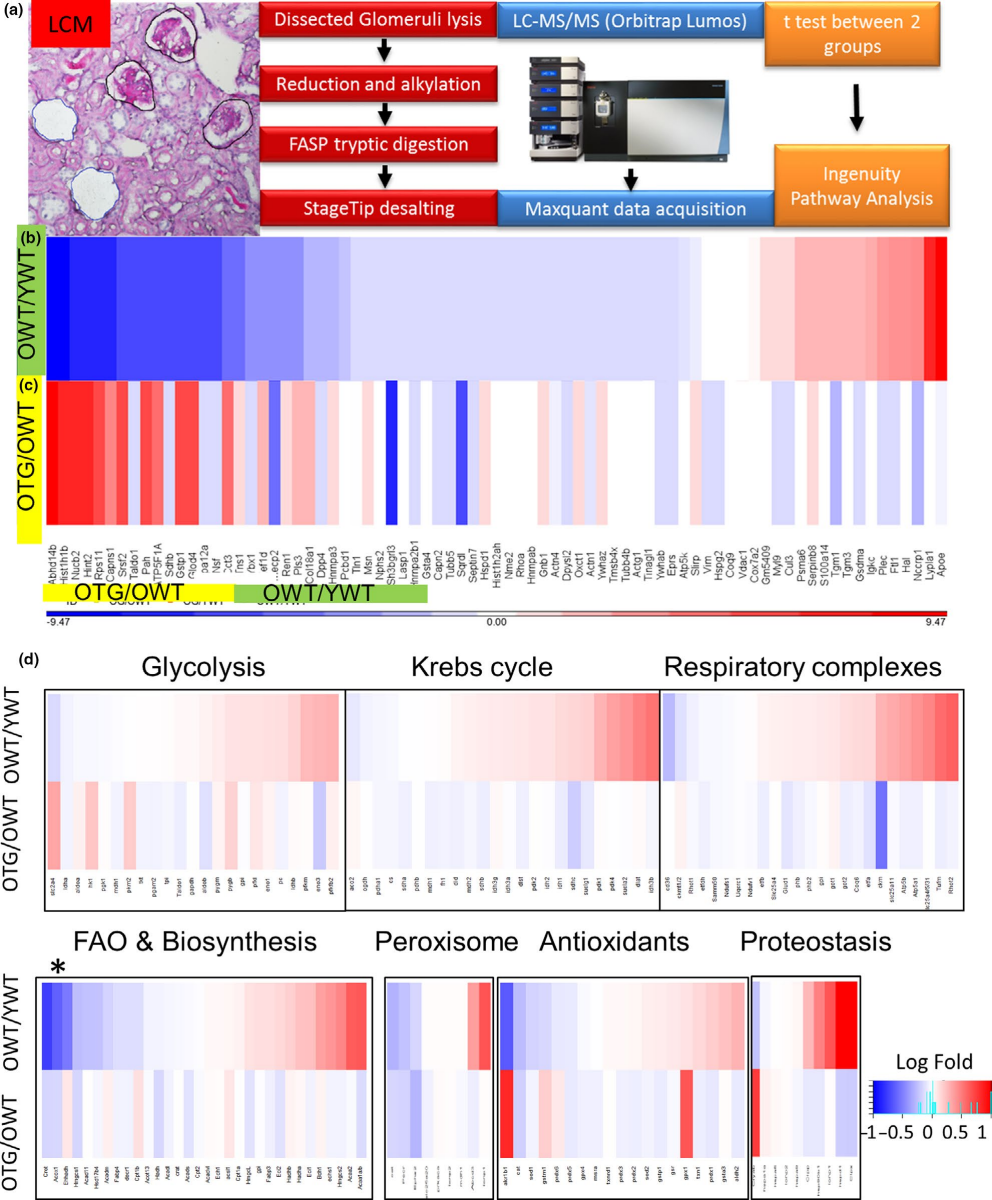
Label‐free shotgun proteomics of glomeruli from YWT, OWT, and OTG mice (*n* = 4 per group) (a–c). (a) Glomeruli were collected using laser capture microdissection, processed for proteomics (Orbitrap Lumos), and analyzed using Student's *t* tests (with corrections) for OWT versus YWT (aging effect) and OTG versus OWT (transgenic effect). (b, c) Heatmaps show comparisons of protein abundance of OWT versus YWT and OTG versus OWT, respectively. Red indicates increases, and blue indicates decreases, of abundance. Proteins were ranked according to fold‐change with age for 77 proteins (see Table S1 for details), *N* = 4. (d) Targeted proteomics of tubules from YWT, OWT and OTG, which was focused on 139 selected proteins involved in glycolysis, Krebs cycle, mitochondrial respiratory complexes, fatty acid oxidation & biosynthesis, peroxisomes, antioxidants, and proteostasis (see Table S2 for details). In each heatmap, top row indicates OWT versus YWT (aging effect) and bottom row indicates OTG versus OWT (effect of GPX1 overexpression). *Substantial decrease in key enzymes of fatty acid oxidation with aging

To gain a deeper understanding of the changes that occur in the kidney due to aging, we performed separate proteomic analysis on glomeruli and tubules. Glomeruli, which function in the ultrafiltration of plasma to produce urine, are the primary site affected in several kidney diseases that often progress to CKD. We collected glomeruli by laser capture microdissection and performed label‐free shotgun proteomics (Figure 6a). Six hundred and sixteen proteins were identified in all glomerular samples, of which 48 proteins significantly changed with age (*p* < .05, Table S1). The relative abundance of 77 proteins with differential expression between OWT versus YWT (*p* < .1) are shown in heat maps (Figure 6b). Approximately 3/4 of these proteins decreased with aging (lower abundance in OWT, blue on the heat map), whereas the remaining ~1/4 increased with aging (higher abundance in OWT, red on the heat map). Of the 56 proteins that were decreased with aging, 24 were partially attenuated by GPX1 overexpression (red, or increase in OTG versus OWT, Figure 6c). Conversely, of the 16 proteins that were increased with aging, 8 were decreased in OTG versus OWT (blue in Figure 6c). Thus, GPX1 TG reversed the aging changes in ~50% of the proteins in the glomeruli, as shown by the reversal of color in OTG/OWT versus OWT/YWT (Figure 6b,c).

To confirm the above proteomic findings, we selected two proteins to analyze by immunoblotting: ATP synthase (ATP5A), a mitochondrial complex V protein involved in ATP generation during the final step of oxidative phosphorylation, and Hsp70, a heat shock protein which assists in protein folding and proteostasis. Immunoblots confirmed that ATP5A was significantly decreased in old mice and that overexpression of GPX1 attenuated this decrease (Figure S2a). Similar results were obtained with HSP70 (Figure S2b). Thus, changes detected through our proteomics analysis were validated by immunoblotting, consistent with our previous study (Dai et al., 2012).

### 2.5 Targeted proteomics of tubules in old mice and the effects of GPX1 TG

To investigate changes in proteins involved in essential pathways in kidney tubules, including glycolysis, fatty acid oxidation, Krebs cycle, antioxidant, proteostasis, and peroxisomal pathways, we performed targeted proteomics of kidney tubules from young and old mice (Figure 6d, detailed in Table S2). Six out of 23 glycolytic proteins, including the key enzyme phosphofructokinase, were significantly increased in old mice. In contrast, 5 out of 9 enzymes responsible for fatty acid oxidation, including those involved in key initial steps of oxidation (i.e., acyl‐CoA dehydrogenase within mitochondria and acyl‐CoA oxidase within peroxisomes), were significantly decreased in old mice. Other enzymes involved in the intermediate steps of oxidation were upregulated in old mice (Table S2).

Among Krebs cycle proteins, isocitrate dehydrogenase and succinate dehydrogenase complex were significantly increased with aging, whereas aconitase was slightly decreased in old mice. Old mice showed increases in pyruvate dehydrogenase kinase, which functions to inhibit the activity of the pyruvate dehydrogenase complex that oxidizes pyruvate to acetyl‐CoA in the Krebs cycle. Respiratory complexes I, III and IV were not changed, however complex II (succinate dehydrogenase complex, which also belongs to Krebs cycle) and complex V (ATP synthase) significantly increased with age. The antioxidants aldo‐keto reductase and catalase were significantly decreased in old mice, which might contribute to increased oxidative damage to lipids and proteins. Further, there were increased heat shock proteins and proteases/peptidases in old mice (Table S2).

Like OWT, OTG kidney tubules had an increase in enzymes that are involved in glycolysis and a decrease in enzymes that are involved in fatty acid oxidation. OTG had significantly higher aldo‐keto reductase, GPX1, glutathione S‐transferase, but slightly lower peroxiredoxin.

### 2.6 ROS and Klotho in kidney aging

Klotho is an anti‐aging hormone involved in senescence, endogenous antioxidants, apoptosis and phosphate homeostasis (Kuro‐o et al., 1997; Kurosu et al., 2005). Kidney tubular epithelial cells are the main source of circulating Klotho (Olauson, Mencke, Hillebrands, & Larsson, 2017). We found that full‐length membranous Klotho (designated tissue Klotho) was significantly decreased in OWT kidney tissue by ~45%, and GPX1 TG did not prevent this decrease (Figure S3). The decrease of Klotho expression in kidney tubular epithelial cells may represent another potential mechanism of kidney aging. As such, kidneys of 8‐week‐old Klotho‐deficient mice (Klotho^kl/kl^) with accelerated aging phenotypes (Xie et al., 2012) had a significant decrease in GPX1 (Figure S4). This was in parallel with an ~2.5‐fold increase in nitrotyrosine, and a >3‐fold increase in interstitial fibrosis and glomerulosclerosis in the kidney cortex (Figure S5). Collectively, these findings suggest that Klotho deficiency (in the context of aging or Klotho‐deficient mice) may augment protein oxidative damage by decreasing proteins in the antioxidant defense pathways, such as GPX1.

## 3 DISCUSSION

There are 5 major findings presented in this study. First, the aging phenotype in mouse kidneys recapitulates the pathology observed in the kidneys of elderly people. Second, scavenging H_2_O_2_ by GPX1 overexpression significantly ameliorate kidney aging phenotypes, concomitant with reduced oxidative damage and preserved mitochondria. These findings provide evidence that ROS play a direct role in pathologic phenotypes in the kidney that arise with aging and indicate the potential benefits of overexpressing GPX1 to circumvent these effects. Third, the glomerular proteome of old mice is characterized by decreased expression of several cytoskeletal proteins that are critical for maintaining normal glomerular filtration. These novel findings suggest that proteome remodeling that occurs in aging is similar to protein changes found in several glomerulopathies. Fourth, analysis of the tubular proteome in old mice suggests that there is decreased abundance of proteins involved in fatty acid oxidation and an increase in those involved in the glycolytic pathway. Fifth, the partial protection afforded by overexpressing GPX1 is downstream and independent of Nrf2 and Klotho.

Imaging studies of aged human kidneys demonstrate decreased cortical thickness and increased medullary volume and atherosclerosis (Lorenz et al., 2010). Histopathological analysis shows increased global glomerulosclerosis, tubular atrophy, interstitial fibrosis, arteriosclerosis, and glomerular hypertrophy (Rule et al., 2010). Our data from aged mice show increased glomerulosclerosis and compensatory glomerular hypertrophy, tubular atrophy, interstitial fibrosis, and reduced cortical thickness, resembling findings from aged human kidneys. However, in contrast to significant arteriosclerosis observed in humans, we did not find evidence of arteriosclerosis in aged mice, consistent with a prior study (Sweetwyne et al., 2017). This phenotype coincides with reduced severity of other age‐related comorbidities in mice, such as the absence of diabetes and hypertension (Dai et al., 2009).

In this study, overexpression of GPX1 significantly ameliorated many pathological features observed in kidneys from aged mice (Figures 2, 3, 4) and was accompanied by protection from oxidative damage, indicated by nitrotyrosine (protein nitrosylation) and F2 isoprostane (lipid peroxidation) (Figure 5). Further, the cellular redox status changed to a more oxidized state in aged kidneys, as shown by a decreased GSH/GSSG ratio due to depletion of GSH reducing equivalent and accumulation of oxidized GSSG (Figure 5f). Overexpression of GPX1 partially improved this redox status. Taken together, these findings emphasize the roles of ROS, particularly mitochondrial H_2_O_2,_ in kidney pathology due to aging. Our findings are also consistent with a recent study using SS31 to reverse kidney aging (Sweetwyne et al., 2017). SS31 is a tetra‐peptide that binds to cardiolipin in the inner mitochondrial membrane to improve coupling of oxidative phosphorylation and increase ATP synthesis (Birk et al., 2013; Szeto, 2014). They reported that SS31 improved age‐related mitochondrial morphology and glomerulosclerosis in parallel with reduced senescence and increased density of glomerular epithelial and endothelial cells (Sweetwyne et al., 2017). SS31 acts by binding to cardiolipin to preserve mitochondrial cristae ultrastructures, enhance super‐complex formation and electron transfer efficiency, and indirectly reduce ROS. Therefore, it is unclear whether the beneficial effects of SS31 are mediated by reducing production of ROS or through other beneficial effects on mitochondria. In contrast, our findings demonstrate that overexpression of GPX1 enhances scavenging of H_2_O_2_, providing evidence of a direct role of ROS on kidney pathology associated with aging.

To the best of our knowledge, the present study is the first to assess glomerular and tubular proteome changes that are associated with aging and the effects of an antioxidant enzyme, GPX1. As shown in Table S1, expression of several cytoskeletal and focal adhesion proteins is significantly decreased in aged glomeruli, including tensin‐1 (Tns1), plastin‐3, various tubulin subunits, actinin‐4, talin‐1, and podocin (Nphs2). Deletion of the focal adhesion molecule Tns1 in mice caused polycystic kidney disease and glomerulosclerosis (Lo, Yu, Degenstein, Chen, & Fuchs, 1997), suggesting a critical role for Tns1 in maintaining intact cell‐matrix junctions in tubular epithelial cells and podocytes (Nishino et al., 2012). A previous targeted proteomics study in a rat diabetic nephropathy model reported that several proteins involved in the reorganization of the actin cytoskeleton were significantly decreased in the glomeruli, including plastin, tubulin, and actinin (Nakatani et al., 2011). This suggests that aged glomeruli undergo changes in expression of cytoskeletal proteins that are similar to diabetic glomerulosclerosis. Furthermore, podocin (Nphs2) is a critical protein component of podocyte filtration slits, and its deficiency or mutation can cause focal segmental glomerulosclerosis leading to nephrotic syndrome, with massive leakage of albumin into urine (Mollet et al., 2009). In addition, there are significant decreases in glutathione S‐transferase, an enzyme in the glutathione antioxidant system, and in most heat shock proteins/ chaperones. The latter findings suggest glomerular proteostasis is impaired. Consistent with impaired proteostasis, there is a >3‐fold accumulation in the inflammatory markers, S100 and ferritin, and a >9‐fold accumulation of apolipoprotein E (Apo E). Apo E is a protein involved in cholesterol metabolism that accumulates in a wide range of glomerular diseases (Andeen, Yang, Dai, MacCoss, & Smith, 2018). This glomerular proteome remodeling suggests that aging is associated with an increased susceptibility to glomerulosclerosis. Overexpression of GPX1 partially reversed the changes associated with glomerular aging, as reversed expression was observed in ~ 50% of these proteins (i.e., OTG/OWT when compared with OWT/YWT; Figure 6b,c, Table S1).

Targeted proteomics was performed on renal medullary tissues, predominantly tubules and collecting ducts, with a focus on 139 proteins that are involved in metabolism, proteostasis, antioxidants, or mitochondrial and peroxisomal pathways. The pattern of tubular proteome remodeling observed in aged mice is suggestive of a switch in substrate utilization, in which there is an increased use of glycolytic enzymes and a decreased use of fatty acid oxidation enzymes in old mice. Although this novel observation has not been reported in kidneys from aged mice, the switch in substrate utilization from predominantly fatty acids to glucose has been well documented in hearts from aged mice (Dai et al., 2014), heart failure (Dai et al., 2012), and chronic kidney disease (Kang et al., 2015). Interestingly, in contrast to a decrease in heat shock proteins in the glomeruli, we observed a significant increase in heat shock proteins 1 and 90, and Ion peptidases, in tubules (Table S2), which could be a response to increased oxidative damage in kidneys from aged mice. GPX1 TG did not have a protective effect on the switch of substrates that were utilized. However, GPX1 TG mice did attenuate increases in heat shock proteins, in conjunction with better preservation of the antioxidant system and less oxidative damage (Figure 5).

To investigate the relationship between Klotho and ROS, we examined tissue Klotho in GPX1 TG mice and measured oxidative damage in kidneys from 8‐week‐old Klotho‐deficient mice with hypomorphic Klotho allele (Klotho^kl/kl^). Klotho^kl/kl^ mice manifest multiple “accelerated” aging phenotypes, including skin and muscle atrophy, hyperphosphatemia, osteoporosis, vascular calcification, and premature death (Kuro‐o et al., 1997). Immunohistochemistry demonstrated a substantial increase in nitrotyrosine in kidneys from Klotho^kl/kl^ mice (Figure S3), and the increased oxidative damage is consistent with previous reports (Kimura et al., 2018; Yamamoto et al., 2005). The lack of a protective effect on Klotho expression in aged tubular epithelial cells from GPX1 TG mice suggests that reducing ROS does not affect the age‐dependent decline in tissue Klotho expression (Figure S3). Combined with the increased oxidative damage observed in kidneys from Klotho^kl/kl^ (Figure S4), these findings suggest that increased ROS play a role downstream of Klotho deficiency and that loss of Klotho may represent one mechanism underlying kidney aging.

In contrast to the lack of an effect on Klotho expression in GPX1 TG mice, inhibiting mTOR by rapamycin ameliorated vascular disease in CKD by upregulating tissue and secreted Klotho expression, whereas overexpression of mTOR suppressed Klotho gene expression (Zhao et al., 2015). Increased mTOR signaling in kidneys from old rats promoted cellular senescence (Zhuo et al., 2009). Conversely, inhibiting mTOR by rapalogs attenuated aging‐associated nephropathy (Shavlakadze et al., 2018).

We previously demonstrated that inhibiting mTOR by either calorie restriction or rapamycin rejuvenates the aging heart (Dai et al., 2014), the mechanisms of which are not fully understood. Together with the fact that Klotho‐deficient mice had accelerated vascular calcification and exaggerated pathological cardiac hypertrophy (Xie et al., 2012), and that soluble Klotho ameliorated cardiac hypertrophy in heterozygous Klotho‐deficient mice with CKD (Xie, Yoon, An, Kuro‐o, & Huang, 2015), the above findings suggest that age‐related Klotho deficiency may be a mechanism that promotes pathology in both the kidneys and heart. The deficiency of Klotho was reversed by inhibiting mTOR, indicating that mTOR is an upstream regulator of Klotho expression, whereas increased ROS function downstream of both mTOR activation and Klotho deficiency.

In summary, scavenging H_2_O_2_ by modest overexpression of GPX1 attenuates oxidative damage, preserves mitochondrial cristae structure, and ameliorates pathologies and proteome remodeling in the glomeruli and tubules of old mouse kidneys. These beneficial effects are likely downstream and independent of Klotho and Nrf2 signaling. Thus, the present study provides a scientific basis for the potential application of mitochondrial‐targeted antioxidants and mitochondrial protective strategies to delay kidney aging and attenuate chronic kidney diseases.

## 4 EXPERIMENTAL METHODS

Detailed methods are provided in the supplemental material.

### 4.1 Mouse studies

Animal experiments were approved by the University of Iowa Animal Care and Use Committee. The GPX1 transgene comprised 2.2 Kb of 5′ flanking sequence, 1.05 Kb encoding mRNA, and 2.1 Kb of 3′ flanking sequence of the mouse GPX1 locus under its endogenous promoter, as described (Cheng et al., 1997). Klotho‐deficient mice were the result of an insertional mutagenesis screen of the Klotho gene, as described (Kuro‐o et al., 1997). The GPX1 TG mice and mice homozygous for hypomorphic Klotho allele were on the C57BL/6J background. Young (4–5 months old) and old (21–23 months old) littermates were used as controls. All mice were fed with regular chow.

### 4.2 Immunohistochemistry, immunofluorescence, and immunoblotting

For immunohistochemistry and immunofluorescence, kidney slices were embedded in OCT and sections were processed with standard procedures. For immunoblotting, kidneys were homogenized in RIPA containing protease inhibitor cocktail (Roche). After quantification with BCA, equal amount of proteins (30–80 µg) were loaded onto an SDS‐PAGE gel, followed by standard immunoblotting. Band densities were quantified with Image J. Primary antibodies used were anti‐GPX1 (R&D Systems, AF3798, 1:200), nitrotyrosine (MilliporeSigma, #06‐284,1:1000), VDAC1 (Abcam, 15895, 1:100), mKlotho (R&D; AF1819, 1:100), ATP5A (Abcam; ab176569, 1:500), and HSP70 (Cell Signaling Technology, 4872, 1:500). Secondary antibodies were Santa Cruz Biotechnology anti‐goat IgG‐HRP (SC‐2354), anti‐rabbit IgG‐HRP (sc‐2357), anti‐mouse IgG‐HRP (Abcam; ab97046), AlexaFluor 568 donkey anti‐goat IgG, and AlexaFluor 488 goat anti‐rabbit IgG (Thermo Fisher).

### 4.3 Assessment of glomerulosclerosis, interstitial fibrosis, and cortical thickness

Kidney sections (2 µm) were stained with PAS and Masson trichrome. Using PAS staining, we ranked glomeruli on a scale of 0 to 3 for glomerulosclerosis: normal with no injury (score 0); mild mesangiosclerosis: mesangial expansion in <50% with limited Bowman capsule thickening (score 1); moderate mesangiosclerosis: mesangial expansion >50%, with extensive loss of capillary loop structure and podocytes, and more extensive Bowman capsule thickening (score 2); and severe glomerulosclerosis: complete or nearly complete (>75%) glomerulosclerosis with extensive adhesion to Bowman capsules and shrinkage of glomerular tufts with little or no visible/patent capillaries (score 3). The percentage of glomeruli with each score was presented separately as outer cortex and juxtamedullary cortex. Masson trichrome staining was used to assess the degree of interstitial fibrosis. Three representative images at low power from each kidney section were used to quantify the blue area relative to total tissue area by Image J. Cortical thickness was measured at three different sites for each kidney section, at a perpendicular distance from the renal capsule to the medullary regions, which were marked by arrays of tubules with frequent intratubular pink PAS‐positive proteinaceous casts.

### 4.4 Transmission Electron Microscopy

Kidney cortex was fixed in 2.5% glutaraldehyde/cacodylate. Electron microscopy was performed on a JEOL JEM 1200EXII at a magnification of 15,000X. Density of mitochondrial cristae was quantified blindly from 5–8 images, with numbers of cristae normalized to the length of mitochondria.

### 4.5 Ex vivo staining of ROS with fluorescent dyes

Ex vivo staining was performed as previously described (Johnson & Rabinovitch, 2012). Immediately following euthanasia, kidney slices (2–3 mm) were rinsed quickly in HBSS and transferred to DMEM containing 5% FBS, L‐Glutamine, pyruvate, sodium bicarbonate, and gentamycin. Ex vivo fresh kidney slices were stained “live” in the DMEM medium containing 5 µM MitoSOX, 5 µM DCFDA, and 1 µg/ml Hoechst at 37C for 30 min. After washing once with DMEM medium, kidney slices were immediately embedded in OCT and frozen. Frozen kidney slices (5 µm) were sectioned within 3 days, and coverslips were placed over sections with VECTA SHIELD and imaged with a Leica SP8 confocal microscope.

### 4.6 Quantitative RT‐PCR

Total RNA was extracted from kidneys with TriZol and then reverse transcribed. Equal amounts of cDNA were used to quantify gene expression using the TaqMan‐∆∆Ct method, a FAM dye for the gene of interest, and a HEX dye for a ribosomal gene for normalization, all in a single reaction (Chu et al., 2013). Quantitation was performed using the TaqMan Gene Expression Assay (predesigned by Applied Biosystems or Integrated DNA Technologies) on an Applied Biosystems 7900HT Real‐time PCR System. The genes quantified were: Nrf2 (Mm00487471_m1), Gclm (Mm.PT.58.17443532), Nqo1 (Mm00500921_m1) and Hmox1 (Mm00516007_m1). Quantifications were normalized with Rplp0 (Mm.PT.58.43894205).

### 4.7 Measurement of oxidative damage and redox status

Levels of F2‐isoprostanes in kidneys were determined as described (Roberts & Morrow, 2000), with modifications. Briefly, 100 mg of tissue was homogenized in 10 ml of ice‐cold Folch solution (CHCl3: MeOH, 2:1). F2‐isoprostanes were extracted and quantified by gas chromatography‐mass spectrometry using the internal standard [2H4]8‐Iso‐PGF2α, which was added to the samples at the beginning of extraction to correct for yield of the extraction process. Esterified F2‐isoprostanes were measured using gas chromatography–mass spectrometry and calculated as nanograms/gram of tissue.

GSH and GSSG were extracted from tissue homogenate with 5% metaphosphoric acid. Proteins were precipitated on ice for 20 min and pelleted by centrifugation (16,000 *g* for 10 min). Supernatant was filtered (0.45‐μm on syringe) and analyzed for GSH and GSSG with HPLC and electrochemical detection (ESA HPLC system).

### 4.8 Shotgun proteomic analyses of glomeruli

Glomeruli from 18 mice, 6 each from YWT, OWT, and OTG, were harvested by microdissection with a Leica LMD 7000 Laser Microdissection System and processed for reduction, alkylation, and digestion using filter‐aided sample preparation (FASP) (Wisniewski, Zougman, Nagaraj, & Mann, 2009). Trypsin‐digested peptides were cleaned with StageTip (Thermo Fisher Scientific) and loaded for liquid chromatography/MS/MS analysis using higher‐energy collisional dissociation (HCD) on an Orbitrap Fusion Lumos mass spectrometer (Thermo Fisher Scientific) in the top‐speed data‐dependent mode to identify peptides to respective proteins.

### 4.9 Targeted proteomic analysis of tubules

Kidney medulla tissues from 18 mice, 6 each from YWT, OWT, and OTG, were homogenized in RIPA buffer containing protease inhibitor cocktail. One hundred micrograms of total protein from each sample was used for targeted proteomics analysis. Total proteins were mixed with 200 µl of 1% SDS and 20 µl of BSA internal standard. The mixtures were separated on SDS‐PAGE gels, and proteins were extracted from gels. These proteins were determined with a TSQ Quantiva triple quadrupole mass spectrometry system in the selected reaction monitoring mode. The Skyline program was used to determine the integrated area of the appropriate chromatographic peaks for quantification.

### 4.10 Statistical analysis

Data were analyzed using Stata IC10 and presented as means ± *SEM*. Two‐sample *t* test or ANOVA was used to compare differences among groups, followed by post hoc tests for significance. *p* < .05 was considered significant. Proportional odds linear regression (POLR) model (via the polr function in the R library MASS) was used to analyze effects of aging and GPX1 overexpression on glomerular injury score (GS). Briefly, the log odds of GS greater than score *k* is *β*_1_YO + *β*_2_TG − *ζ_k_*, for any *k* = 0, 1, 2, where YO is the dummy variable for old mice, and TG for transgenic mice, and hence, *β*_1_ is the difference in the log odds between young and old mice (i.e., the aging effects) and *β*_2_ is the effect of GPX1 overexpression, given everything else being equal. The *ζ_k_*s are nuisance parameters modeling the baseline distribution of GS.

For shotgun proteomics data, differentially expressed proteins between groups were determined by applying a *t* test with adjusted significance level of *p* < .05 corrected by the Storey *Q*‐values calculated to adjust for false discovery rate. A total of 77 proteins with *p* < .1 between OWT and YWT were used to plot a heatmap. Shotgun proteomics analysis of glomeruli was performed using Partek Genomics Suite 7.0.

Targeted proteomics data were analyzed pathway by pathway on the log scale via a penalized linear regression model that accounts for protein‐specific baselines, protein‐specific aging effects, and protein‐specific effects of GPX1 overexpression; selection of significant effects was enforced by the use of the composite minimax concave penalty with the tuning parameter determined by 10‐fold cross‐validation and other defaults as implemented in the R library grpreg.

## CONFLICT OF INTEREST

The authors do not have any conflicts of interest.

## AUTHOR CONTRIBUTIONS

D.‐F.D. designed and performed the study, analyzed and interpreted the data, and wrote the manuscript. Y.C. performed experiments and wrote the manuscript. R.L. performed shotgun proteomic experiments, data analysis, and interpretation. R.H. and K‐S.C. performed statistical analysis. H.F. and R.K. performed experiments. S.D. assisted in experimental design and manuscript revision.

## Supporting information

Fig S1‐S5

Table S1‐S2

Methods S1

## Data Availability

The data that support the findings of this study are available from the corresponding author upon reasonable request.

## References

[acel13154-bib-0001] USRDS annual data report: Volume one: CKD in the general population (2018). UNITED STATES RENAL DATA SYSTEM.

[acel13154-bib-0002] Andeen, N. K. , Yang, H. Y. , Dai, D. F. , MacCoss, M. J. , & Smith, K. D. (2018). DnaJ homolog subfamily B member 9 is a putative autoantigen in fibrillary GN. Journal of the American Society of Nephrology, 29(1), 231–239. 10.1681/asn.2017050566 29097624 PMC5748922

[acel13154-bib-0003] Birk, A. V. , Liu, S. , Soong, Y. I. , Mills, W. , Singh, P. , Warren, J. D. , … Szeto, H. H. (2013). The mitochondrial‐targeted compound SS‐31 re‐energizes ischemic mitochondria by interacting with cardiolipin. Journal of the American Society of Nephrology, 24(8), 1250–1261. 10.1681/asn.2012121216 23813215 PMC3736700

[acel13154-bib-0004] Bitzer, M. , & Wiggins, J. (2016). Aging biology in the kidney. Advances in Chronic Kidney Disease, 23(1), 12–18. 10.1053/j.ackd.2015.11.005 26709058

[acel13154-bib-0005] Chen, G. , Bridenbaugh, E. A. , Akintola, A. D. , Catania, J. M. , Vaidya, V. S. , Bonventre, J. V. , … Parrish, A. R. (2007). Increased susceptibility of aging kidney to ischemic injury: Identification of candidate genes changed during aging, but corrected by caloric restriction. American Journal of Physiology‐Renal Physiology, 293(4), F1272–1281. 10.1152/ajprenal.00138.2007 17670906 PMC2758575

[acel13154-bib-0006] Cheng, W. H. , Ho, Y. S. , Ross, D. A. , Han, Y. , Combs, G. F. Jr , & Lei, X. G. (1997). Overexpression of cellular glutathione peroxidase does not affect expression of plasma glutathione peroxidase or phospholipid hydroperoxide glutathione peroxidase in mice offered diets adequate or deficient in selenium. Journal of Nutrition, 127(5), 675–680. 10.1093/jn/127.5.675 9164985

[acel13154-bib-0007] Chu, Y. , Lund, D. D. , Weiss, R. M. , Brooks, R. M. , Doshi, H. , Hajj, G. P. , … Heistad, D. D. (2013). Pioglitazone attenuates valvular calcification induced by hypercholesterolemia. Arteriosclerosis, Thrombosis, and Vascular Biology, 33(3), 523–532. 10.1161/atvbaha.112.300794 PMC3573264 23288158

[acel13154-bib-0008] Cogliati, S. , Frezza, C. , Soriano, M. E. , Varanita, T. , Quintana‐Cabrera, R. , Corrado, M. , … Scorrano, L. (2013). Mitochondrial cristae shape determines respiratory chain supercomplexes assembly and respiratory efficiency. Cell, 155(1), 160–171. 10.1016/j.cell.2013.08.032 24055366 PMC3790458

[acel13154-bib-0009] Dai, D.‐F. , Hsieh, E. J. , Liu, Y. , Chen, T. , Beyer, R. P. , Chin, M. T. , … Rabinovitch, P. S. (2012). Mitochondrial proteome remodelling in pressure overload‐induced heart failure: The role of mitochondrial oxidative stress. Cardiovascular Research, 93(1), 79–88. 10.1093/cvr/cvr274 22012956 PMC3243039

[acel13154-bib-0010] Dai, D.‐F. , Karunadharma, P. P. , Chiao, Y. A. , Basisty, N. , Crispin, D. , Hsieh, E. J. , … Rabinovitch, P. S. (2014). Altered proteome turnover and remodeling by short‐term caloric restriction or rapamycin rejuvenate the aging heart. Aging Cell, 13(3), 529–539. 10.1111/acel.12203 24612461 PMC4040127

[acel13154-bib-0011] Dai, D.‐F. , Santana, L. F. , Vermulst, M. , Tomazela, D. M. , Emond, M. J. , MacCoss, M. J. , … Rabinovitch, P. S. (2009). Overexpression of catalase targeted to mitochondria attenuates murine cardiac aging. Circulation, 119(21), 2789–2797. 10.1161/circulationaha.108.822403 19451351 PMC2858759

[acel13154-bib-0012] Glodny, B. , Unterholzner, V. , Taferner, B. , Hofmann, K. J. , Rehder, P. , Strasak, A. , & Petersen, J. (2009). Normal kidney size and its influencing factors – A 64‐slice MDCT study of 1.040 asymptomatic patients. BMC Urology, 9, 19 10.1186/1471-2490-9-19 20030823 PMC2813848

[acel13154-bib-0013] Gouaze, V. , Mirault, M. E. , Carpentier, S. , Salvayre, R. , Levade, T. , & Andrieu‐Abadie, N. (2001). Glutathione peroxidase‐1 overexpression prevents ceramide production and partially inhibits apoptosis in doxorubicin‐treated human breast carcinoma cells. Molecular Pharmacology, 60(3), 488–496. 11502879

[acel13154-bib-0014] Handy, D. E. , Lubos, E. , Yang, Y. I. , Galbraith, J. D. , Kelly, N. , Zhang, Y.‐Y. , … Loscalzo, J. (2009). Glutathione peroxidase‐1 regulates mitochondrial function to modulate redox‐dependent cellular responses. Journal of Biological Chemistry, 284(18), 11913–11921. 10.1074/jbc.M900392200 19254950 PMC2673260

[acel13154-bib-0015] Harman, D. (1972). The biologic clock: The mitochondria? Journal of the American Geriatrics Society, 20(4), 145–147. 10.1111/j.1532-5415.1972.tb00787.x 5016631

[acel13154-bib-0016] Johnson, S. , & Rabinovitch, P. (2012). Ex vivo imaging of excised tissue using vital dyes and confocal microscopy. Current Protocols in Cytometry, 61(1), 1–20, Chapter 9, Unit, 9, 39. 10.1002/0471142956.cy0939s61 PMC3401092 22752953

[acel13154-bib-0017] Kang, H. M. , Ahn, S. H. , Choi, P. , Ko, Y.‐A. , Han, S. H. , Chinga, F. , … Susztak, K. (2015). Defective fatty acid oxidation in renal tubular epithelial cells has a key role in kidney fibrosis development. Nature Medicine, 21(1), 37–46. 10.1038/nm.3762 PMC4444078 25419705

[acel13154-bib-0018] Kimura, T. , Shiizaki, K. , Akimoto, T. , Shinzato, T. , Shimizu, T. , Kurosawa, A. , … Yagisawa, T. (2018). The impact of preserved Klotho gene expression on antioxidative stress activity in healthy kidney. American Journal of Physiology‐Renal Physiology, 315(2), F345–f352. 10.1152/ajprenal.00486.2017 29693450

[acel13154-bib-0019] Kuro‐o, M. , Matsumura, Y. , Aizawa, H. , Kawaguchi, H. , Suga, T. , Utsugi, T. , … Nabeshima, Y.‐I. (1997). Mutation of the mouse klotho gene leads to a syndrome resembling ageing. Nature, 390(6655), 45–51. 10.1038/36285 9363890

[acel13154-bib-0020] Kurosu, H. , Yamamoto, M. , Clark, J. D. , Pastor, J. V. , Nandi, A. , Gurnani, P. , … Kuro‐o, M. (2005). Suppression of aging in mice by the hormone Klotho. Science, 309(5742), 1829–1833. 10.1126/science.1112766 16123266 PMC2536606

[acel13154-bib-0021] Lee, H.‐Y. , Choi, C. S. , Birkenfeld, A. L. , Alves, T. C. , Jornayvaz, F. R. , Jurczak, M. J. , … Shulman, G. I. (2010). Targeted expression of catalase to mitochondria prevents age‐associated reductions in mitochondrial function and insulin resistance. Cell Metabolism, 12(6), 668–674. 10.1016/j.cmet.2010.11.004 21109199 PMC3013349

[acel13154-bib-0022] Li, S. , Yan, T. , Yang, J. Q. , Oberley, T. D. , & Oberley, L. W. (2000). The role of cellular glutathione peroxidase redox regulation in the suppression of tumor cell growth by manganese superoxide dismutase. Cancer Research, 60(14), 3927–3939. 10919671

[acel13154-bib-0023] Lo, S. H. , Yu, Q. C. , Degenstein, L. , Chen, L. B. , & Fuchs, E. (1997). Progressive kidney degeneration in mice lacking tensin. Journal of Cell Biology, 136(6), 1349–1361. 10.1083/jcb.136.6.1349 9087448 PMC2132507

[acel13154-bib-0024] Lorenz, E. C. , Vrtiska, T. J. , Lieske, J. C. , Dillon, J. J. , Stegall, M. D. , Li, X. , … Rule, A. D. (2010). Prevalence of renal artery and kidney abnormalities by computed tomography among healthy adults. Clinical Journal of the American Society of Nephrology, 5(3), 431–438. 10.2215/cjn.07641009 20089492 PMC2827579

[acel13154-bib-0025] Mollet, G. , Ratelade, J. , Boyer, O. , Muda, A. O. , Morisset, L. , Lavin, T. A. , … Esquivel, E. L. (2009). Podocin inactivation in mature kidneys causes focal segmental glomerulosclerosis and nephrotic syndrome. Journal of the American Society of Nephrology, 20(10), 2181–2189. 10.1681/asn.2009040379 19713307 PMC2754108

[acel13154-bib-0026] Nakatani, S. , Kakehashi, A. , Ishimura, E. , Yamano, S. , Mori, K. , Wei, M. , … Wanibuchi, H. (2011). Targeted proteomics of isolated glomeruli from the kidneys of diabetic rats: Sorbin and SH3 domain containing 2 is a novel protein associated with diabetic nephropathy. Experimental Diabetes Research, 2011, 979354 10.1155/2011/979354 22007191 PMC3189611

[acel13154-bib-0027] Neugarten, J. , Gallo, G. , Silbiger, S. , & Kasiske, B. (1999). Glomerulosclerosis in aging humans is not influenced by gender. American Journal of Kidney Diseases, 34(5), 884–888. 10.1016/s0272-6386(99)70046-6 10561145

[acel13154-bib-0028] Nishino, T. , Sasaki, N. , Chihara, M. , Nagasaki, K. , Torigoe, D. , Kon, Y. , & Agui, T. (2012). Distinct distribution of the tensin family in the mouse kidney and small intestine. Experimental Animals, 61(5), 525–532. 10.1538/expanim.61.525 23095816

[acel13154-bib-0029] Olauson, H. , Mencke, R. , Hillebrands, J. L. , & Larsson, T. E. (2017). Tissue expression and source of circulating alphaKlotho. Bone, 100, 19–35. 10.1016/j.bone.2017.03.043 28323144

[acel13154-bib-0030] Roberts, L. J. , & Morrow, J. D. (2000). Measurement of F(2)‐isoprostanes as an index of oxidative stress in vivo. Free Radical Biology and Medicine, 28(4), 505–513. 10.1016/S0891-5849(99)00264-6 10719231

[acel13154-bib-0031] Rule, A. D. , Amer, H. , Cornell, L. D. , Taler, S. J. , Cosio, F. G. , Kremers, W. K. , … Stegall, M. D. (2010). The association between age and nephrosclerosis on renal biopsy among healthy adults. Annals of Internal Medicine, 152(9), 561–567. 10.7326/0003-4819-152-9-201005040-00006 20439574 PMC2864956

[acel13154-bib-0032] Schriner, S. E. , Linford, N. J. , Martin, G. M. , Treuting, P. , Ogburn, C. E. , Emond, M. , … Rabinovitch, P. S. (2005). Extension of murine life span by overexpression of catalase targeted to mitochondria. Science, 308(5730), 1909–1911. 10.1126/science.1106653 15879174

[acel13154-bib-0033] Shavlakadze, T. , Zhu, J. , Wang, S. , Zhou, W. , Morin, B. , Egerman, M. A. , … Glass, D. J. (2018). Short‐term low‐dose mTORC1 inhibition in aged rats counter‐regulates age‐related gene changes and blocks age‐related kidney pathology. Journals of Gerontology. Series A, Biological Sciences and Medical Sciences, 73(7), 845–852. 10.1093/gerona/glx249 PMC6001891 29304191

[acel13154-bib-0034] Sweetwyne, M. T. , Pippin, J. W. , Eng, D. G. , Hudkins, K. L. , Chiao, Y. A. , Campbell, M. D. , … Shankland, S. J. (2017). The mitochondrial‐targeted peptide, SS‐31, improves glomerular architecture in mice of advanced age. Kidney International, 91(5), 1126–1145. 10.1016/j.kint.2016.10.036 28063595 PMC5392164

[acel13154-bib-0035] Szeto, H. H. (2014). First‐in‐class cardiolipin‐protective compound as a therapeutic agent to restore mitochondrial bioenergetics. British Journal of Pharmacology, 171(8), 2029–2050. 10.1111/bph.12461 24117165 PMC3976620

[acel13154-bib-0036] Tonelli, M. , Wiebe, N. , Culleton, B. , House, A. , Rabbat, C. , Fok, M. , … Garg, A. X. (2006). Chronic kidney disease and mortality risk: A systematic review. Journal of the American Society of Nephrology, 17(7), 2034–2047. 10.1681/asn.2005101085 16738019

[acel13154-bib-0037] Vermulst, M. , Wanagat, J. , Kujoth, G. C. , Bielas, J. H. , Rabinovitch, P. S. , Prolla, T. A. , & Loeb, L. A. (2008). DNA deletions and clonal mutations drive premature aging in mitochondrial mutator mice. Nature Genetics, 40(4), 392–394. 10.1038/ng.95 18311139

[acel13154-bib-0038] Wang, X. , Bonventre, J. V. , & Parrish, A. R. (2014). The aging kidney: Increased susceptibility to nephrotoxicity. International Journal of Molecular Sciences, 15(9), 15358–15376. 10.3390/ijms150915358 25257519 PMC4200815

[acel13154-bib-0039] Wisniewski, J. R. , Zougman, A. , Nagaraj, N. , & Mann, M. (2009). Universal sample preparation method for proteome analysis. Nature Methods, 6(5), 359–362. 10.1038/nmeth.1322 19377485

[acel13154-bib-0040] Xie, J. , Cha, S. K. , An, S. W. , Kuro, O. M. , Birnbaumer, L. , & Huang, C. L. (2012). Cardioprotection by Klotho through downregulation of TRPC6 channels in the mouse heart. Nature Communications, 3, 1238 10.1038/ncomms2240 PMC3526952 23212367

[acel13154-bib-0041] Xie, J. , Yoon, J. , An, S. W. , Kuro‐o, M. , & Huang, C. L. (2015). Soluble Klotho protects against uremic cardiomyopathy independently of fibroblast growth factor 23 and phosphate. Journal of the American Society of Nephrology, 26(5), 1150–1160. 10.1681/asn.2014040325 25475745 PMC4413766

[acel13154-bib-0042] Yamamoto, M. , Clark, J. D. , Pastor, J. V. , Gurnani, P. , Nandi, A. , Kurosu, H. , … Kuro‐o, M. (2005). Regulation of oxidative stress by the anti‐aging hormone klotho. Journal of Biological Chemistry, 280(45), 38029–38034. 10.1074/jbc.M509039200 16186101 PMC2515369

[acel13154-bib-0043] Yoshida, T. , Watanabe, M. , Engelman, D. T. , Engelman, R. M. , Schley, J. A. , Maulik, N. , … Das, D. K. (1996). Transgenic mice overexpressing glutathione peroxidase are resistant to myocardial ischemia reperfusion injury. Journal of Molecular and Cellular Cardiology, 28(8), 1759–1767. 10.1006/jmcc.1996.0165 8877785

[acel13154-bib-0044] Zhao, Y. , Zhao, M.‐M. , Cai, Y. , Zheng, M.‐F. , Sun, W.‐L. , Zhang, S.‐Y. , … Xu, M.‐J. (2015). Mammalian target of rapamycin signaling inhibition ameliorates vascular calcification via Klotho upregulation. Kidney International, 88(4), 711–721. 10.1038/ki.2015.160 26061549

[acel13154-bib-0045] Zhuo, L. I. , Cai, G. , Liu, F. , Fu, B. O. , Liu, W. , Hong, Q. , … Chen, X. (2009). Expression and mechanism of mammalian target of rapamycin in age‐related renal cell senescence and organ aging. Mechanisms of Ageing and Development, 130(10), 700–708. 10.1016/j.mad.2009.08.005 19698731

