# Supplementary material for: Glutathione peroxidase‐1 overexpression reduces oxidative stress, and improves pathology and proteome remodeling in the kidneys of old mice
Source: Aging Cell. 2020 May 13;19(6):e13154. doi: 10.1111/acel.13154 (PMC7294784; doi:10.1111/acel.13154)
Supplement: Supplementary file 1 — Fig S1‐S5 [file ACEL-19-e13154-s001.pdf]

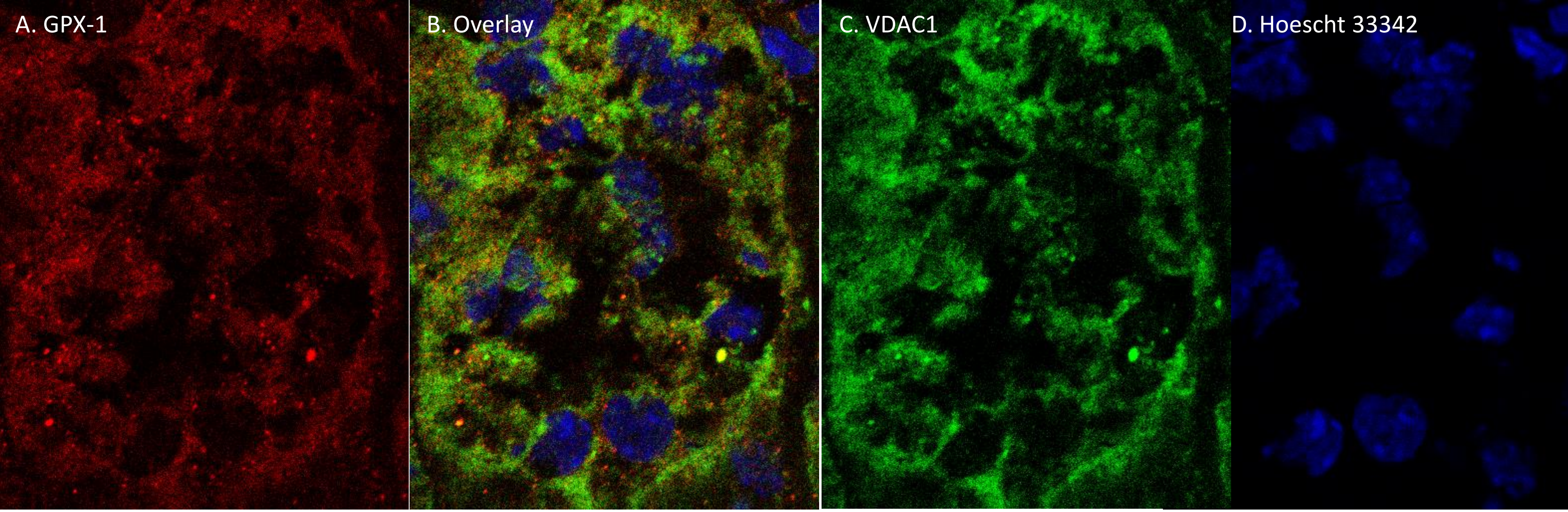

SFigure 1. Immunofluorescence for (A) GPX-1,(C) VDAC1 and (B) overlaid image of kidney tubular epithelial cells. (D) Hoescht 33342 nuclear staining.

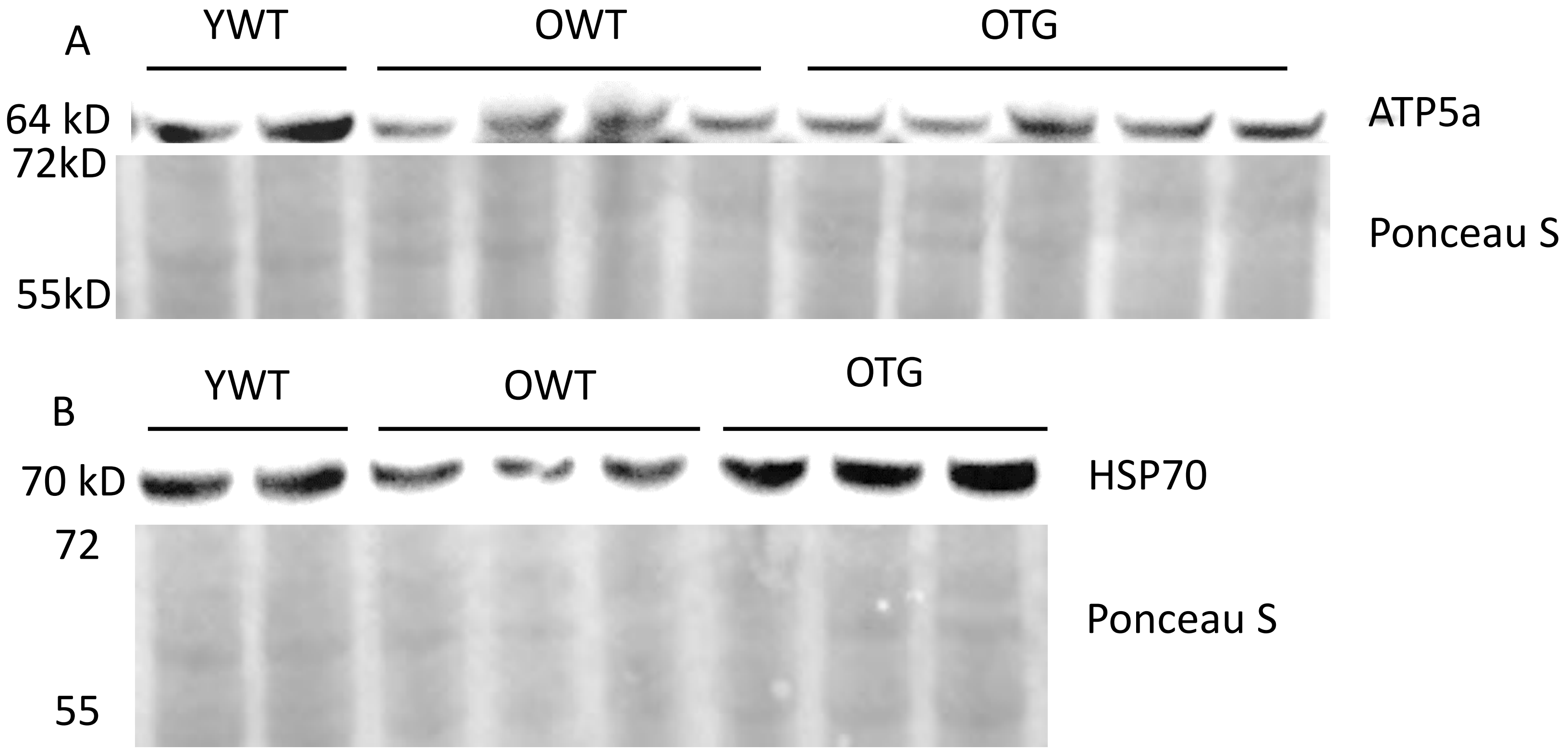

SFigure 2. Western blots of kidney cortex

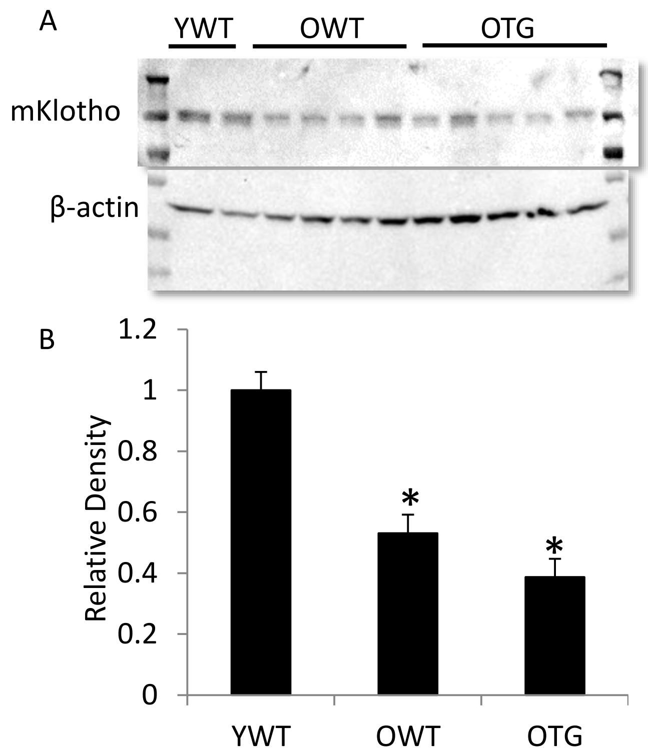

SFig 3. Levels of membrane Klotho (mKlotho) protein in kidneys measured by immunoblotting. A. Immunoblot. B. Quantification of mKlotho after normalization by β-actin. N=3-5. *$p<0.05$ vs. YWT.

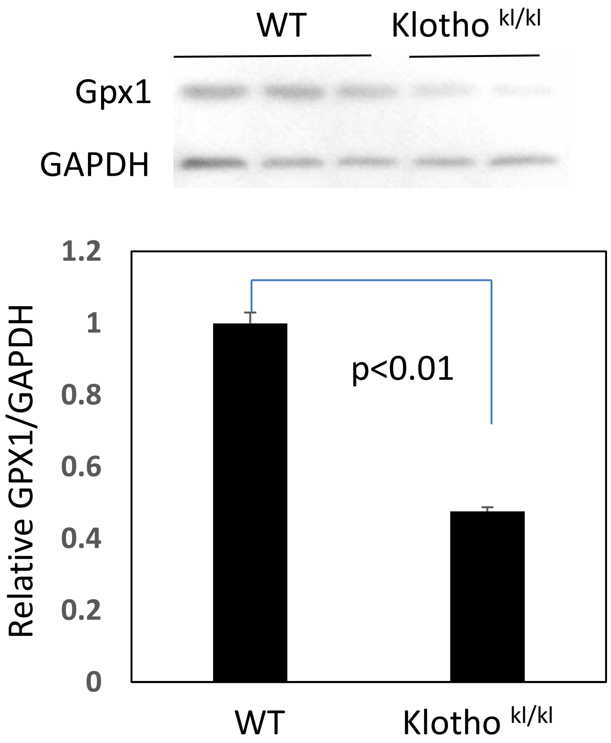

SFig 4. GPX1 protein in kidney lysates from WT and klotho KO mice by immunoblotting.

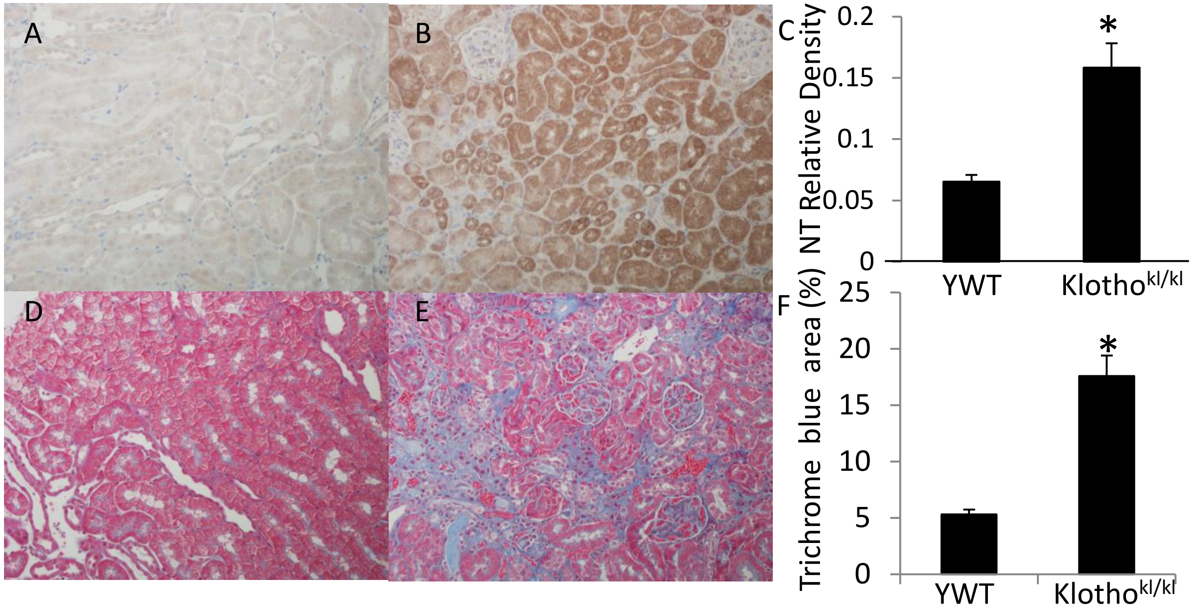

SFigure 5. Immunohistochemistry for Nitrotyrosine (NT) showed that compared with young wild type kidneys(YWT,A), there is substantial increase in NT in Klotho$^{kl/kl}$ kidneys (B), (C). Analysis of anti-Nitrotyrosine staining. Masson trichrome stain showed increased fibrosis in Klotho$^{kl/kl}$ kidneys (E), relative to YWT(D). (F). Analysis of trichrome blue area (%); *$p<0.05$ vs YWT; n=3-5
