# Supplementary material for: Glutathione peroxidase‐1 overexpression reduces oxidative stress, and improves pathology and proteome remodeling in the kidneys of old mice
Source: Aging Cell. 2020 May 13;19(6):e13154. doi: 10.1111/acel.13154 (PMC7294784; doi:10.1111/acel.13154)
Supplement: Supplementary file 2 — Table S1‐S2 [file ACEL-19-e13154-s002.pdf]

STAB.1. Glomerular Proteome Changes in Aging (OWT/YWT) and The Effect of GPX-1 Overexpression (OTG vs.OWT), N=3-4

Probeset ID	Protein names	p-value	q-value	t	MeanRatio (OWT/YWT)	FoldChange (OWT/YWT)	p-value	q-value	t	MeanRatio (OTG/OWT)	FoldChange (OTG/OWT)
Tns1	Tensin 1	3.87E-05	0.0056801	-38.4411	0.0500084	-19.9966	0.355918	0.999478	1	1.18921	1.18921
Ren1	Renin-1;Renin-2;Renin-2 heavy chain;Renin-2 light chain	0.075637	0.456159	-2.67083	0.0747462	-13.3786	0.90745	0.999478	-0.12125	0.874255	-1.14383
Hist1h3e	Histone H3;Histone H3.3;Histone H3.2;Histone H3.1;Histone H3.3C	0.418545	0.624312	-0.93549	0.0839856	-11.9068	0.578777	0.999478	0.586728	4.42061	4.42061
Hist1h1b	Histone H1.5	1.52E-11	6.70E-09	-5251.53	0.0906747	-11.0284	0.754088	0.999478	-0.32796	0.897192	-1.11459
Capns1	Calpain small subunit 1	0.054428	0.453261	-3.07314	0.09634	-10.3799	0.027319	0.999478	2.90043	1.87467	1.87467
Pls3	Plastin-3	0.040309	0.450818	-3.47126	0.103325	-9.67818	0.459873	0.999478	0.789498	1.94556	1.94556
Eef1d	Elongation factor 1-delta	0.006122	0.179581	-6.9453	0.113934	-8.77698	0.670315	0.999478	0.447356	1.43632	1.43632
Synpo	Synaptopodin	0.175478	0.622392	-1.76653	0.115466	-8.66056	0.936875	0.999478	0.082575	1.10084	1.10084
Mecp2	Methyl-CpG Binding Protein 2, DNA methylation, mutation in Rett syndrome	0.008476	0.233097	-6.19416	0.126938	-7.87788	0.039374	0.999478	-2.62402	0.481444	-2.07708
Erh	Enhancer of rudimentary homolog	0.115782	0.572676	-2.19435	0.144399	-6.92524	0.201814	0.999478	-1.43309	0.618564	-1.61665
Nes	Nestin	0.128209	0.587626	-2.08646	0.170369	-5.86963	0.283507	0.999478	-1.17771	0.51171	-1.95423
Atp6v1b1	V-type proton ATPase subunit B, brain isoform	0.272228	0.622392	-1.34164	0.177783	-5.62483	0.246538	0.999478	1.28386	2.6212	2.6212
S100g	Protein S100;Protein S100-G	0.117321	0.572676	-2.18026	0.187669	-5.32853	0.545837	0.999478	-0.63997	0.58176	-1.71892
Rab7	Ras-related protein Rab-7a	0.102292	0.569725	-2.32846	0.194844	-5.13231	?	?	?	1	1
Pcbd1	Pterin-4-alpha-carbinolamine dehydratase	0.054731	0.453261	-3.06607	0.202116	-4.94766	0.622441	0.999478	0.518847	1.22321	1.22321
Col18a1	Collagen alpha-1(XVIII) chain;Endostatin	0.049296	0.453261	-3.20094	0.205343	-4.86991	0.112926	0.999478	1.8555	2.88429	2.88429
Tmod3	Tropomodulin-3	0.234601	0.622392	-1.48342	0.22304	-4.4835	0.78133	0.999478	-0.29034	0.83814	-1.19312
Tubb2b	Tubulin beta-2B chain;Tubulin beta-2A chain	0.146039	0.617856	-1.95165	0.223176	-4.48076	0.137916	0.999478	-1.71107	0.420373	-2.37884
Hnrnpa3	Heterogeneous nuclear ribonucleoprotein A3	0.016074	0.336789	-4.92149	0.230385	-4.34055	0.624329	0.999478	0.515972	1.25022	1.25022
Cct3	T-complex protein 1 subunit gamma	0.000492	0.0308969	-16.4203	0.235557	-4.24525	0.355918	0.999478	1	1.29623	1.29623
Nsf	Vesicle-fusing ATPase	0.075966	0.456159	-2.66574	0.247724	-4.03674	?	?	?	1	1
ATP5F1A	ATP Synthase, H+ Transporting, Mitochondrial F1 Complex, Alpha Subunit,	0.000187	0.0206108	-22.6941	0.253893	-3.93867	0.755399	0.999478	0.326132	1.30224	1.30224
Srsf2	Serine/arginine-rich splicing factor 2	0.07246	0.456159	-2.72146	0.254838	-3.92406	0.355918	0.999478	1	1.41598	1.41598
Pah	Phenylalanine-4-hydroxylase	0.034753	0.413284	-3.68016	0.259099	-3.85953	0.355918	0.999478	1	1.45669	1.45669
Dpp4	Dipeptidyl peptidase 4;Dipeptidyl peptidase 4 membrane form;Dipeptidyl peptidase 4 soluble form	0.000931	0.0455022	-13.2414	0.264199	-3.78503	0.31657	0.999478	-1.09239	0.251772	-3.97184
Col4a4	Collagen alpha-4(IV) chain	0.373171	0.624312	-1.04409	0.270451	-3.69752	0.58397	0.999478	0.578504	2.03522	2.03522
Rpl24	60S ribosomal protein L24	0.124896	0.584618	-2.11397	0.277623	-3.60201	0.654282	0.999478	0.470993	1.35344	1.35344
Nucb2	Nucleobindin-2;Nesfatin-1	0.060778	0.453261	-2.93452	0.27887	-3.58591	0.141678	0.999478	1.69158	2.32198	2.32198
Ehd3	EH domain-containing protein 3	0.155594	0.622392	-1.88714	0.282486	-3.54	0.841948	0.999478	-0.20822	0.885469	-1.12934
Pgam1	Phosphoglycerate mutase 1;Phosphoglycerate mutase 2	0.206772	0.622392	-1.60531	0.284618	-3.51348	0.242822	0.999478	1.29529	2.55251	2.55251
Abhd14b		0.000278	0.0227975	-19.8776	0.28779	-3.47476	0.140498	0.999478	1.69763	1.49849	1.49849
rps14	40S ribosomal protein S14	0.286066	0.622392	-1.2947	0.297009	-3.36691	0.629978	0.999478	0.507403	1.51508	1.51508
Gstk1	Glutathione S-transferase kappa 1	0.272228	0.622392	-1.34164	0.29737	-3.36282	0.046394	0.999478	2.50214	2.6541	2.6541
Cbr1	Carbonyl reductase [NADPH] 1	0.428823	0.624312	-0.91242	0.305152	-3.27706	0.77599	0.999478	0.297674	1.23521	1.23521
Rps11	40S ribosomal protein S11	0.047688	0.453261	-3.24449	0.30672	-3.26031	0.355918	0.999478	1	1.23395	1.23395
Ca2	Carbonic anhydrase 2	0.305913	0.622392	-1.23137	0.306731	-3.26018	0.361804	0.999478	0.986895	2.67564	2.67564
Sh3bgrl3	SH3 domain-binding glutamic acid-rich-like protein 3	0.029543	0.371399	-3.9191	0.310351	-3.22216	0.927222	0.999478	0.095245	1.02541	1.02541
Sri	Sorcin	0.216553	0.622392	-1.5605	0.312312	-3.20193	0.060004	0.999478	-2.31321	0.251707	-3.97288
Clic5	Chloride intracellular channel protein 5;Chloride intracellular channel protein	0.140999	0.616787	-1.9877	0.313961	-3.18511	0.506331	0.999478	0.706572	1.32618	1.32618
Ints7	Integrator complex subunit 7	0.283138	0.622392	-1.30443	0.314523	-3.17942	0.548197	0.999478	-0.63609	0.534117	-1.87225
Ybx1	Nuclease-sensitive element-binding protein 1	0.043261	0.450818	-3.37468	0.316444	-3.16012	0.355918	0.999478	1	1.55545	1.55545
Pgm2	Phosphoglucomutase-1	0.274498	0.622392	-1.33377	0.318092	-3.14374	0.997608	0.999478	-0.00313	0.998044	-1.00196
Sfn	14-3-3 protein sigma	0.308337	0.622392	-1.22392	0.324158	-3.08492	0.718487	0.999478	0.377936	1.59997	1.59997
Fus	RNA-binding protein FUS	0.15886	0.622392	-1.86614	0.336431	-2.97238	0.591704	0.999478	0.566333	1.37646	1.37646
H2-Ke6	Estradiol 17-beta-dehydrogenase 8	0.233292	0.622392	-1.48879	0.339328	-2.947	0.611723	0.999478	-0.53525	0.786837	-1.27091
Hint2	Histidine triad nucleotide-binding protein 2, mitochondrial	0.030537	0.37323	-3.86952	0.346102	-2.88932	0.165057	0.999478	1.58058	1.71858	1.71858
H2afv	Histone H2A;Histone H2A.V;Histone H2A.Z	0.506165	0.625426	-0.75298	0.349029	-2.86509	0.355918	0.999478	-1	0.658831	-1.51784
Sdhb	Succinate dehydrogenase [ubiquinone] iron-sulfur subunit, mitochondrial	0.026429	0.354929	-4.08945	0.356454	-2.80541	?	?	?	1	1

Pcca	Propionyl-CoA carboxylase alpha chain, mitochondrial	0.272228	0.622392	-1.34164	0.36511	-2.7389	0.355918	0.999478	1	1.70507	1.70507
Arpc3	Actin-related protein 2/3 complex subunit 3	0.11844	0.572676	-2.17015	0.375111	-2.66588	0.554251	0.999478	-0.62619	0.770346	-1.29812
Sqrdl	Sulfide:quinone oxidoreductase, mitochondrial	0.084652	0.496624	-2.54037	0.376038	-2.65931	0.355918	0.999478	-1	0.840889	-1.18922
Arpc2	Actin-related protein 2/3 complex subunit 2	0.245377	0.622392	-1.44044	0.379887	-2.63236	0.802458	0.999478	0.261492	1.13571	1.13571
Dusp13		0.365071	0.624312	-1.06479	0.384525	-2.60061	0.041076	0.999478	2.59246	3.74813	3.74813
Aif1l	Allograft inflammatory factor 1-like	0.173697	0.622392	-1.77668	0.390095	-2.56348	0.354077	0.999478	-1.00413	0.669345	-1.494
Lpp	Lipoma-preferred partner homolog	0.331374	0.62345	-1.15603	0.392125	-2.55021	0.684047	0.999478	0.427334	1.33079	1.33079
Eif2a	Eukaryotic translation initiation factor 2A;Eukaryotic translation initiation factor 2A, N-terminally p	0.272228	0.622392	-1.34164	0.395513	-2.52836	0.228259	0.999478	1.34164	2.27042	2.27042
Tln1	Talin-1	0.049967	0.453261	-3.18331	0.398155	-2.51159	0.794119	0.999478	0.272846	1.31754	1.31754
Glod4	Glyoxalase domain-containing protein 4	0.012076	0.279654	-5.45938	0.398915	-2.5068	0.143377	0.999478	1.68293	1.71047	1.71047
Rras2	Ras-related protein R-Ras2;Ras-related protein R-Ras	0.308699	0.622392	-1.22281	0.399105	-2.50561	0.49942	0.999478	-0.71857	0.641933	-1.55779
Gsta1	Glutathione S-transferase A1;Glutathione S-transferase A1, N-terminally processed;Glutathione S-t	0.569317	0.637609	-0.63716	0.403953	-2.47553	0.541106	0.999478	0.647781	1.63222	1.63222
Grhpr	Glyoxylate reductase/hydroxypyruvate reductase	0.388047	0.624312	-1.00717	0.405644	-2.46522	0.03368	0.999478	2.74124	11.5914	11.5914
S100a11	Protein S100-A11	0.191463	0.622392	-1.68044	0.407292	-2.45524	0.307423	0.999478	1.11522	1.84298	1.84298
Anxa1	Annexin;Annexin A1	0.250277	0.622392	-1.42157	0.410375	-2.43679	0.033001	0.999478	-2.75662	0.384723	-2.59927
Slc3a2	4F2 cell-surface antigen heavy chain	0.154311	0.622392	-1.89552	0.413458	-2.41862	0.704991	0.999478	0.397162	1.21094	1.21094
Gm5546		0.272228	0.622392	-1.34164	0.415029	-2.40947	0.355918	0.999478	1	1.23613	1.23613
Msn	Moesin	0.068216	0.456159	-2.79358	0.415182	-2.40858	0.877586	0.999478	0.160723	1.11427	1.11427
Pdia3	Protein disulfide-isomerase A3	0.49982	0.624776	-0.76524	0.416139	-2.40305	0.644508	0.999478	0.485548	1.50401	1.50401
Cryl1	Lambda-crystallin homolog	0.480185	0.624776	-0.80404	0.419365	-2.38456	0.427903	0.999478	-0.85006	0.46608	-2.14556
Ehd4	EH domain-containing protein 4	0.283025	0.622392	-1.30481	0.429131	-2.33029	0.947915	0.999478	0.068105	1.03535	1.03535
Nphs2	Podocin	0.029535	0.371399	-3.91953	0.432125	-2.31414	0.816695	0.999478	0.242195	1.10137	1.10137
Rpl8	60S ribosomal protein L8	0.158653	0.622392	-1.86746	0.435699	-2.29516	0.694334	0.999478	0.412461	1.14281	1.14281
Cyc1	Cytochrome c1, heme protein, mitochondrial	0.280257	0.622392	-1.31411	0.437073	-2.28795	0.704377	0.999478	0.39804	1.27074	1.27074
Enpep	Glutamyl aminopeptidase	0.320796	0.62345	-1.18659	0.443596	-2.25431	0.665921	0.999478	-0.45381	0.693186	-1.44261
Pdhb	Pyruvate dehydrogenase E1 component subunit beta, mitochondrial	0.436665	0.624776	-0.89515	0.444733	-2.24854	0.851079	0.999478	-0.196	0.839903	-1.19061
Bdh2	3-hydroxybutyrate dehydrogenase type 2	0.378462	0.624312	-1.0308	0.44645	-2.23989	0.928061	0.999478	-0.09414	0.913941	-1.09416
Map4	Microtubule-associated protein 4	0.34799	0.62345	-1.10995	0.446693	-2.23867	0.699645	0.999478	-0.40482	0.725059	-1.3792
Hspa12a	Heat shock 70 kDa protein 12A	0.002264	0.0830074	-9.79154	0.447877	-2.23276	0.358967	0.999478	1	1.24874	1.24874
Rps28	40S ribosomal protein S28	0.433645	0.624312	-0.90177	0.447877	-2.23276	0.155733	0.999478	-1.62292	0.266335	-3.75466
Timm13	Mitochondrial import inner membrane translocase subunit Tim13	0.403948	0.624312	-0.96917	0.450778	-2.21838	0.726309	0.999478	0.366867	1.13574	1.13574
Taldo1	Transaldolase	0.001887	0.0754919	-10.4185	0.451307	-2.21579	?	?	?	1	1
Pdlim2	PDZ and LIM domain protein 2	0.158761	0.622392	-1.86677	0.453778	-2.20372	0.257346	0.999478	-1.25146	0.595013	-1.68064
Hnrnpl	Heterogeneous nuclear ribonucleoprotein L	0.475943	0.624776	-0.8126	0.455534	-2.19523	0.446103	0.999478	-0.8152	0.590744	-1.69278
Hmgb1	High mobility group protein B1	0.376222	0.624312	-1.0364	0.461192	-2.1683	0.724977	0.999478	0.368749	1.1834	1.1834
Aldh1l1	10-formyltetrahydrofolate dehydrogenase;Cytosolic 10-formyltetrahydrofolate dehydrogenase	0.49957	0.624776	-0.76573	0.461376	-2.16743	0.555856	0.999478	0.623574	1.69532	1.69532
Myo1c	Unconventional myosin-Ic	0.225802	0.622392	-1.52015	0.464404	-2.1533	0.473736	0.999478	-0.76417	0.509691	-1.96197
Gcdh	Glutaryl-CoA dehydrogenase, mitochondrial	0.412499	0.624312	-0.94931	0.474196	-2.10883	0.822868	0.999478	-0.23386	0.879721	-1.13672
Eprs	Bifunctional glutamate/proline--tRNA ligase;Glutamate--tRNA ligase;Proline--tRNA ligase	0.000311	0.0227975	-19.152	0.474561	-2.10721	?	?	?	1	1
Park7	Protein deglycase DJ-1	0.652438	0.658425	-0.49842	0.477041	-2.09625	0.208712	0.999478	-1.40823	0.213854	-4.67608
Rdx	Radixin	0.162594	0.622392	-1.84274	0.477829	-2.0928	0.212908	0.999478	1.39347	1.52372	1.52372
Slirp	SRA stem-loop-interacting RNA-binding protein, mitochondrial	0.095488	0.552824	-2.4045	0.478655	-2.08919	0.678571	0.999478	0.435294	1.18026	1.18026
Fhl3	Four and a half LIM domains protein 3	0.346286	0.62345	-1.11457	0.480613	-2.08068	0.684891	0.999478	0.42611	1.25561	1.25561
Actr3	Actin-related protein 3	0.350331	0.62345	-1.10363	0.483055	-2.07016	0.675911	0.999478	-0.43917	0.707116	-1.41419
Dync1h1	Cytoplasmic dynein 1 heavy chain 1	0.417333	0.624312	-0.93825	0.484596	-2.06357	0.355918	0.999478	-1	0.840897	-1.18921
Slc9a3r2	Na(+)/H(+) exchange regulatory cofactor NHE-RF;Na(+)/H(+) exchange regulatory cofactor NHE-RF2	0.144384	0.616787	-1.96332	0.48721	-2.0525	0.159053	0.999478	-1.60757	0.627039	-1.5948
Mpeg1	Macrophage-expressed gene 1 protein	0.321226	0.62345	-1.18533	0.490958	-2.03683	0.734121	0.999478	-0.35586	0.762711	-1.31111
Gstp1	Glutathione S-transferase P 1;Glutathione S-transferase P 2	4.28E-06	0.0009408	-80.1762	0.49414	-2.02372	0.355918	0.999478	1	1.27535	1.27535
Anxa5	Annexin A5	0.164772	0.622392	-1.82937	0.494574	-2.02194	0.430488	0.999478	0.845044	1.40277	1.40277
Lasp1	LIM and SH3 domain protein 1	0.054615	0.453261	-3.06877	0.497769	-2.00896	0.896113	0.999478	0.136206	1.11397	1.11397
Dbi	Acyl-CoA-binding protein	0.109331	0.572676	-2.25598	0.499077	-2.0037	0.393968	0.999478	-0.91811	0.804784	-1.24257

Rpl27	60S ribosomal protein L27	0.409365	0.624312	-0.95654	0.499914	-2.00034	0.640788	0.999478	-0.49112	0.76558	-1.3062
Capza2	F-actin-capping protein subunit alpha-2	0.405695	0.624312	-0.96508	0.50054	-1.99784	0.907029	0.999478	0.121807	1.06327	1.06327
Hnrnpa2b1	Heterogeneous nuclear ribonucleoproteins A2/B1	0.076289	0.456159	-2.66077	0.50068	-1.99728	0.783257	0.999478	-0.2877	0.884196	-1.13097
Trap1	Heat shock protein 75 kDa, mitochondrial	0.52408	0.625426	-0.71901	0.504141	-1.98357	0.446929	0.999478	0.81364	1.63841	1.63841
Uqcrfs1	Cytochrome b-c1 complex subunit Rieske, mitochondrial;Cytochrome b-c1 complex subunit 11	0.123835	0.584618	-2.12295	0.504571	-1.98188	0.16169	0.999478	-1.5956	0.524125	-1.90794
Lonp1	Lon protease homolog, mitochondrial	0.272228	0.622392	-1.34164	0.505705	-1.97744	?	?	?	1	1
Rps4x	40S ribosomal protein S4;40S ribosomal protein S4, X isoform	0.408136	0.624312	-0.95939	0.506257	-1.97528	0.602861	0.999478	-0.54894	0.720011	-1.38887
Prdx6	Peroxiredoxin-6	0.346862	0.62345	-1.113	0.513771	-1.94639	0.893705	0.999478	0.139388	1.0746	1.0746
Aldh7a1	Alpha-aminoadipic semialdehyde dehydrogenase	0.127451	0.587626	-2.09268	0.519101	-1.92641	0.557416	0.999478	0.621037	1.45998	1.45998
Cltc	Clathrin heavy chain;Clathrin heavy chain 1	0.237215	0.622392	-1.4728	0.520688	-1.92054	0.699984	0.999478	0.404337	1.27598	1.27598
Pdia6	Protein disulfide-isomerase A6	0.514689	0.625426	-0.7367	0.522713	-1.9131	0.926151	0.999478	0.096651	1.05345	1.05345
Hnrnpc	Heterogeneous nuclear ribonucleoproteins C1/C2	0.313677	0.62345	-1.20773	0.523256	-1.91111	0.355918	0.999478	-1	0.757348	-1.3204
Prkcsh	Glucosidase 2 subunit beta	0.349157	0.62345	-1.10679	0.528807	-1.89105	0.621516	0.999478	0.520256	1.28712	1.28712
Srsf3	Serine/arginine-rich splicing factor 3;Serine/arginine-rich splicing factor 7	0.314831	0.62345	-1.20427	0.531836	-1.88028	0.682775	0.999478	-0.42918	0.819926	-1.21962
Cdh16	Cadherin-16	0.54151	0.625426	-0.68685	0.535194	-1.86848	0.708547	0.999478	0.392081	1.33668	1.33668
Tjp1	Tight junction protein ZO-1	0.539518	0.625426	-0.69048	0.538156	-1.8582	0.96742	0.999478	0.042577	1.02933	1.02933
Rps8	40S ribosomal protein S8	0.131758	0.597666	-2.05792	0.540214	-1.85112	0.140204	0.999478	1.69915	1.51217	1.51217
Capn2	Calpain-2 catalytic subunit	0.042372	0.450818	-3.40285	0.54055	-1.84997	0.235158	0.999478	-1.31936	0.457369	-2.18642
Sord	Sorbitol dehydrogenase	0.412693	0.624312	-0.94886	0.544043	-1.83809	0.921492	0.999478	0.102773	1.08345	1.08345
Arhgdia	Rho GDP-dissociation inhibitor 1	0.339219	0.62345	-1.13399	0.544299	-1.83722	0.810818	0.999478	0.250148	1.29037	1.29037
Gnai2	Guanine nucleotide-binding protein G(i) subunit alpha-2	0.213713	0.622392	-1.57327	0.545624	-1.83276	0.915124	0.999478	0.111148	1.03289	1.03289
Tufm	Elongation factor Tu, mitochondrial	0.408996	0.624312	-0.9574	0.547093	-1.82784	0.21498	0.999478	1.38628	2.21003	2.21003
Pck1	Phosphoenolpyruvate carboxykinase, cytosolic [GTP]	0.724232	0.673321	-0.38752	0.548569	-1.82292	0.261583	0.999478	1.23909	2.19686	2.19686
Tubb5	Tubulin beta-5 chain	0.066081	0.456159	-2.83198	0.549897	-1.81852	0.41771	0.999478	-0.87006	0.776633	-1.28761
Gsta4	Glutathione S-transferase A4	0.053673	0.453261	-3.09096	0.554251	-1.80424	0.183136	0.999478	-1.50457	0.591636	-1.69023
Vcp	Transitional endoplasmic reticulum ATPase	0.371059	0.624312	-1.04944	0.555271	-1.80092	0.941607	0.999478	-0.07637	0.971237	-1.02962
Rps13	40S ribosomal protein S13	0.450914	0.624776	-0.86448	0.556262	-1.79772	0.875268	0.999478	-0.1638	0.890918	-1.12244
Eef2	Elongation factor 2	0.170291	0.622392	-1.79641	0.562345	-1.77827	0.303405	0.999478	1.12542	2.04109	2.04109
Slc5a2	Sodium/glucose cotransporter 2	0.427778	0.624312	-0.91474	0.563929	-1.77327	0.292435	0.999478	-1.15387	0.619187	-1.61502
Slc25a4	ADP/ATP translocase 1	0.582775	0.642019	-0.61377	0.566125	-1.76639	0.292749	0.999478	-1.15305	0.434554	-2.30121
C4b	Complement C4-B;Complement C4 beta chain;Complement C4 alpha chain;C4a anaphylatoxin;Com	0.503983	0.625426	-0.75718	0.57137	-1.75018	0.252527	0.999478	-1.26576	0.520293	-1.92199
Arf4	ADP-ribosylation factor 4	0.508005	0.625426	-0.74944	0.574191	-1.74158	0.769469	0.999478	0.306656	1.22238	1.22238
Septin7	Alpha-1-antitrypsin 1-5	0.019517	0.353248	-4.58209	0.574283	-1.7413	0.424029	0.999478	-0.85762	0.543942	-1.83843
Acss1	Acetyl-coenzyme A synthetase;Acetyl-coenzyme A synthetase 2-like, mitochondrial	0.547745	0.627625	-0.67554	0.574478	-1.74071	0.562301	0.999478	0.613121	1.52289	1.52289
Idh1	Isocitrate dehydrogenase [NADP];Isocitrate dehydrogenase [NADP] cytoplasmic	0.164494	0.622392	-1.83107	0.577224	-1.73243	0.9255	0.999478	-0.09751	0.975266	-1.02536
Rap1b	Ras-related protein Rap-1b;Ras-related protein Rap-1A	0.552344	0.629152	-0.66726	0.577539	-1.73148	0.764911	0.999478	0.31295	1.2397	1.2397
Hspd1	60 kDa heat shock protein, mitochondrial	0.044057	0.450818	-3.35005	0.577882	-1.73046	0.118446	0.999478	1.82106	2.38593	2.38593
Hsp90b1	Heat shock protein HSP 90-beta	0.625877	0.658425	-0.54136	0.580671	-1.72215	0.025989	0.999478	2.93876	3.19975	3.19975
Rpsa	40S ribosomal protein SA	0.219855	0.622392	-1.54588	0.582266	-1.71743	0.220936	0.999478	1.36595	1.75542	1.75542
Clic1	Chloride intracellular channel protein;Chloride intracellular channel protein 1	0.197009	0.622392	-1.65247	0.584021	-1.71227	0.651768	0.999478	-0.47473	0.804431	-1.24311
Nme2	Nucleoside diphosphate kinase;Nucleoside diphosphate kinase B;Nucleoside diphosphate kinase A	0.066074	0.456159	-2.83212	0.591459	-1.69073	0.580389	0.999478	0.584171	1.16086	1.16086
Clic4	Chloride intracellular channel protein;Chloride intracellular channel protein 4	0.113881	0.572676	-2.21208	0.59936	-1.66845	0.854997	0.999478	-0.19077	0.922674	-1.08381
Hist1h2ah	Histone H2A;Histone H2A type 1-H;Histone H2A.J;Histone H2A type 2-C;Histone H2A type 1-K;Histo	0.02662	0.354929	-4.07829	0.600891	-1.6642	0.8	0.999478	-0.26483	0.958498	-1.0433
Tpt1	Translationally-controlled tumor protein	0.476724	0.624776	-0.81102	0.601635	-1.66214	0.355918	0.999478	-1	0.840894	-1.18921
Rhoa	Transforming protein RhoA;Rho-related GTP-binding protein RhoC	0.010424	0.269784	-5.7551	0.603403	-1.65727	0.392678	0.999478	0.920789	1.68956	1.68956
Mdh2	Malate dehydrogenase, mitochondrial	0.251422	0.622392	-1.41722	0.605219	-1.65229	0.460489	0.999478	0.788363	1.36496	1.36496
Parva	Alpha-parvin	0.345059	0.62345	-1.11791	0.606954	-1.64757	0.977873	0.999478	0.028912	1.01189	1.01189
Ppib	Peptidyl-prolyl cis-trans isomerase;Peptidyl-prolyl cis-trans isomerase B	0.272228	0.622392	-1.34164	0.608023	-1.64467	0.135897	0.999478	1.72174	1.68508	1.68508
Acot13	Acyl-coenzyme A thioesterase 13;Acyl-coenzyme A thioesterase 13, N-terminally processed	0.597087	0.647709	-0.58932	0.609031	-1.64195	0.926717	0.999478	0.095908	1.04946	1.04946
Arf5	ADP-ribosylation factor 5	0.420423	0.624312	-0.93124	0.611903	-1.63425	0.355918	0.999478	-1	0.744528	-1.34313
Actbl2	Beta-actin-like protein 2	0.640983	0.658425	-0.5168	0.612077	-1.63378	0.558849	0.999478	-0.61871	0.765275	-1.30672

Rab11b	Ras-related protein Rab-11B;Ras-related protein Rab-11A	0.143351	0.616787	-1.97069	0.615598	-1.62444	0.54823	0.999478	-0.63604	0.770589	-1.29771
Myh11	Myosin-11	0.40664	0.624312	-0.96288	0.620001	-1.6129	0.125872	0.999478	-1.77715	0.350942	-2.84947
Cryz	Quinone oxidoreductase	0.329936	0.62345	-1.16012	0.620004	-1.61289	0.93936	0.999478	-0.07932	0.954449	-1.04773
Ahnak		0.374598	0.624312	-1.04049	0.6241	-1.60231	0.094774	0.999478	-1.9819	0.36398	-2.7474
Naca	Nascent polypeptide-associated complex subunit alpha;Nascent polypeptide-associated complex su	0.142993	0.616787	-1.97325	0.625853	-1.59782	0.679864	0.999478	-0.43341	0.781857	-1.27901
Rbmxl1	RNA binding motif protein, X-linked-like-1;RNA-binding motif protein, X chromosome;RNA-binding	0.316752	0.62345	-1.19854	0.628176	-1.59191	0.730973	0.999478	-0.36029	0.868993	-1.15076
Tpm1	Tropomyosin alpha-1 chain	0.405747	0.624312	-0.96496	0.631017	-1.58474	0.82937	0.999478	0.225102	1.12777	1.12777
Nebl	LIM zinc-binding domain-containing Nebulette	0.304478	0.622392	-1.2358	0.634802	-1.57529	0.719682	0.999478	-0.37624	0.768689	-1.30092
Hsd17b10	3-hydroxyacyl-CoA dehydrogenase type-2	0.272228	0.622392	-1.34164	0.635489	-1.57359	0.355918	0.999478	1	1.20623	1.20623
Fhl2	Four and a half LIM domains protein 2	0.446693	0.624776	-0.87347	0.63699	-1.56988	0.999478	0.999478	0.000682	1.00031	1.00031
Anxa3	Annexin;Annexin A3	0.351306	0.62345	-1.10101	0.637439	-1.56878	0.148337	0.999478	-1.65827	0.64371	-1.5535
Rps3	40S ribosomal protein S3	0.621162	0.658425	-0.54911	0.639428	-1.5639	0.834744	0.999478	0.21788	1.13815	1.13815
Hnrnpab	Heterogeneous nuclear ribonucleoprotein A/B	0.017146	0.342913	-4.80642	0.63968	-1.56328	0.83722	0.999478	-0.21456	0.935571	-1.06887
Sptan1	Spectrin alpha chain, non-erythrocytic 1	0.411115	0.624312	-0.9525	0.639974	-1.56256	0.723641	0.999478	-0.37064	0.828904	-1.20641
Lyrm4	LYR motif-containing protein 4	0.462392	0.624776	-0.84038	0.640372	-1.56159	0.577118	0.999478	0.589366	1.34478	1.34478
Mylk	Myosin light chain kinase, smooth muscle;Myosin light chain kinase, smooth muscle, deglutamylate	0.634965	0.658425	-0.52653	0.641807	-1.5581	0.850625	0.999478	0.196608	1.1737	1.1737
Hsp90ab1	Heat shock protein HSP 90-alpha	0.264947	0.622392	-1.36736	0.642791	-1.55572	0.409437	0.999478	0.886568	1.41123	1.41123
Tbc1d8	TBC1 domain family member 8B;TBC1 domain family member 8	0.541562	0.625426	-0.68675	0.643219	-1.55468	0.355918	0.999478	-1	0.812989	-1.23003
Fxyd2	Sodium/potassium-transporting ATPase subunit gamma	0.700499	0.667141	-0.42344	0.643734	-1.55344	0.961751	0.999478	0.049993	1.04225	1.04225
Gnb1	Guanine nucleotide-binding protein G(I)/G(S)/G(T) subunit beta-1;Guanine nucleotide-binding prot	0.020071	0.353248	-4.53474	0.644565	-1.55143	0.527862	0.999478	0.669875	1.33999	1.33999
Prdx3	Thioredoxin-dependent peroxide reductase, mitochondrial	0.373858	0.624312	-1.04235	0.644673	-1.55117	0.658693	0.999478	0.464461	1.26195	1.26195
Cbx1	Chromobox protein homolog 3;Chromobox protein homolog 1	0.511925	0.625426	-0.74195	0.647039	-1.5455	0.986139	0.999478	0.01811	1.00462	1.00462
Lmnb1	Lamin-B1	0.359002	0.624312	-1.08059	0.649616	-1.53937	0.771051	0.999478	-0.30447	0.853385	-1.1718
Uba1	Ubiquitin-like modifier-activating enzyme 1	0.767292	0.673321	-0.32389	0.650017	-1.53842	0.401956	0.999478	-0.90171	0.49877	-2.00493
Hnrnph2	Heterogeneous nuclear ribonucleoprotein H2;Heterogeneous nuclear ribonucleoprotein H;Heterog	0.272228	0.622392	-1.34164	0.650629	-1.53697	?	?	?	1	1
Actn4	Alpha-actinin-4	0.049693	0.453261	-3.19046	0.651602	-1.53468	0.785188	0.999478	-0.28505	0.914971	-1.09293
Cfl1	Cofilin-1	0.217728	0.622392	-1.55527	0.651858	-1.53408	0.300556	0.999478	-1.13272	0.647841	-1.54359
Hspa1b	Heat shock 70 kDa protein 1A;Heat shock 70 kDa protein 1B	0.272228	0.622392	-1.34164	0.654393	-1.52813	0.506363	0.999478	-0.70652	0.623631	-1.60351
Etfb	Electron transfer flavoprotein subunit beta	0.432843	0.624312	-0.90353	0.657361	-1.52123	0.962805	0.999478	-0.04861	0.977705	-1.0228
Ndufs3	NADH dehydrogenase [ubiquinone] iron-sulfur protein 3, mitochondrial	0.572408	0.63762	-0.63175	0.659553	-1.51618	0.281038	0.999478	1.18441	1.73446	1.73446
Mccc1	Methylcrotonoyl-CoA carboxylase subunit alpha, mitochondrial	0.711049	0.67282	-0.40739	0.659687	-1.51587	0.293022	0.999478	1.15233	1.53834	1.53834
Twf1	Twinfilin-1	0.272228	0.622392	-1.34164	0.664549	-1.50478	0.355918	0.999478	1	1.18921	1.18921
Gstt2	Glutathione S-transferase theta-2	0.520871	0.625426	-0.72503	0.666852	-1.49958	0.511003	0.999478	-0.69852	0.665698	-1.50218
Tpi1	Triosephosphate isomerase	0.526294	0.625426	-0.71488	0.669435	-1.4938	0.742838	0.999478	0.343641	1.2984	1.2984
Bpnt1	3(2),5-bisphosphate nucleotidase 1	0.170184	0.622392	-1.79704	0.669539	-1.49357	0.428135	0.999478	-0.84961	0.754778	-1.32489
Chchd3	MICOS complex subunit Mic19	0.409713	0.624312	-0.95574	0.670585	-1.49124	0.98264	0.999478	-0.02268	0.988145	-1.012
Dpysl2	Dihydropyrimidinase-related protein 2	0.053992	0.453261	-3.08339	0.67173	-1.48869	0.191873	0.999478	-1.47033	0.391066	-2.55711
Akr1b3	Aldose reductase	0.452447	0.624776	-0.86123	0.67757	-1.47586	0.350597	0.999478	-1.01199	0.697545	-1.4336
Pcx	Pyruvate carboxylase;Pyruvate carboxylase, mitochondrial	0.455874	0.624776	-0.854	0.67785	-1.47525	0.431672	0.999478	0.842754	1.49288	1.49288
Fabp4	Fatty acid-binding protein, adipocyte	0.531725	0.625426	-0.7048	0.679337	-1.47202	0.990034	0.999478	0.01302	1.00323	1.00323
Tpm3	Tropomyosin alpha-3 chain	0.4095	0.624312	-0.95623	0.679368	-1.47196	0.904488	0.999478	0.125156	1.04058	1.04058
Upp2	Uridine phosphorylase;Uridine phosphorylase 2	0.534934	0.625426	-0.69888	0.683256	-1.46358	0.224099	0.999478	1.35536	2.85822	2.85822
Smim1	Small integral membrane protein 1	0.686154	0.664995	-0.44549	0.68413	-1.46171	0.49569	0.999478	-0.72509	0.623374	-1.60417
Hist1h1d	Histone H1.3	0.324205	0.62345	-1.17663	0.686576	-1.4565	0.965826	0.999478	-0.04466	0.990971	-1.00911
Ppia	Peptidyl-prolyl cis-trans isomerase;Peptidyl-prolyl cis-trans isomerase A;Peptidyl-prolyl cis-trans iso	0.205043	0.622392	-1.61348	0.688878	-1.45164	0.817079	0.999478	0.241676	1.0464	1.0464
Mif	Macrophage migration inhibitory factor	0.391549	0.624312	-0.99868	0.691322	-1.4465	0.775362	0.999478	-0.29854	0.942382	-1.06114
Pcp4l1	Purkinje cell protein 4-like protein 1	0.752283	0.673321	-0.34586	0.692614	-1.4438	0.925563	0.999478	-0.09742	0.943289	-1.06012
Oxct1	Succinyl-CoA:3-ketoacid-coenzyme A transferase;Succinyl-CoA:3-ketoacid coenzyme A transferase	0.072754	0.456159	-2.71666	0.692984	-1.44303	0.222954	0.999478	1.35918	1.83801	1.83801
Pafah1b1	Platelet-activating factor acetylhydrolase IB subunit alpha	0.56794	0.637609	-0.63958	0.69342	-1.44213	0.395483	0.999478	0.914984	1.52129	1.52129
Pcdhb1		0.775594	0.674789	-0.31182	0.696666	-1.43541	0.973772	0.999478	0.034272	1.02095	1.02095
Pter	Phosphotriesterase-related protein	0.753627	0.673321	-0.34388	0.696868	-1.43499	0.725367	0.999478	0.368197	1.2493	1.2493

Ywhaz	14-3-3 protein zeta/delta	0.074752	0.456159	-2.68466	0.69721	-1.43429	0.855264	0.999478	-0.19041	0.934363	-1.07025
Atp5c1	ATP synthase subunit gamma;ATP synthase subunit gamma, mitochondrial	0.195986	0.622392	-1.65756	0.698697	-1.43124	0.07982	0.999478	2.10593	1.78541	1.78541
Psmb7	Proteasome subunit beta type;Proteasome subunit beta type-7	0.600187	0.647709	-0.58407	0.700233	-1.4281	0.065487	0.999478	2.24949	2.70947	2.70947
Tubb4b	Tubulin beta-4B chain;Tubulin beta-4A chain	0.021042	0.354885	-4.45571	0.701754	-1.425	0.990692	0.999478	0.012161	1.00371	1.00371
Actg1	Actin, cytoplasmic 2;Actin, cytoplasmic 2, N-terminally processed	0.059118	0.453261	-2.96895	0.702175	-1.42415	0.943684	0.999478	-0.07365	0.987166	-1.013
Actn1	Alpha-actinin-1	0.068948	0.456159	-2.78075	0.703072	-1.42233	0.598867	0.999478	-0.55514	0.78292	-1.27727
Ywhae	14-3-3 protein epsilon	0.219803	0.622392	-1.54611	0.705675	-1.41708	0.963828	0.999478	0.047275	1.00833	1.00833
Capg	Macrophage-capping protein	0.272228	0.622392	-1.34164	0.70705	-1.41433	0.034721	0.999478	2.71829	1.97018	1.97018
EG381936		0.272228	0.622392	-1.34164	0.707101	-1.41423	?	?	?	1	1
Fkbp1a	Peptidyl-prolyl cis-trans isomerase;Peptidyl-prolyl cis-trans isomerase FKBP1A	0.272228	0.622392	-1.34164	0.707102	-1.41422	0.134972	0.999478	1.72668	1.45013	1.45013
Pabpc1	Polyadenylate-binding protein;Polyadenylate-binding protein 1	0.272228	0.622392	-1.34164	0.707103	-1.41422	?	?	?	1	1
Rab1b	Ras-related protein Rab-1B	0.272228	0.622392	-1.34164	0.707106	-1.41422	0.355918	0.999478	1	1.78745	1.78745
Mapt	Microtubule-associated protein;Microtubule-associated protein tau	0.272228	0.622392	-1.34164	0.707108	-1.41421	?	?	?	1	1
Ces1d	Carboxylesterase 1D	0.272228	0.622392	-1.34164	0.707109	-1.41421	0.13412	0.999478	1.73127	1.44144	1.44144
Sult1d1	Sulfotransferase;Sulfotransferase 1 family member D1	0.272228	0.622392	-1.34164	0.707111	-1.4142	0.140843	0.999478	1.69586	1.74508	1.74508
Ywhah	14-3-3 protein eta	0.272228	0.622392	-1.34164	0.707114	-1.4142	0.139112	0.999478	1.70481	1.7966	1.7966
C4bp	C4b-binding protein	0.272228	0.622392	-1.34164	0.707115	-1.4142	?	?	?	1	1
Ltf	Lactotransferrin	0.272228	0.622392	-1.34164	0.707119	-1.41419	0.355918	0.999478	1	4.68615	4.68615
Tmsb4x		0.047884	0.453261	-3.2391	0.707451	-1.41353	0.98207	0.999478	0.023427	1.00414	1.00414
Psmb6	Proteasome subunit beta type-6	0.429051	0.624312	-0.91192	0.710063	-1.40833	0.677102	0.999478	-0.43744	0.871828	-1.14702
Fn1	Fibronectin;Anastellin	0.513215	0.625426	-0.7395	0.711258	-1.40596	0.32329	0.999478	-1.07597	0.527576	-1.89546
Ass1	Argininosuccinate synthase	0.641368	0.658425	-0.51617	0.711667	-1.40515	0.900779	0.999478	0.130048	1.09186	1.09186
Tinagl1	Tubulointerstitial nephritis antigen-like	0.069217	0.456159	-2.77607	0.712701	-1.40311	0.792856	0.999478	0.27457	1.09833	1.09833
Rps20	40S ribosomal protein S20	0.554798	0.629152	-0.66286	0.714462	-1.39965	0.653857	0.999478	0.471624	1.31561	1.31561
Podxl	Podocalyxin	0.460255	0.624776	-0.84483	0.714933	-1.39873	0.145304	0.999478	-1.67325	0.652215	-1.53324
Hnrnpu	Heterogeneous nuclear ribonucleoprotein U	0.78576	0.676584	-0.29712	0.716127	-1.3964	0.963092	0.999478	-0.04824	0.965891	-1.03531
Csrp1	Cysteine and glycine-rich protein 1	0.491763	0.624776	-0.78101	0.719038	-1.39075	0.188818	0.999478	-1.48213	0.513385	-1.94786
Septin2	Alpha-1-antitrypsin 1-3;Alpha-1-antitrypsin 1-1;Alpha-1-antitrypsin 1-4;Alpha-1-antitrypsin 1-2	0.527476	0.625426	-0.71268	0.724447	-1.38036	0.165141	0.999478	-1.58021	0.430461	-2.32309
Ptms	Parathymosin	0.638821	0.658425	-0.52029	0.724638	-1.38	0.84392	0.999478	-0.20558	0.942058	-1.06151
Hist1h1e	Histone H1.4	0.196185	0.622392	-1.65657	0.726728	-1.37603	0.682113	0.999478	0.430141	1.12273	1.12273
Ube2v1	Ubiquitin-conjugating enzyme E2 variant 2;Ubiquitin-conjugating enzyme E2 variant 1	0.376375	0.624312	-1.03602	0.728238	-1.37318	0.384145	0.999478	-0.93864	0.745932	-1.3406
Ywhab	14-3-3 protein beta/alpha;14-3-3 protein beta/alpha, N-terminally processed	0.072719	0.456159	-2.71724	0.730552	-1.36883	0.172864	0.999478	-1.54684	0.593529	-1.68484
Hspa9	Stress-70 protein, mitochondrial	0.445028	0.624776	-0.87704	0.731198	-1.36762	0.782004	0.999478	0.289415	1.31512	1.31512
Prph	Peripherin	0.844684	0.67991	-0.21342	0.73267	-1.36487	0.675469	0.999478	-0.43982	0.702344	-1.4238
Asl	Argininosuccinate lyase	0.408377	0.624312	-0.95883	0.734024	-1.36235	0.915795	0.999478	0.110265	1.04155	1.04155
haemaglobin	Hemoglobin subunit alpha	0.522411	0.625426	-0.72213	0.73693	-1.35698	0.12219	0.999478	1.79859	3.72632	3.72632
Esd	S-formylglutathione hydrolase	0.744511	0.673321	-0.35732	0.73903	-1.35313	0.715318	0.999478	0.382436	1.21412	1.21412
Ewsr1	RNA-binding protein EWS	0.588112	0.643705	-0.6046	0.741228	-1.34911	0.791568	0.999478	0.276328	1.09005	1.09005
Ilk	Integrin-linked protein kinase	0.329192	0.62345	-1.16225	0.743128	-1.34566	0.756769	0.999478	-0.32423	0.673588	-1.48459
Atp5k	ATP synthase subunit e, mitochondrial	0.05791	0.453261	-2.99477	0.744964	-1.34235	0.591811	0.999478	0.566165	1.13083	1.13083
Hist1h2bj	Histone H2B;Histone H2B type 1-P;Histone H2B type 1-K;Histone H2B type 1-C/E/G;Histone H2B typ	0.208408	0.622392	-1.59765	0.745503	-1.34138	0.813259	0.999478	-0.24684	0.946736	-1.05626
Acadvl	Very long-chain specific acyl-CoA dehydrogenase, mitochondrial	0.632752	0.658425	-0.53013	0.745789	-1.34086	0.998198	0.999478	0.002354	1.00233	1.00233
Atp5o	ATP synthase subunit O, mitochondrial	0.616277	0.658425	-0.55718	0.747376	-1.33801	0.264539	0.999478	1.23056	1.72093	1.72093
Cpn10-rs1	10 kDa heat shock protein, mitochondrial	0.49353	0.624776	-0.77753	0.747477	-1.33783	0.478513	0.999478	-0.75556	0.722056	-1.38493
Sptbn1	Spectrin beta chain, non-erythrocytic 1	0.423645	0.624312	-0.92398	0.749516	-1.33419	0.971873	0.999478	-0.03675	0.96531	-1.03594
Hbbt1	Hemoglobin subunit beta-1	0.521784	0.625426	-0.72331	0.749974	-1.33338	0.098422	0.999478	1.95465	3.72384	3.72384
Dcxr	L-xylulose reductase	0.192843	0.622392	-1.6734	0.750109	-1.33314	0.332262	0.999478	-1.05448	0.700985	-1.42656
Uqcrc2	Cytochrome b-c1 complex subunit 2, mitochondrial	0.434181	0.624312	-0.90059	0.753603	-1.32696	0.708457	0.999478	0.392209	1.31457	1.31457
Cltb	Clathrin light chain B	0.117575	0.572676	-2.17795	0.754321	-1.32569	0.952894	0.999478	-0.06158	0.981694	-1.01865
Spr	Sepiapterin reductase	0.761278	0.673321	-0.33266	0.754708	-1.32502	0.623537	0.999478	-0.51718	0.80686	-1.23937
Bcam	Basal cell adhesion molecule	0.295515	0.622392	-1.264	0.758109	-1.31907	0.764202	0.999478	-0.31393	0.765557	-1.30624

Fbp1	Fructose-1,6-bisphosphatase 1	0.311196	0.622392	-1.21522	0.758383	-1.3186	0.609693	0.999478	-0.53838	0.837108	-1.19459
Hdgf	Hepatoma-derived growth factor	0.664343	0.658425	-0.47954	0.762069	-1.31222	0.355918	0.999478	-1	0.802416	-1.24624
Gsn	Gelsolin	0.532201	0.625426	-0.70392	0.762151	-1.31208	0.706896	0.999478	0.394438	1.13045	1.13045
Ndufb4	NADH dehydrogenase [ubiquinone] 1 beta subcomplex subunit 4	0.696606	0.666274	-0.4294	0.764182	-1.30859	0.80484	0.999478	-0.25826	0.923987	-1.08227
Miox	Inositol oxygenase	0.840962	0.67991	-0.21864	0.766887	-1.30397	0.225187	0.999478	-1.35175	0.546391	-1.83019
Flna	Filamin-A	0.424521	0.624312	-0.92202	0.770052	-1.29861	0.355467	0.999478	-1.00101	0.307269	-3.25448
Pcbp1	Poly(rC)-binding protein 1	0.788181	0.677343	-0.29363	0.771273	-1.29656	0.631177	0.999478	-0.50559	0.806581	-1.2398
Galm	Aldose 1-epimerase	0.782835	0.675722	-0.30134	0.772452	-1.29458	0.643192	0.999478	-0.48752	0.817799	-1.22279
Cct5	T-complex protein 1 subunit epsilon	0.449589	0.624776	-0.86729	0.773581	-1.29269	0.602821	0.999478	0.548999	1.29785	1.29785
Alpl	Alkaline phosphatase;Alkaline phosphatase, tissue-nonspecific isozyme	0.697589	0.666274	-0.42789	0.775414	-1.28963	0.835587	0.999478	-0.21675	0.911462	-1.09714
Ppp2r1a	Serine/threonine-protein phosphatase 2A 65 kDa regulatory subunit A alpha isoform	0.652559	0.658425	-0.49823	0.779822	-1.28234	0.596403	0.999478	0.558985	1.27083	1.27083
Glud1	Glutamate dehydrogenase 1, mitochondrial	0.468798	0.624776	-0.82716	0.779954	-1.28213	0.391861	0.999478	-0.92248	0.695077	-1.43869
Tagln2	Transgelin-2	0.231883	0.622392	-1.4946	0.781095	-1.28025	0.421295	0.999478	-0.86299	0.839945	-1.19055
Myl6	Myosin light polypeptide 6	0.391565	0.624312	-0.99864	0.785427	-1.27319	0.228878	0.999478	-1.33961	0.390951	-2.55786
Serpinb6a	Serpin B6	0.80624	0.677598	-0.26776	0.786767	-1.27102	0.622528	0.999478	-0.51871	0.798048	-1.25306
Pebp1	Phosphatidylethanolamine-binding protein 1;Hippocampal cholinergic neurostimulating peptide	0.720154	0.673321	-0.39364	0.788207	-1.2687	0.168031	0.999478	1.56755	2.11749	2.11749
Ddx5	Probable ATP-dependent RNA helicase DDX5;Probable ATP-dependent RNA helicase DDX17	0.671703	0.658425	-0.46797	0.788347	-1.26848	0.676258	0.999478	-0.43867	0.816315	-1.22502
Aldoa	Fructose-bisphosphate aldolase;Fructose-bisphosphate aldolase A	0.310438	0.622392	-1.21752	0.788552	-1.26815	0.782954	0.999478	0.288112	1.05031	1.05031
Tkt	Transketolase	0.569501	0.637609	-0.63684	0.791161	-1.26397	0.916769	0.999478	0.108984	1.09697	1.09697
Tpm4	Tropomyosin alpha-4 chain	0.282773	0.622392	-1.30565	0.793443	-1.26033	0.663087	0.999478	-0.45798	0.789079	-1.2673
Vim	Vimentin	0.056943	0.453261	-3.01594	0.797083	-1.25457	0.111644	0.999478	-1.86374	0.545294	-1.83387
Gstm2	Glutathione S-transferase Mu 2	0.112624	0.572676	-2.22399	0.797338	-1.25417	0.935936	0.999478	0.083807	1.02337	1.02337
Gstm5	Glutathione S-transferase Mu 5	0.569002	0.637609	-0.63771	0.800321	-1.2495	0.877231	0.999478	0.161194	1.08377	1.08377
Dab2	Disabled homolog 2	0.807382	0.677598	-0.26613	0.801311	-1.24795	0.598521	0.999478	-0.55568	0.703773	-1.42091
Ogdh	2-oxoglutarate dehydrogenase, mitochondrial	0.670556	0.658425	-0.46977	0.802093	-1.24674	0.972698	0.999478	-0.03568	0.968806	-1.0322
Rpl13	60S ribosomal protein L13	0.136458	0.606482	-2.0215	0.802728	-1.24575	0.526786	0.999478	0.671684	1.1903	1.1903
Hist2h4	Histone H4	0.418284	0.624312	-0.93609	0.808854	-1.23632	0.557779	0.999478	-0.62045	0.834302	-1.19861
Hspg2	Basement membrane-specific heparan sulfate proteoglycan core protein;Endorepellin;LG3 peptide	0.022474	0.354885	-4.34743	0.80901	-1.23608	0.989549	0.999478	0.013654	1.01075	1.01075
Scp2	Non-specific lipid-transfer protein	0.514334	0.625426	-0.73737	0.810337	-1.23405	0.941866	0.999478	-0.07603	0.975348	-1.02528
Psma4	Proteasome subunit alpha type;Proteasome subunit alpha type-4	0.669396	0.658425	-0.47159	0.811318	-1.23256	0.355918	0.999478	-1	0.840902	-1.1892
Atp5j	ATP synthase-coupling factor 6, mitochondrial	0.539528	0.625426	-0.69046	0.817916	-1.22262	0.834977	0.999478	-0.21757	0.930324	-1.07489
Hist1h1c	Histone H1.2	0.805547	0.677598	-0.26875	0.818089	-1.22236	0.191612	0.999478	-1.47133	0.490024	-2.04072
Tgm2	Protein-glutamine gamma-glutamyltransferase 2	0.688807	0.665708	-0.44139	0.819841	-1.21975	0.985766	0.999478	0.018597	1.01038	1.01038
Phb	Prohibitin	0.796273	0.677598	-0.28201	0.823504	-1.21432	0.370406	0.999478	-0.96805	0.582825	-1.71578
Rab21	Ras-related protein Rab-21	0.352816	0.62345	-1.09697	0.824187	-1.21332	0.61854	0.999478	0.5248	1.33019	1.33019
Arf3	ADP-ribosylation factor 1;ADP-ribosylation factor 3;ADP-ribosylation factor 2	0.484972	0.624776	-0.79446	0.824417	-1.21298	0.854972	0.999478	0.190803	1.07979	1.07979
Actr2	Actin-related protein 2	0.224499	0.622392	-1.52572	0.824652	-1.21263	0.572589	0.999478	-0.59659	0.812195	-1.23123
Agrn	Agrin;Agrin N-terminal 110 kDa subunit;Agrin C-terminal 110 kDa subunit;Agrin C-terminal 90 kDa f	0.554302	0.629152	-0.66375	0.82536	-1.21159	0.618724	0.999478	-0.52452	0.687217	-1.45515
Atp6v1g1	V-type proton ATPase subunit G 1	0.416839	0.624312	-0.93937	0.825751	-1.21102	0.283733	0.999478	-1.17709	0.787391	-1.27002
Slc25a13	Calcium-binding mitochondrial carrier protein Aralar2;Calcium-binding mitochondrial carrier protei	0.76919	0.673321	-0.32112	0.82799	-1.20774	0.927855	0.999478	0.094413	1.04379	1.04379
Ezr	Ezrin	0.67189	0.658425	-0.46768	0.829223	-1.20595	0.83053	0.999478	0.223543	1.19591	1.19591
Phb2	Prohibitin-2	0.650263	0.658425	-0.50189	0.831175	-1.20312	0.852067	0.999478	-0.19468	0.898777	-1.11262
Lap3	Cytosol aminopeptidase	0.877993	0.688352	-0.167	0.831851	-1.20214	0.940293	0.999478	0.078093	1.05724	1.05724
Keg1	Glycine N-acyltransferase-like protein Keg1	0.498076	0.624776	-0.76864	0.832167	-1.20168	0.345926	0.999478	1.02264	2.01576	2.01576
Uqcrb	Cytochrome b-c1 complex subunit 7	0.776912	0.674789	-0.30991	0.83253	-1.20116	0.444998	0.999478	-0.81729	0.655794	-1.52487
Lmna	Prelamin-A/C;Lamin-A/C	0.155894	0.622392	-1.88518	0.832852	-1.20069	0.524588	0.999478	-0.67539	0.921677	-1.08498
Dld	Dihydrolipoyl dehydrogenase, mitochondrial;Dihydrolipoyl dehydrogenase	0.362651	0.624312	-1.07106	0.834309	-1.1986	0.179336	0.999478	1.51994	1.44888	1.44888
Dusp3	Dual specificity protein phosphatase 3	0.834204	0.67991	-0.22814	0.834606	-1.19817	0.782485	0.999478	0.288756	1.14927	1.14927
Calm1	Calmodulin-like protein 3	0.729524	0.673321	-0.37959	0.83498	-1.19763	0.445361	0.999478	-0.8166	0.792656	-1.26158
Anpep	Aminopeptidase N	0.764886	0.673321	-0.32739	0.835471	-1.19693	0.898762	0.999478	0.13271	1.11435	1.11435
Pkm	Pyruvate kinase PKM	0.5394	0.625426	-0.6907	0.836609	-1.1953	0.301348	0.999478	1.13069	1.4269	1.4269

Atp5f1	ATP synthase F(0) complex subunit B1, mitochondrial	0.814972	0.67991	-0.25533	0.83768	-1.19377	0.795476	0.999478	0.270995	1.24343	1.24343
Cct7	T-complex protein 1 subunit eta	0.707182	0.670603	-0.41326	0.838799	-1.19218	0.739614	0.999478	0.348154	1.12525	1.12525
Hspa5	78 kDa glucose-regulated protein	0.423268	0.624312	-0.92483	0.843466	-1.18558	0.818228	0.999478	-0.24012	0.944331	-1.05895
Vcl	Vinculin	0.180754	0.622392	-1.73716	0.84407	-1.18474	0.507188	0.999478	-0.70509	0.818173	-1.22224
Ywhag	14-3-3 protein gamma;14-3-3 protein gamma, N-terminally processed	0.304418	0.622392	-1.23598	0.844824	-1.18368	0.822855	0.999478	0.233878	1.09408	1.09408
Rpl22	60S ribosomal protein L22	0.237182	0.622392	-1.47293	0.847104	-1.18049	0.542439	0.999478	-0.64558	0.805097	-1.24209
Cyb5r3	NADH-cytochrome b5 reductase;NADH-cytochrome b5 reductase 3;NADH-cytochrome b5 reductas	0.838048	0.67991	-0.22273	0.848759	-1.17819	0.682156	0.999478	-0.43008	0.849997	-1.17647
Ndrg1	Nucleoside diphosphate kinase;Nucleoside diphosphate kinase 3	0.598994	0.647709	-0.58609	0.849	-1.17786	0.304305	0.999478	1.12312	2.08694	2.08694
Atp6v1a	V-type proton ATPase catalytic subunit A	0.561416	0.635021	-0.65108	0.849718	-1.17686	0.998739	0.999478	0.001648	1.00044	1.00044
Lman1	Protein ERGIC-53	0.724262	0.673321	-0.38747	0.850449	-1.17585	0.193423	0.999478	-1.46441	0.534589	-1.87059
Prx	Periaxin	0.729139	0.673321	-0.38017	0.853684	-1.17139	0.659087	0.999478	0.463878	1.20244	1.20244
Coq9	Ubiquinone biosynthesis protein COQ9, mitochondrial	0.063149	0.456159	-2.88736	0.854259	-1.1706	0.716988	0.999478	-0.38006	0.861033	-1.1614
Cct8	T-complex protein 1 subunit theta	0.777541	0.674789	-0.309	0.854435	-1.17036	0.742824	0.999478	-0.34366	0.838989	-1.19191
Atp5j2	ATP synthase subunit f, mitochondrial	0.511246	0.625426	-0.74325	0.854908	-1.16972	0.743953	0.999478	0.342082	1.18225	1.18225
Eppk1	Epiplakin	0.431718	0.624312	-0.90601	0.85617	-1.16799	0.16305	0.999478	1.5895	1.94168	1.94168
Sod1	Superoxide dismutase [Cu-Zn]	0.660377	0.658425	-0.48581	0.856595	-1.16741	0.341156	0.999478	-1.03364	0.740359	-1.3507
Atp2a2	Sarcoplasmic/endoplasmic reticulum calcium ATPase 2;Sarcoplasmic/endoplasmic reticulum calciu	0.797112	0.677598	-0.28081	0.857449	-1.16625	0.735697	0.999478	-0.35365	0.75814	-1.31902
Capzb	F-actin-capping protein subunit beta	0.264865	0.622392	-1.36766	0.858128	-1.16533	0.663998	0.999478	-0.45663	0.896198	-1.11582
Shmt2	Serine hydroxymethyltransferase	0.810349	0.677859	-0.2619	0.859118	-1.16398	0.993658	0.999478	-0.00829	0.993753	-1.00629
Acaa2	3-ketoacyl-CoA thiolase, mitochondrial	0.731936	0.673321	-0.37599	0.861495	-1.16077	0.776671	0.999478	-0.29674	0.887154	-1.1272
Hoga1	4-hydroxy-2-oxoglutarate aldolase, mitochondrial	0.915948	0.697262	-0.11467	0.86314	-1.15856	0.86807	0.999478	0.173359	1.25105	1.25105
Rps18	40S ribosomal protein S18	0.826003	0.67991	-0.23971	0.863681	-1.15784	0.774239	0.999478	0.300082	1.23892	1.23892
Hnrnpk	Heterogeneous nuclear ribonucleoprotein K	0.391205	0.624312	-0.99951	0.864238	-1.15709	0.543829	0.999478	-0.64328	0.729217	-1.37133
Decr1	2,4-dienoyl-CoA reductase, mitochondrial	0.551605	0.629152	-0.66859	0.865114	-1.15592	0.892148	0.999478	-0.14145	0.942431	-1.06109
EG433182	Alpha-enolase	0.680486	0.662255	-0.45427	0.867477	-1.15277	0.586747	0.999478	0.574122	1.15689	1.15689
Cs	Citrate synthase, mitochondrial;Citrate synthase	0.746783	0.673321	-0.35396	0.867757	-1.1524	0.317839	0.999478	1.08927	2.26422	2.26422
Gcc2	GRIP and coiled-coil domain-containing protein 2	0.862146	0.68459	-0.18901	0.868324	-1.15164	0.53045	0.999478	0.66553	1.4127	1.4127
Eef1a1	Elongation factor 1-alpha;Elongation factor 1-alpha 1;Elongation factor 1-alpha 2	0.405018	0.624312	-0.96666	0.881411	-1.13454	0.27124	0.999478	1.21152	1.19483	1.19483
Hspa8	Heat shock cognate 71 kDa protein	0.582921	0.642019	-0.61351	0.882779	-1.13279	0.878044	0.999478	-0.16012	0.980668	-1.01971
Lama5	Laminin subunit alpha-5	0.63307	0.658425	-0.52961	0.883887	-1.13137	0.668872	0.999478	-0.44947	0.753237	-1.3276
Hrsp12	Ribonuclease UK114	0.439778	0.624776	-0.88838	0.885406	-1.12943	0.170636	0.999478	-1.55632	0.647696	-1.54393
Trrap	Transformation/transcription domain-associated protein	0.779749	0.675373	-0.3058	0.885525	-1.12927	0.134444	0.999478	-1.72952	0.697415	-1.43387
Hnrnpf	Heterogeneous nuclear ribonucleoprotein F;Heterogeneous nuclear ribonucleoprotein F, N-termina	0.783223	0.675722	-0.30078	0.887599	-1.12663	0.355918	0.999478	-1	0.840891	-1.18921
Snrpd3	Small nuclear ribonucleoprotein Sm D3	0.763815	0.673321	-0.32896	0.887694	-1.12651	0.494831	0.999478	-0.7266	0.848604	-1.17841
Col6a3		0.877496	0.688352	-0.16769	0.891946	-1.12114	0.752193	0.999478	0.330591	1.16086	1.16086
Cotl1	Coactosin-like protein	0.845251	0.67991	-0.21262	0.893889	-1.11871	0.870229	0.999478	0.17049	1.08596	1.08596
Sfxn1	Sideroflexin-1	0.943767	0.704767	-0.0766	0.896541	-1.1154	0.805179	0.999478	-0.2578	0.802968	-1.24538
Dak	Bifunctional ATP-dependent dihydroxyacetone kinase/FAD-AMP lyase (cyclizing);ATP-dependent di	0.908981	0.695568	-0.12424	0.90729	-1.10218	0.928016	0.999478	-0.0942	0.957209	-1.0447
Tuba1b	Tubulin alpha-1B chain;Tubulin alpha-1C chain;Tubulin alpha-1A chain;Tubulin alpha-3 chain;Tubuli	0.349352	0.62345	-1.10627	0.907921	-1.10142	0.832973	0.999478	-0.22026	0.961065	-1.04051
Rpl18	60S ribosomal protein L18	0.675618	0.660372	-0.46185	0.907943	-1.10139	0.08915	0.999478	2.02606	1.36615	1.36615
Prdx5	Peroxiredoxin-5, mitochondrial	0.633636	0.658425	-0.52869	0.908607	-1.10059	0.630653	0.999478	0.506382	1.19543	1.19543
Gpx1	Glutathione peroxidase 1;Glutathione peroxidase	0.671893	0.658425	-0.46768	0.909022	-1.10008	0.061828	0.999478	2.29137	2.22819	2.22819
Dctn1	Dynactin subunit 1	0.844861	0.67991	-0.21317	0.914272	-1.09377	0.422565	0.999478	0.860491	1.34121	1.34121
Gm4450	3 beta-hydroxysteroid dehydrogenase type 4;3 beta-hydroxysteroid dehydrogenase type 5	0.894451	0.691667	-0.14425	0.917401	-1.09004	0.14172	0.999478	-1.69136	0.663205	-1.50783
GAPDH	Glyceraldehyde-3-phosphate dehydrogenase	0.667703	0.658425	-0.47425	0.918699	-1.0885	0.346546	0.999478	1.02122	1.19383	1.19383
Rplp2	60S acidic ribosomal protein P2	0.927428	0.699817	-0.09894	0.920652	-1.08619	0.097172	0.999478	-1.96388	0.301353	-3.31837
Eif4a1	Eukaryotic initiation factor 4A-I;Eukaryotic initiation factor 4A-I;Eukaryotic initiation factor 4A-II, N	0.597888	0.647709	-0.58796	0.930139	-1.07511	0.266747	0.999478	-1.22424	0.908005	-1.10132
Myh9	Myosin-9	0.60038	0.647709	-0.58375	0.930728	-1.07443	0.744943	0.999478	-0.3407	0.949843	-1.05281
Anxa6	Annexin;Annexin A6	0.85678	0.684534	-0.1965	0.931481	-1.07356	0.444569	0.999478	-0.8181	0.675066	-1.48134
Ctsd	Cathepsin D	0.929694	0.699817	-0.09584	0.934958	-1.06957	0.366653	0.999478	0.976228	1.65687	1.65687
Gm6793		0.893081	0.691667	-0.14614	0.936338	-1.06799	0.412212	0.999478	-0.881	0.616702	-1.62153

Ndufa12	NADH dehydrogenase [ubiquinone] 1 alpha subcomplex subunit 12	0.935724	0.701395	-0.08759	0.939162	-1.06478	0.161177	0.999478	-1.59791	0.611345	-1.63574
Ywhaq	14-3-3 protein theta	0.804651	0.677598	-0.27002	0.941512	-1.06212	0.160196	0.999478	-1.60236	0.757004	-1.321
Aco2	Aconitate hydratase, mitochondrial	0.879003	0.688352	-0.1656	0.945249	-1.05792	0.619976	0.999478	0.522606	1.22921	1.22921
Slc9a3r1	Na(+)/H(+) exchange regulatory cofactor NHE-RF;Na(+)/H(+) exchange regulatory cofactor NHE-RF1	0.858813	0.684534	-0.19366	0.946335	-1.05671	0.447872	0.999478	-0.81187	0.567532	-1.76202
Ubc	Ubiquitin-60S ribosomal protein L40;Ubiquitin;60S ribosomal protein L40;Ubiquitin-40S ribosomal p	0.748053	0.673321	-0.35209	0.947545	-1.05536	0.41534	0.999478	-0.87477	0.862249	-1.15976
Ldhb	L-lactate dehydrogenase B chain;L-lactate dehydrogenase	0.71468	0.673321	-0.4019	0.951429	-1.05105	0.940416	0.999478	0.077933	1.01866	1.01866
H2afy	Core histone macro-H2A.1;Histone H2A;Core histone macro-H2A;Core histone macro-H2A.2	0.891339	0.691667	-0.14854	0.952445	-1.04993	0.504058	0.999478	-0.71051	0.907965	-1.10136
Cnn3	Calponin-3	0.962586	0.705896	-0.05093	0.953598	-1.04866	0.559372	0.999478	-0.61786	0.567379	-1.76249
Atp6v1e1	V-type proton ATPase subunit E 1	0.801681	0.677598	-0.27427	0.958916	-1.04284	0.801776	0.999478	0.262419	1.23705	1.23705
mt-Co2	Cytochrome c oxidase subunit 2	0.887554	0.689971	-0.15377	0.961631	-1.0399	0.400754	0.999478	0.904162	1.48562	1.48562
Actc1	Actin, alpha cardiac muscle 1;Actin, aortic smooth muscle;Actin, gamma-enteric smooth muscle	0.928865	0.699817	-0.09697	0.961838	-1.03968	0.944474	0.999478	-0.07261	0.971351	-1.02949
Marcks	Myristoylated alanine-rich C-kinase substrate	0.961784	0.705896	-0.05202	0.96681	-1.03433	0.510724	0.999478	-0.699	0.721255	-1.38647
Txn	Thioredoxin	0.833622	0.67991	-0.22896	0.969757	-1.03119	0.843878	0.999478	0.205631	1.13346	1.13346
H1f0	Histone H1.0;Histone H1.0, N-terminally processed	0.981476	0.712149	-0.0252	0.970385	-1.03052	0.503206	0.999478	-0.71198	0.517712	-1.93158
Atp5a1	ATP synthase subunit alpha, mitochondrial;ATP synthase subunit alpha	0.907662	0.695568	-0.12606	0.972791	-1.02797	0.434685	0.999478	0.836946	1.28484	1.28484
mt-Co3	Cytochrome c oxidase subunit 3	0.931921	0.699817	-0.09279	0.974282	-1.0264	0.855504	0.999478	-0.19009	0.84389	-1.18499
Des	Desmin	0.918056	0.697659	-0.11178	0.977233	-1.0233	0.165597	0.999478	-1.5782	0.413546	-2.41811
Idh2	Isocitrate dehydrogenase [NADP], mitochondrial	0.954834	0.705807	-0.06149	0.984397	-1.01585	0.969771	0.999478	0.039502	1.01146	1.01146
Rps26	40S ribosomal protein S26	0.982441	0.712149	-0.02389	0.98884	-1.01129	0.51204	0.999478	0.696743	1.34741	1.34741
Gatm	Glycine amidinotransferase, mitochondrial	0.989911	0.716383	-0.01373	0.989095	-1.01102	0.484356	0.999478	-0.74511	0.60841	-1.64363
Sdha	Succinate dehydrogenase [ubiquinone] flavoprotein subunit, mitochondrial	0.95544	0.705807	-0.06067	0.989825	-1.01028	0.505262	0.999478	0.70842	1.37536	1.37536
Pof1b	Protein POF1B	0.99693	0.716612	-0.00418	0.992914	-1.00714	0.678321	0.999478	-0.43566	0.597783	-1.67285
Aass	Alpha-aminoadipic semialdehyde synthase, mitochondrial;Lysine ketoglutarate reductase;Saccharo	0.993061	0.716612	-0.00944	0.995186	-1.00484	0.315797	0.999478	1.0943	1.88934	1.88934
Hsd17b4	Peroxisomal multifunctional enzyme type 2;(3R)-hydroxyacyl-CoA dehydrogenase;Enoyl-CoA hydra	1	0.716612	-1.00E-08	1	-1	0.355918	0.999478	1	1.1892	1.1892
Gm11214		?	?	?	1	1	0.355918	0.999478	1	1.90709	1.90709
Spata5	Spermatogenesis-associated protein 5;Katanin p60 ATPase-containing subunit A-like 2	?	?	?	1	1	0.589857	0.999478	-0.56923	0.533238	-1.87533
Hnrnpa1	Heterogeneous nuclear ribonucleoprotein A1;Heterogeneous nuclear ribonucleoprotein A1, N-term	0.999289	0.716612	0.000968	1.00032	1.00032	0.102451	0.999478	-1.92572	0.392581	-2.54724
Pan2	PAB-dependent poly(A)-specific ribonuclease subunit PAN2	0.99773	0.716612	0.003087	1.00256	1.00256	0.394882	0.999478	0.916226	2.04141	2.04141
Csrp2	Cysteine and glycine-rich protein 2	0.995945	0.716612	0.005516	1.00679	1.00679	0.223616	0.999478	-1.35697	0.454182	-2.20176
Ahcy	Adenosylhomocysteinase	0.94663	0.704767	0.072687	1.00996	1.00996	0.722983	0.999478	0.371567	1.11613	1.11613
Mdh1	Malate dehydrogenase, cytoplasmic	0.921534	0.698182	0.107013	1.01168	1.01168	0.838045	0.999478	0.213449	1.05606	1.05606
Snrpa	U1 small nuclear ribonucleoprotein A	0.897927	0.693137	0.139454	1.01538	1.01538	0.025694	0.999478	-2.94757	0.540517	-1.85008
Cox6b1	Cytochrome c oxidase subunit 6B1	0.973406	0.711768	0.036188	1.01713	1.01713	0.693743	0.999478	-0.41331	0.791829	-1.2629
Ndufs1	NADH-ubiquinone oxidoreductase 75 kDa subunit, mitochondrial	0.977	0.711999	0.031295	1.01759	1.01759	0.728003	0.999478	-0.36448	0.716061	-1.39653
Arpc1b	Actin-related protein 2/3 complex subunit 1B	0.977381	0.711999	0.030776	1.02148	1.02148	0.164016	0.999478	-1.58519	0.60454	-1.65415
Idh3a	Isocitrate dehydrogenase [NAD] subunit alpha, mitochondrial	0.921917	0.698182	0.106487	1.02778	1.02778	0.678034	0.999478	-0.43608	0.779179	-1.2834
Picalm	Phosphatidylinositol-binding clathrin assembly protein	0.979412	0.712149	0.028012	1.02935	1.02935	0.545099	0.999478	-0.64119	0.711213	-1.40605
Ndufv1	NADH dehydrogenase [ubiquinone] flavoprotein 1, mitochondrial	0.932029	0.699817	0.09264	1.02957	1.02957	0.45778	0.999478	-0.79337	0.577812	-1.73067
Mfge8	Lactadherin	0.960305	0.705896	0.054034	1.03018	1.03018	0.139757	0.999478	1.70146	2.20738	2.20738
Cox4i1	Cytochrome c oxidase subunit 4 isoform 1, mitochondrial	0.912499	0.695907	0.119408	1.03069	1.03069	0.812897	0.999478	0.247332	1.10529	1.10529
Aldh6a1	Methylmalonate-semialdehyde dehydrogenase [acylating], mitochondrial	0.912588	0.695907	0.119287	1.03104	1.03104	0.701433	0.999478	0.402257	1.18602	1.18602
Sorbs1	Sorbin and SH3 domain-containing protein 1	0.961878	0.705896	0.051891	1.03162	1.03162	0.59219	0.999478	0.565572	1.29039	1.29039
Kpnb1	Importin subunit beta-1	0.658162	0.658425	0.489316	1.03197	1.03197	0.214689	0.999478	-1.38728	0.853585	-1.17153
Dstn	Destrin	0.660119	0.658425	0.486214	1.03389	1.03389	0.496468	0.999478	0.723728	1.22542	1.22542
mCG_6739	40S ribosomal protein S21	0.731671	0.673321	0.376389	1.03413	1.03413	0.011451	0.999478	-3.59384	0.487772	-2.05014
Glyat	Glycine N-acyltransferase	0.877983	0.688352	0.167013	1.03511	1.03511	0.872851	0.999478	-0.16701	0.897637	-1.11404
Myl12a	Myosin regulatory light chain 12B	0.887041	0.689971	0.154478	1.0423	1.0423	0.131257	0.999478	-1.74687	0.46318	-2.15899
Immt	MICOS complex subunit Mic60	0.94879	0.705182	0.069739	1.0453	1.0453	0.768819	0.999478	0.307553	1.28093	1.28093
Lamc1	Laminin subunit gamma-1	0.832756	0.67991	0.230178	1.04621	1.04621	0.441457	0.999478	-0.824	0.645611	-1.54892
Akr1a1	Alcohol dehydrogenase [NADP(+)]	0.712818	0.673047	0.40471	1.04872	1.04872	0.815846	0.999478	0.243343	1.09963	1.09963
Aldh8a1	Aldehyde dehydrogenase family 8 member A1	0.973828	0.711768	0.035613	1.05038	1.05038	0.792784	0.999478	-0.27467	0.795297	-1.25739

Map4k1	Mitogen-activated protein kinase kinase kinase kinase;Mitogen-activated protein kinase kinase kina	0.765029	0.673321	0.327185	1.05043	1.05043	0.849514	0.999478	-0.19809	0.927006	-1.07874
Serpina3k	Serpin B5	0.956047	0.705807	0.059839	1.0528	1.0528	0.674655	0.999478	0.441007	1.55947	1.55947
Aldob	Fructose-bisphosphate aldolase;Fructose-bisphosphate aldolase B	0.739808	0.673321	0.364285	1.06326	1.06326	0.947195	0.999478	-0.06905	0.981089	-1.01928
Vil1	Villin-1	0.852919	0.683578	0.201888	1.0698	1.0698	0.690026	0.999478	0.418677	1.28503	1.28503
Ptma	Prothymosin alpha;Prothymosin alpha, N-terminally processed;Thymosin alpha	0.824	0.67991	0.242537	1.07406	1.07406	0.177465	0.999478	-1.52762	0.254187	-3.93411
Ddx3y	ATP-dependent RNA helicase DDX3Y;Putative ATP-dependent RNA helicase Pl10;ATP-dependent RI	0.833368	0.67991	0.229315	1.07566	1.07566	0.370127	0.999478	0.968654	1.50193	1.50193
Myh10	Myosin-10	0.400652	0.624312	0.976929	1.07676	1.07676	0.78059	0.999478	-0.29136	0.866084	-1.15462
Med13	Mediator of RNA polymerase II transcription subunit 13	0.879213	0.688352	0.165308	1.07982	1.07982	0.295168	0.999478	1.1467	1.23374	1.23374
Lad1	Ladinin-1	0.841574	0.67991	0.217778	1.08312	1.08312	0.746087	0.999478	0.339102	1.18103	1.18103
Prex1	Phosphatidylinositol 3,4,5-trisphosphate-dependent Rac exchanger 1 protein	0.944064	0.704767	0.076191	1.08535	1.08535	0.216008	0.999478	1.38273	2.41374	2.41374
Ppp1cb	Serine/threonine-protein phosphatase PP1-beta catalytic subunit;Serine/threonine-protein phosph	0.723239	0.673321	0.389007	1.08644	1.08644	0.905414	0.999478	-0.12394	0.956391	-1.0456
Lamb2	Laminin subunit beta-2	0.289683	0.622392	1.28282	1.08775	1.08775	0.260978	0.999478	-1.24085	0.514489	-1.94368
Hadhb	Trifunctional enzyme subunit beta, mitochondrial;3-ketoacyl-CoA thiolase	0.860335	0.684534	0.191537	1.08822	1.08822	0.977377	0.999478	0.02956	1.01851	1.01851
Gstm1	Glutathione S-transferase Mu 1;Glutathione S-transferase Mu 7	0.580809	0.642019	0.617156	1.08889	1.08889	0.711346	0.999478	-0.38809	0.776673	-1.28754
Etfa	Electron transfer flavoprotein subunit alpha, mitochondrial	0.630726	0.658425	0.53343	1.0893	1.0893	0.816265	0.999478	0.242776	1.31898	1.31898
Eef1g	Elongation factor 1-gamma	0.197494	0.622392	1.65006	1.08989	1.08989	0.058793	0.999478	-2.32811	0.548692	-1.82252
Rab6a	Ras-related protein Rab-6B;Ras-related protein Rab-6A;Ras-related protein Rab-39A	0.769728	0.673321	0.320337	1.09222	1.09222	0.155959	0.999478	1.62186	1.48887	1.48887
Atp5d	ATP synthase subunit delta, mitochondrial	0.583654	0.642019	0.612251	1.09235	1.09235	0.752723	0.999478	-0.32985	0.92794	-1.07766
Cryzl2		0.728569	0.673321	0.381022	1.09953	1.09953	0.965732	0.999478	-0.04479	0.973589	-1.02713
Cttn	Src substrate cortactin	0.904332	0.694622	0.130635	1.10092	1.10092	0.725649	0.999478	-0.3678	0.812676	-1.2305
Cpne1	Copine-1	0.849677	0.682222	0.206422	1.10304	1.10304	0.728331	0.999478	-0.36401	0.878812	-1.1379
Mat2a	S-adenosylmethionine synthase;S-adenosylmethionine synthase isoform type-2	0.88589	0.689971	0.156068	1.10327	1.10327	0.355918	0.999478	-1	0.716323	-1.39602
Xylb	Xylulose kinase	0.859819	0.684534	0.192257	1.10474	1.10474	0.875637	0.999478	-0.16331	0.890627	-1.1228
Prdx4	Peroxiredoxin-4	0.747603	0.673321	0.352752	1.10484	1.10484	0.709791	0.999478	0.390305	1.11863	1.11863
Gm9774	Proteasomal ubiquitin receptor ADRM1	0.752564	0.673321	0.345444	1.10541	1.10541	0.13135	0.999478	-1.74636	0.315519	-3.16938
Cald1		0.76134	0.673321	0.332573	1.10824	1.10824	0.247921	0.999478	-1.27965	0.330537	-3.02538
Cyp4b1	Cytochrome P450 4B1	0.903392	0.694622	0.131928	1.11883	1.11883	0.197539	0.999478	1.44889	2.17857	2.17857
Eci1	Enoyl-CoA delta isomerase 1, mitochondrial	0.742211	0.673321	0.360723	1.12027	1.12027	0.803082	0.999478	-0.26064	0.911445	-1.09716
Col4a1	Collagen alpha-1(IV) chain;Arresten	0.303681	0.622392	1.23827	1.12184	1.12184	0.859259	0.999478	-0.18509	0.939438	-1.06447
Nid1	Nidogen-1	0.745818	0.673321	0.355388	1.12697	1.12697	0.805578	0.999478	0.257254	1.19382	1.19382
Acsm2	Acyl-coenzyme A synthetase ACSM2, mitochondrial	0.830032	0.67991	0.234018	1.12788	1.12788	0.267691	0.999478	1.22155	3.1172	3.1172
Add1	Alpha-adducin	0.826012	0.67991	0.239693	1.13007	1.13007	0.982927	0.999478	0.022307	1.00802	1.00802
Lmo1	Rhombotin-1	0.874316	0.688352	0.172097	1.13011	1.13011	0.650386	0.999478	0.476781	1.24075	1.24075
Pdha1	Pyruvate dehydrogenase E1 component subunit alpha;Pyruvate dehydrogenase E1 component sub	0.945989	0.704767	0.073562	1.13036	1.13036	0.652762	0.999478	0.473249	1.95134	1.95134
Sod2	Superoxide dismutase;Superoxide dismutase [Mn], mitochondrial	0.378262	0.624312	1.0313	1.13113	1.13113	0.394009	0.999478	0.91803	1.22764	1.22764
Fermt2	Fermitin family homolog 2	0.80162	0.677598	0.274353	1.1326	1.1326	0.138205	0.999478	-1.70955	0.675952	-1.47939
Uqcrc1	Cytochrome b-c1 complex subunit 1, mitochondrial	0.828522	0.67991	0.236148	1.13731	1.13731	0.837975	0.999478	0.213543	1.13124	1.13124
Pomgnt1	Protein O-linked-mannose beta-1,2-N-acetylglucosaminyltransferase 1	0.650605	0.658425	0.501346	1.13766	1.13766	0.190538	0.999478	-1.47547	0.575875	-1.73649
Msra	Mitochondrial peptide methionine sulfoxide reductase	0.863516	0.68459	0.187106	1.13857	1.13857	0.27526	0.999478	-1.2003	0.622931	-1.60532
Selenbp1	Selenium-binding protein 1;Selenium-binding protein 2	0.511954	0.625426	0.741895	1.14231	1.14231	0.862233	0.999478	-0.18113	0.922407	-1.08412
Hint1	Histidine triad nucleotide-binding protein 1	0.698074	0.666274	0.427148	1.14641	1.14641	0.14758	0.999478	-1.66198	0.548875	-1.82191
Psma3	Proteasome subunit alpha type;Proteasome subunit alpha type-3	0.867708	0.686675	0.181274	1.1481	1.1481	0.768204	0.999478	-0.3084	0.819853	-1.21973
Slc25a11	Mitochondrial 2-oxoglutarate/malate carrier protein	0.793378	0.677598	0.286161	1.14853	1.14853	0.259249	0.999478	1.24588	1.67397	1.67397
Lmnb2	Lamin-B2	0.828161	0.67991	0.236658	1.1565	1.1565	0.847628	0.999478	0.200613	1.20889	1.20889
Cox5a	Cytochrome c oxidase subunit 5A, mitochondrial	0.150072	0.622392	1.92383	1.15719	1.15719	0.799413	0.999478	0.265633	1.05993	1.05993
Suclg2	Succinyl-CoA ligase [GDP-forming] subunit beta, mitochondrial;Succinyl-CoA ligase subunit beta	0.72495	0.673321	0.38644	1.15907	1.15907	0.912192	0.999478	0.115007	1.08457	1.08457
P4hb	Protein disulfide-isomerase	0.637969	0.658425	0.521665	1.16185	1.16185	0.996179	0.999478	-0.00499	0.995503	-1.00452
Sucla2	Succinyl-CoA ligase subunit beta;Succinyl-CoA ligase [ADP-forming] subunit beta, mitochondrial	0.83496	0.67991	0.227074	1.16445	1.16445	0.655608	0.999478	0.469027	1.31605	1.31605
Atp5h	ATP synthase subunit d, mitochondrial	0.829445	0.67991	0.234847	1.1647	1.1647	0.20169	0.999478	-1.43354	0.449269	-2.22584
Dsp	Desmoplakin	0.836731	0.67991	0.224583	1.17057	1.17057	0.827457	0.999478	0.227678	1.0789	1.0789
Serpina1a	Serine protease inhibitor A3K	0.808498	0.677598	0.264539	1.18377	1.18377	0.467785	0.999478	0.774978	1.74682	1.74682

Akr7a2	Aflatoxin B1 aldehyde reductase member 2	0.769066	0.673321	0.321301	1.18599	1.18599	0.919067	0.999478	-0.10596	0.960999	-1.04058
Atp5b	ATP synthase subunit beta, mitochondrial	0.532527	0.625426	0.703319	1.18627	1.18627	0.776297	0.999478	0.297252	1.12156	1.12156
Gnb2l1	Guanine nucleotide-binding protein subunit beta-2-like 1;Guanine nucleotide-binding protein subu	0.804811	0.677598	0.269796	1.18944	1.18944	0.332273	0.999478	1.05446	2.20482	2.20482
Akr1c21	Aldo-keto reductase family 1 member C21	0.536699	0.625426	0.695642	1.18986	1.18986	0.257952	0.999478	1.24968	1.52129	1.52129
Dsc1	Desmocollin-1	0.690796	0.665708	0.438322	1.19009	1.19009	0.645239	0.999478	0.484455	1.16112	1.16112
Got1	Aspartate aminotransferase, cytoplasmic	0.496866	0.624776	0.770996	1.19273	1.19273	0.339818	0.999478	-1.03675	0.733884	-1.36261
Sorbs2	Sorbin and SH3 domain-containing protein 2	0.881217	0.688695	0.162533	1.19561	1.19561	0.596443	0.999478	-0.55892	0.70144	-1.42564
Hagh	Hydroxyacylglutathione hydrolase, mitochondrial; hydrolysis of S-lactoyl-glutathione to reduced glu	0.289468	0.622392	1.28353	1.19582	1.19582	0.935898	0.999478	-0.08386	0.972473	-1.02831
Alad	Delta-aminolevulinic acid dehydratase	0.423756	0.624312	0.923732	1.20865	1.20865	0.303365	0.999478	-1.12552	0.678436	-1.47398
Cat	Catalase	0.676882	0.660372	0.459882	1.21563	1.21563	0.251524	0.999478	1.26876	1.46721	1.46721
Atp1a1	Sodium/potassium-transporting ATPase subunit alpha-1	0.665294	0.658425	0.478041	1.21825	1.21825	0.785421	0.999478	0.284734	1.13007	1.13007
Acadm	Medium-chain specific acyl-CoA dehydrogenase, mitochondrial	0.586558	0.643605	0.607259	1.22363	1.22363	0.461732	0.999478	0.786072	1.2371	1.2371
Psma7	Proteasome subunit alpha type;Proteasome subunit alpha type-7;Proteasome subunit alpha type-7	0.904587	0.694622	0.130284	1.22429	1.22429	0.349308	0.999478	-1.01492	0.36219	-2.76099
Pecr	Peroxisomal trans-2-enoyl-CoA reductase	0.738038	0.673321	0.366912	1.22508	1.22508	0.263156	0.999478	1.23454	1.83484	1.83484
Aldh2	Aldehyde dehydrogenase, mitochondrial	0.448221	0.624776	0.870207	1.22835	1.22835	0.979613	0.999478	0.026637	1.00992	1.00992
Npepps	Puromycin-sensitive aminopeptidase	0.803757	0.677598	0.271301	1.22844	1.22844	0.946044	0.999478	0.070557	1.04452	1.04452
Vdac1	Voltage-dependent anion-selective channel protein 1	0.076463	0.456159	2.6581	1.23137	1.23137	0.213139	0.999478	1.39266	1.43155	1.43155
Dsg1b	Desmoglein-1-alpha;Desmoglein-1-beta;Desmoglein-1-gamma	0.580369	0.642019	0.617917	1.24177	1.24177	0.847332	0.999478	0.201009	1.05638	1.05638
Got2	Aspartate aminotransferase, mitochondrial	0.629545	0.658425	0.535357	1.245	1.245	0.78142	0.999478	0.290216	1.20092	1.20092
Cpm	Carboxypeptidase M	0.35189	0.62345	1.09945	1.25026	1.25026	0.023353	0.999478	-3.02146	0.433259	-2.30809
Alb	Serum albumin	0.760609	0.673321	0.333642	1.25296	1.25296	0.422289	0.999478	0.861034	1.90712	1.90712
Napb	Beta-soluble NSF attachment protein	0.836668	0.67991	0.224672	1.25346	1.25346	0.434988	0.999478	0.836362	1.87849	1.87849
mt-Atp8	ATP synthase protein 8	0.657456	0.658425	0.490436	1.2543	1.2543	0.141596	0.999478	-1.69199	0.650599	-1.53705
Anxa4	Annexin;Annexin A4	0.44626	0.624776	0.8744	1.25474	1.25474	0.635667	0.999478	-0.49882	0.711859	-1.40477
Fh	Fumarate hydratase, mitochondrial	0.776614	0.674789	0.310339	1.25868	1.25868	0.652613	0.999478	0.473471	1.40588	1.40588
Lgalsl	Galectin-related protein	0.495025	0.624776	0.774597	1.25991	1.25991	0.390714	0.999478	0.924872	1.3752	1.3752
Cst14	Cystatin-14	0.495025	0.624776	0.774597	1.25992	1.25992	0.18865	0.999478	-1.48279	0.546234	-1.83072
Gm10367		0.495025	0.624776	0.774597	1.25992	1.25992	0.139271	0.999478	-1.70399	0.671767	-1.48861
Actn2	Alpha-actinin-2	0.495025	0.624776	0.774597	1.25993	1.25993	0.10133	0.999478	1.93365	3.84099	3.84099
Mug1	Murinoglobulin-1	0.495025	0.624776	0.774597	1.25993	1.25993	0.326471	0.999478	1.0683	1.69698	1.69698
Hspb1		0.495025	0.624776	0.774597	1.26203	1.26203	0.159032	0.999478	1.60767	2.11568	2.11568
Acat1	Acetyl-CoA acetyltransferase, mitochondrial	0.38595	0.624312	1.01229	1.26233	1.26233	0.542004	0.999478	0.646295	1.55071	1.55071
Atp6v1h	V-type proton ATPase subunit H	0.44397	0.624776	0.879317	1.26878	1.26878	0.6001	0.999478	-0.55323	0.650782	-1.53661
Rps19	40S ribosomal protein S19	0.324904	0.62345	1.1746	1.30464	1.30464	0.664602	0.999478	-0.45575	0.838463	-1.19266
Rab14	Ras-related protein Rab-14	0.30096	0.622392	1.24676	1.30552	1.30552	0.971526	0.999478	0.037208	1.01617	1.01617
Cox7a2	Cytochrome c oxidase subunit 7A2, mitochondrial	0.097476	0.557008	2.38161	1.30852	1.30852	0.494169	0.999478	-0.72776	0.878025	-1.13892
Cat	Catalase	0.647912	0.658425	0.505655	1.31169	1.31169	0.973014	0.999478	-0.03526	0.984592	-1.01565
Prdx2	Peroxiredoxin-2	0.635899	0.658425	0.525018	1.31451	1.31451	0.607774	0.999478	0.541337	1.26253	1.26253
Acads	Short-chain specific acyl-CoA dehydrogenase, mitochondrial	0.374475	0.624312	1.04079	1.32316	1.32316	0.318812	0.999478	1.08688	1.39234	1.39234
Fah	Fumarylacetoacetase	0.469179	0.624776	0.826381	1.32515	1.32515	0.469831	0.999478	0.771252	1.22268	1.22268
Jup	Junction plakoglobin	0.717317	0.673321	0.397915	1.32668	1.32668	0.713396	0.999478	-0.38517	0.887879	-1.12628
Zyx	Zyxin	0.604924	0.650323	0.576104	1.33761	1.33761	0.145762	0.999478	-1.67097	0.619966	-1.61299
Acadl	Long-chain specific acyl-CoA dehydrogenase, mitochondrial	0.183956	0.622392	1.71981	1.34145	1.34145	0.587573	0.999478	0.572822	1.4465	1.4465
Pgk1	Phosphoglycerate kinase 1	0.533631	0.625426	0.701282	1.36249	1.36249	0.995224	0.999478	0.006239	1.00198	1.00198
Anxa2	Annexin;Annexin A2	0.293929	0.622392	1.26908	1.36364	1.36364	0.604634	0.999478	-0.54619	0.921017	-1.08576
Eif6	Eukaryotic translation initiation factor 6	0.748997	0.673321	0.350696	1.36527	1.36527	0.355918	0.999478	-1	0.610516	-1.63796
Gstt1	Glutathione S-transferase theta-1	0.744605	0.673321	0.35718	1.36857	1.36857	0.771808	0.999478	-0.30343	0.83922	-1.19158
Etfdh	Electron transfer flavoprotein-ubiquinone oxidoreductase, mitochondrial	0.681821	0.662255	0.452198	1.36896	1.36896	0.938446	0.999478	0.080515	1.04629	1.04629
Col4a2	Collagen alpha-2(IV) chain;Canstatin	0.40725	0.624312	0.961455	1.37601	1.37601	0.818753	0.999478	0.239415	1.11263	1.11263
Gss	Glutathione synthetase	0.347156	0.62345	1.11221	1.37931	1.37931	0.605734	0.999478	-0.54449	0.784531	-1.27465
Acad8	Isobutyryl-CoA dehydrogenase, mitochondrial	0.653818	0.658425	0.496221	1.38147	1.38147	0.76004	0.999478	-0.31969	0.852065	-1.17362

Lyz1	Lysozyme;Lysozyme C-1	0.660934	0.658425	0.484925	1.38685	1.38685	0.512243	0.999478	0.696395	2.12543	2.12543
Dlst	Dihydrolipoyllysine-residue succinyltransferase component of 2-oxoglutarate dehydrogenase comp	0.692941	0.665708	0.435022	1.3916	1.3916	0.225349	0.999478	1.35122	2.50668	2.50668
Ptp4a2		0.735556	0.673321	0.370601	1.41055	1.41055	0.980314	0.999478	-0.02572	0.981664	-1.01868
Lrp2	Low-density lipoprotein receptor-related protein 2	0.664746	0.658425	0.478906	1.41105	1.41105	0.97359	0.999478	0.034509	1.01947	1.01947
Aldh9a1	4-trimethylaminobutyraldehyde dehydrogenase	0.341947	0.62345	1.12645	1.42191	1.42191	0.541881	0.999478	-0.6465	0.727178	-1.37518
Hnrnpm	Heterogeneous nuclear ribonucleoprotein M	0.349476	0.62345	1.10593	1.42803	1.42803	0.403991	0.999478	-0.89757	0.576336	-1.7351
Igh		0.495025	0.624776	0.774597	1.43708	1.43708	0.769947	0.999478	-0.306	0.906036	-1.10371
Strbp	Spermatid perinuclear RNA-binding protein	0.107117	0.572676	2.27816	1.44934	1.44934	0.721703	0.999478	-0.37338	0.914383	-1.09363
Sec31a	Protein transport protein Sec31A	0.641426	0.658425	0.516081	1.47174	1.47174	0.493346	0.999478	-0.72921	0.680985	-1.46846
Gm5409		0.018348	0.351001	4.68798	1.47572	1.47572	0.863576	0.999478	0.179336	1.02612	1.02612
Ak4	Adenylate kinase 4, mitochondrial	0.600602	0.647709	0.583373	1.47866	1.47866	0.932848	0.999478	-0.08786	0.957405	-1.04449
Hibadh	3-hydroxyisobutyrate dehydrogenase;3-hydroxyisobutyrate dehydrogenase, mitochondrial	0.475961	0.624776	0.812562	1.48173	1.48173	0.677757	0.999478	-0.43648	0.75069	-1.33211
Arg1	Arginase-1	0.13463	0.604462	2.03549	1.48205	1.48205	0.909807	0.999478	-0.11815	0.961488	-1.04005
Ggt1	Rab GDP dissociation inhibitor beta	0.646868	0.658425	0.507328	1.49076	1.49076	0.680952	0.999478	0.431829	1.29291	1.29291
Psmb2	Proteasome subunit beta type;Proteasome subunit beta type-2	0.519143	0.625426	0.728275	1.52647	1.52647	0.441805	0.999478	-0.82334	0.72011	-1.38868
Gm20441	D-dopachrome decarboxylase	0.486415	0.624776	0.791588	1.53778	1.53778	0.5812	0.999478	-0.58289	0.773859	-1.29223
Gulo	L-gulonolactone oxidase	0.495025	0.624776	0.774597	1.57209	1.57209	0.424086	0.999478	0.85751	1.44427	1.44427
Aldh4a1	Delta-1-pyrroline-5-carboxylate dehydrogenase, mitochondrial	0.210639	0.622392	1.58732	1.57305	1.57305	0.765093	0.999478	0.312698	1.19047	1.19047
Ccdc141		0.330241	0.62345	1.15925	1.57585	1.57585	0.490029	0.999478	-0.73505	0.811972	-1.23157
Amacr	Alpha-methylacyl-CoA racemase	0.509034	0.625426	0.747471	1.57742	1.57742	0.462914	0.999478	-0.7839	0.706493	-1.41544
Cct2	T-complex protein 1 subunit beta	0.498737	0.624776	0.767348	1.58249	1.58249	0.142076	0.999478	-1.68954	0.546518	-1.82977
Apoa1	Apolipoprotein A-I;Proapolipoprotein A-I;Truncated apolipoprotein A-I	0.470386	0.624776	0.823909	1.59134	1.59134	0.342702	0.999478	1.03006	2.09971	2.09971
Tagln	Transgelin	0.704565	0.669565	0.417237	1.59805	1.59805	0.91623	0.999478	-0.10969	0.900662	-1.11029
Krt76	Keratin, type II cytoskeletal 2 oral	0.257786	0.622392	1.39341	1.6036	1.6036	0.347261	0.999478	1.01959	1.38244	1.38244
Krt77		0.644453	0.658425	0.511207	1.60465	1.60465	0.279213	0.999478	1.1894	1.99253	1.99253
Calb1	Calbindin;Calretinin	0.692513	0.665708	0.43568	1.61016	1.61016	0.355918	0.999478	-1	0.539468	-1.85368
Ldha	L-lactate dehydrogenase;L-lactate dehydrogenase A chain;L-lactate dehydrogenase C chain	0.118143	0.572676	2.17281	1.6517	1.6517	0.500664	0.999478	-0.7164	0.788383	-1.26842
Lamp1	Lysosome-associated membrane glycoprotein 1	0.468395	0.624776	0.827989	1.68285	1.68285	0.492578	0.999478	-0.73056	0.702422	-1.42364
Myl9	Myosin regulatory light polypeptide 9	0.001041	0.0458118	12.7488	1.69127	1.69127	0.269108	0.999478	-1.21753	0.535371	-1.86786
Chdh	Choline dehydrogenase, mitochondrial	0.645408	0.658425	0.509672	1.70627	1.70627	0.635042	0.999478	0.499756	1.60793	1.60793
Ndufs7	NADH dehydrogenase [ubiquinone] iron-sulfur protein 7, mitochondrial	0.47655	0.624776	0.811369	1.70786	1.70786	0.899302	0.999478	0.131996	1.07726	1.07726
Anxa7	Annexin;Annexin A7	0.178685	0.622392	1.74856	1.7116	1.7116	0.296899	0.999478	-1.14219	0.646963	-1.54568
Fgg	Fibrinogen gamma chain	0.495025	0.624776	0.774597	1.71579	1.71579	0.758045	0.999478	0.322458	1.19906	1.19906
Ndufs4	NADH dehydrogenase [ubiquinone] iron-sulfur protein 4, mitochondrial	0.230867	0.622392	1.49882	1.72327	1.72327	0.767438	0.999478	-0.30946	0.889614	-1.12408
Cndp1	Beta-Ala-His dipeptidase	0.571454	0.63762	0.633416	1.72926	1.72926	0.698574	0.999478	-0.40636	0.766248	-1.30506
Mical3	Protein-methionine sulfoxide oxidase MICAL3	0.237067	0.622392	1.4734	1.76078	1.76078	0.39979	0.999478	-0.90613	0.629139	-1.58947
Acox1	Acyl-coenzyme A oxidase;Peroxisomal acyl-coenzyme A oxidase 1	0.189309	0.622392	1.69155	1.78518	1.78518	0.308678	0.999478	1.11205	1.46672	1.46672
Clu	Clusterin;Clusterin;Clusterin beta chain;Clusterin alpha chain	0.302249	0.622392	1.24273	1.82685	1.82685	0.998253	0.999478	0.002282	1.00207	1.00207
Ech1	Delta(3,5)-Delta(2,4)-dienoyl-CoA isomerase, mitochondrial	0.465202	0.624776	0.834563	1.83417	1.83417	0.574928	0.999478	-0.59285	0.705231	-1.41797
Slc27a2	Very long-chain acyl-CoA synthetase	0.427619	0.624312	0.915098	1.85569	1.85569	0.982941	0.999478	-0.02229	0.984327	-1.01592
Cul3	Cullin-3	0.058306	0.453261	2.98622	1.86321	1.86321	0.273561	0.999478	-1.20502	0.694941	-1.43897
Prdx1	Peroxiredoxin-1	0.35516	0.624312	1.09073	1.87063	1.87063	0.806819	0.999478	0.255569	1.13983	1.13983
Aifm1	Apoptosis-inducing factor 1, mitochondrial	0.203058	0.622392	1.62295	1.88885	1.88885	0.663156	0.999478	0.457874	1.25673	1.25673
Hadh	Hydroxyacyl-coenzyme A dehydrogenase, mitochondrial	0.257122	0.622392	1.39586	1.8964	1.8964	0.15809	0.999478	-1.61199	0.618803	-1.61602
Gpx3	Glutathione peroxidase 3	0.646601	0.658425	0.507757	1.91762	1.91762	0.690045	0.999478	0.41865	1.29229	1.29229
Ldhd	Probable D-lactate dehydrogenase, mitochondrial	0.413084	0.624312	0.947962	1.94758	1.94758	0.813765	0.999478	0.246157	1.14004	1.14004
Cisd1	CDGSH iron-sulfur domain-containing protein 1	0.423941	0.624312	0.923316	1.96883	1.96883	0.546986	0.999478	0.638081	1.48887	1.48887
Psma1	Proteasome subunit alpha type;Proteasome subunit alpha type-1	0.260942	0.622392	1.38184	1.99923	1.99923	0.045091	0.999478	-2.52322	0.500146	-1.99942
Ivd	Isovaleryl-CoA dehydrogenase, mitochondrial	0.279551	0.622392	1.31649	2.02406	2.02406	0.760522	0.999478	0.319025	1.19988	1.19988
Gm20425	Serotransferrin	0.169597	0.622392	1.80049	2.03407	2.03407	0.518028	0.999478	0.686512	1.82642	1.82642
Acy3	N-acyl-aromatic-L-amino acid amidohydrolase (carboxylate-forming)	0.605983	0.650323	0.574328	2.06071	2.06071	0.564427	0.999478	0.609689	1.68868	1.68868

Ide	Insulin-degrading enzyme	0.287081	0.622392	1.29135	2.07736	2.07736	0.450125	0.999478	0.807635	1.93547	1.93547
A2m	Alpha-2-macroglobulin;Alpha-2-macroglobulin 165 kDa subunit;Alpha-2-macroglobulin 35 kDa subu	0.495025	0.624776	0.774597	2.13618	2.13618	0.170286	0.999478	1.55782	3.79144	3.79144
Dlat	Dihydrolipoyllysine-residue acetyltransferase component of pyruvate dehydrogenase complex, mit	0.26	0.622392	1.38528	2.13862	2.13862	0.915985	0.999478	-0.11002	0.939442	-1.06446
Psmb3	Proteasome subunit beta type;Proteasome subunit beta type-3	0.495025	0.624776	0.774597	2.15117	2.15117	0.528604	0.999478	-0.66863	0.6695	-1.49365
Tuba4a	Tubulin alpha-4A chain	0.204963	0.622392	1.61386	2.17669	2.17669	0.936284	0.999478	0.083351	1.04497	1.04497
Tbx20	T-box transcription factor TBX20	0.112862	0.572676	2.22172	2.224	2.224	0.727793	0.999478	-0.36477	0.917136	-1.09035
Psmb4	Proteasome subunit beta type-4	0.362988	0.624312	1.07018	2.25043	2.25043	0.752691	0.999478	0.329897	1.37769	1.37769
Dnajb4	DnaJ homolog subfamily B member 4	0.546119	0.627395	0.678477	2.26341	2.26341	0.811862	0.999478	0.248733	1.19286	1.19286
Me1	Malic enzyme;NADP-dependent malic enzyme	0.304919	0.622392	1.23444	2.27495	2.27495	0.604475	0.999478	-0.54644	0.748216	-1.33651
Psmb5	Proteasome subunit beta type-5	0.345495	0.62345	1.11672	2.27906	2.27906	0.860155	0.999478	-0.18389	0.892609	-1.12031
Sardh	Sarcosine dehydrogenase, mitochondrial	0.303157	0.622392	1.2399	2.28264	2.28264	0.718201	0.999478	0.378343	1.33026	1.33026
Pkp1	Plakophilin-1	0.279586	0.622392	1.31638	2.28283	2.28283	0.078901	0.999478	-2.11432	0.535933	-1.8659
Atp1b1	Sodium/potassium-transporting ATPase subunit beta-1	0.195496	0.622392	1.66001	2.29115	2.29115	0.892239	0.999478	0.141325	1.05865	1.05865
Try4		0.233283	0.622392	1.48883	2.37277	2.37277	0.897512	0.999478	0.134359	1.09223	1.09223
Usp34	Ubiquitin carboxyl-terminal hydrolase;Ubiquitin carboxyl-terminal hydrolase 34	0.220532	0.622392	1.54291	2.37833	2.37833	0.860762	0.999478	-0.18308	0.908831	-1.10031
Echs1	Enoyl-CoA hydratase, mitochondrial	0.299457	0.622392	1.25149	2.38736	2.38736	0.947007	0.999478	-0.06929	0.953627	-1.04863
Ces1c	Carboxylesterase 1C;Carboxylic ester hydrolase	0.525549	0.625426	0.716268	2.48243	2.48243	0.862847	0.999478	-0.18031	0.824104	-1.21344
Chd3		0.283891	0.622392	1.30192	2.52049	2.52049	0.860187	0.999478	-0.18385	0.89502	-1.11729
Dtd1	D-tyrosyl-tRNA(Tyr) deacylase 1	0.401185	0.624312	0.975672	2.53606	2.53606	0.437002	0.999478	-0.8325	0.490624	-2.03822
Ces1f	Carboxylic ester hydrolase	0.478923	0.624776	0.80658	2.61788	2.61788	0.86225	0.999478	-0.1811	0.840776	-1.18938
Capns2	Calpain small subunit 2	0.266015	0.622392	1.36355	2.67876	2.67876	0.164019	0.999478	-1.58518	0.477584	-2.09387
Ndufa4	Cytochrome c oxidase subunit NDUFA4	0.235266	0.622392	1.48071	2.69379	2.69379	0.285044	0.999478	-1.17356	0.498491	-2.00605
Lypla1	Acyl-protein thioesterase 1	0.005716	0.179581	7.11407	2.70705	2.70705	0.771079	0.999478	-0.30444	0.874668	-1.14329
Psma6	Proteasome subunit alpha type-6	0.024197	0.354885	4.22836	2.76906	2.76906	0.384037	0.999478	-0.93887	0.698804	-1.43102
Col1a2	Collagen alpha-2(I) chain	0.30553	0.622392	1.23255	2.80882	2.80882	0.638651	0.999478	0.494325	1.11774	1.11774
Clcf1	Cardiotrophin-like cytokine factor 1	0.759396	0.673321	0.335418	2.83463	2.83463	0.880054	0.999478	-0.15745	0.724676	-1.37993
Snrpd2	Small nuclear ribonucleoprotein Sm D2	0.11756	0.572676	2.17809	3.06656	3.06656	0.306437	0.999478	-1.11771	0.333235	-3.00089
trypsinogen		0.236289	0.622392	1.47655	3.08767	3.08767	0.822417	0.999478	0.234469	1.09338	1.09338
Blmh	Bleomycin hydrolase	0.155522	0.622392	1.8876	3.1339	3.1339	0.536914	0.999478	-0.65474	0.757591	-1.31997
Serpinb8	Serpin B8	0.023958	0.354885	4.2442	3.17657	3.17657	0.294269	0.999478	1.14905	1.50953	1.50953
Mup20	Major urinary protein 20	0.321299	0.62345	1.18511	3.21376	3.21376	0.312793	0.999478	-1.10175	0.495445	-2.01839
Ehhadh	Peroxisomal bifunctional enzyme;Enoyl-CoA hydratase/3,2-trans-enoyl-CoA isomerase;3-hydroxyac	0.425362	0.624312	0.920133	3.2314	3.2314	0.573019	0.999478	0.5959	1.82455	1.82455
Sprr2k	Small proline-rich protein 2K;Small proline-rich protein 2G;Small proline-rich protein 2D;Small proli	0.495025	0.624776	0.774597	3.28678	3.28678	0.750976	0.999478	0.332285	1.42631	1.42631
Cndp2	Cytosolic non-specific dipeptidase	0.332324	0.62345	1.15333	3.54481	3.54481	0.961383	0.999478	0.050474	1.06471	1.06471
S100a14	Protein S100-A14	0.011107	0.271507	5.62579	3.6455	3.6455	0.38858	0.999478	-0.92932	0.684189	-1.46158
Tgm1	Protein-glutamine gamma-glutamyltransferase K	0.004951	0.167583	7.4787	3.66777	3.66777	0.050713	0.999478	-2.4365	0.406278	-2.46137
Ighg2b	Ig gamma-2B chain C region	0.335971	0.62345	1.14305	3.7256	3.7256	0.756602	0.999478	0.324462	1.49817	1.49817
C3	Complement C3;Complement C3 beta chain;C3-beta-c;Complement C3 alpha chain;C3a anaphylato	0.204219	0.622392	1.6174	3.90424	3.90424	0.496529	0.999478	-0.72362	0.53406	-1.87245
Psma5	Proteasome subunit alpha type;Proteasome subunit alpha type-5	0.115931	0.572676	2.19298	4.25974	4.25974	0.12269	0.999478	-1.79564	0.46408	-2.1548
Tgm3	Protein-glutamine gamma-glutamyltransferase E;Protein-glutamine gamma-glutamyltransferase E	0.024152	0.354885	4.23134	5.01022	5.01022	0.979361	0.999478	0.026966	1.02033	1.02033
Pdzk1	Na(+)/H(+) exchange regulatory cofactor NHE-RF3	0.300687	0.622392	1.24762	5.1399	5.1399	0.810839	0.999478	-0.25012	0.747282	-1.33818
Gsdma	Gasdermin-A	0.076718	0.456159	2.6542	5.97242	5.97242	0.23913	0.999478	-1.3068	0.728933	-1.37187
Mup14	Major urinary protein 6;Major urinary protein 17;Major urinary proteins 11 and 8;Major urinary pr	0.280693	0.622392	1.31264	6.74611	6.74611	0.231151	0.999478	-1.33223	0.284099	-3.5199
Igkc	Ig kappa chain C region;Ig kappa chain V-II region 26-10	0.026532	0.354929	4.0834	6.76443	6.76443	0.421552	0.999478	-0.86248	0.582378	-1.7171
Plec	Plakophilin-3	0.060748	0.453261	2.93514	6.82042	6.82042	0.255932	0.999478	-1.25563	0.334578	-2.98884
Ftl1	Ferritin;Ferritin light chain 1;Ferritin light chain 2	0.042264	0.450818	3.40634	6.83394	6.83394	0.042828	0.999478	-2.56141	0.194763	-5.13444
Hal	Histidine ammonia-lyase	0.013613	0.299477	5.22837	8.5428	8.5428	0.204276	0.999478	-1.42413	0.282931	-3.53443
Apoe	Apolipoprotein E	0.043151	0.450818	3.37814	9.32165	9.32165	0.960697	0.999478	0.051371	1.03313	1.03313
Fabp5	Fatty acid-binding protein, epidermal	0.273569	0.622392	1.33698	10.8216	10.8216	0.810833	0.999478	-0.25013	0.613256	-1.63064
Nccrp1	F-box only protein 50; promote cell proliferation	0.000586	0.0322262	15.4779	12.1769	12.1769	0.017692	0.999478	-3.23977	0.271152	-3.68797
Fgf22	Fibroblast growth factor	0.124695	0.584618	2.11566	13.0662	13.0662	0.414346	0.999478	0.876745	2.06475	2.06475

Ighm	Ig mu chain C region	0.100991	0.569695	2.3425	13.0776	13.0776	0.518577	0.999478	-0.68558	0.539332	-1.85415
Prss1		0.544389	0.627045	0.68161	13.3349	13.3349	0.442176	0.999478	0.822632	14.7383	14.7383
Umod	Uromodulin;Uromodulin, secreted form	0.220073	0.622392	1.54492	18.7124	18.7124	0.321506	0.999478	-1.0803	0.23007	-4.3465

STAB2. Targeted Proteomics of Kidney Tubules (N=6 per group)

Gene ID	Protein Name	YWT (pmol/100µg p)		OWT		OTG		Age Effectǂ	Transgenic Effectǂ
		Mean	SEM	Mean	SEM	Mean	SEM		
	**Glycolysis**								
aldoa	aldolase A, fructose-bisphosphate	1.612	0.096	1.569	0.072	1.667	0.080		
aldob	aldolase B, fructose-bisphosphate	9.365	0.794	9.847	0.805	8.734	0.833		
eno1	enolase 1, alpha non-neuron	4.604	0.333	5.191	0.063	5.407	0.374	0.146	
eno3	enolase 3, beta muscle	0.007	0.001	0.009	0.002	0.007	0.001	0.206	
gapdh	glyceraldehyde-3-phosphate dehydrogenase	13.127	1.283	13.535	0.629	13.522	1.096		
gpi	glucose phosphate isomerase 1	0.489	0.035	0.546	0.019	0.538	0.018		
hk1	hexokinase 1	0.614	0.043	0.599	0.066	0.749	0.100		0.182
ldha	lactate dehydrogenase A	5.533	0.643	5.258	0.482	5.051	0.425		
ldhb	lactate dehydrogenase B	3.834	0.393	4.549	0.182	4.646	0.351	0.199	
mdh1	malate dehydrogenase 1, NAD (soluble)	7.409	0.547	7.347	0.373	7.048	0.390		
pc	Pyruvate carboxylase	3.107	0.583	3.529	0.316	3.341	0.524	0.156	
pfkfb2	6-Phosphofructo-2-Kinase/Fructose-2,6-Biphosph	0.005	0.001	0.006	0.000	0.007	0.000	0.366	0.061
pfkl	phosphofructokinase, liver, B-type	0.183	0.015	0.206	0.024	0.226	0.021		0.159
pfkm	phosphofructokinase, muscle	0.086	0.016	0.112	0.008	0.114	0.010	0.371	
pgam2	phosphoglycerate mutase 2	0.593	0.039	0.595	0.045	0.564	0.094		-0.105
pgk1	phosphoglycerate kinase 1	1.665	0.140	1.638	0.033	1.655	0.108		
pkm2	pyruvate kinase, muscle	1.879	0.078	1.873	0.149	2.288	0.247		0.178
pygb	brain glycogen phosphorylase	0.095	0.003	0.106	0.011	0.124	0.011		0.21
pygm	muscle glycogen phosphorylase	0.021	0.001	0.023	0.002	0.024	0.001		
slc2a4	solute carrier family 2 (facilitated glucose transpo	0.187	0.023	0.163	0.032	0.230	0.054		0.195
Taldo1	transaldolase 1	0.176	0.012	0.181	0.005	0.170	0.006		
tkt	transketolase	2.154	0.110	2.155	0.140	2.061	0.070		
tpi	triosephosphate isomerase 1	1.990	0.199	2.010	0.125	1.940	0.129		
	**Krebs Cycle**								
aco2	aconitase 2, mitochondrial	4.743	0.177	4.325	0.220	4.466	0.266	-0.081	
cs	citrate synthase	4.197	0.250	4.077	0.156	3.924	0.192		-0.053
dlat	dihydrolipoamide S-acetyltransferase (E2 compor	1.929	0.404	3.142	0.146	3.191	0.154	0.658	
dld	dihydrolipoamide dehydrogenase	1.153	0.060	1.205	0.076	1.135	0.074		
dlst	dihydrolipoamide S-succinyltransferase (E2 comp	3.431	0.242	3.790	0.230	3.569	0.234	0.071	
fh1	fumarate hydratase 1	1.120	0.081	1.129	0.067	1.075	0.061		

idh1	isocitrate dehydrogenase 1 (NADP+), soluble	3.532	0.220	4.066	0.199	3.784	0.278	0.104	
idh2	isocitrate dehydrogenase 2 (NADP+), mitochondri	5.308	0.322	5.915	0.316	5.835	0.363	0.103	
idh3a	isocitrate dehydrogenase 3 (NAD+) alpha	0.987	0.049	1.083	0.048	1.070	0.069	0.084	
idh3b	isocitrate dehydrogenase 3 (NAD+) beta	0.456	0.073	0.786	0.025	0.758	0.043	0.605	
idh3g	isocitrate dehydrogenase 3 (NAD+), gamma	0.997	0.042	1.081	0.070	1.110	0.074	0.075	0.024
mdh1	malate dehydrogenase 1, NAD (soluble)	7.331	0.516	7.370	0.393	6.939	0.374		-0.055
mdh2	malate dehydrogenase 2, NAD (mitochondrial)	5.422	0.334	5.732	0.241	5.539	0.278		
ogdh	oxoglutarate (alpha-ketoglutarate) dehydrogenas	2.276	0.110	2.174	0.103	2.173	0.135		
pdha1	pyruvate dehydrogenase E1 alpha 1	2.204	0.119	2.121	0.083	2.041	0.097		-0.058
pdhb	pyruvate dehydrogenase (lipoamide) beta	1.004	0.054	0.999	0.025	0.999	0.048		
pdk1	pyruvate dehydrogenase kinase, isoenzyme 1	0.037	0.005	0.050	0.002	0.051	0.005	0.339	
pdk2	pyruvate dehydrogenase kinase, isoenzyme2	0.112	0.008	0.124	0.002	0.124	0.007	0.113	
pdk4	pyruvate dehydrogenase kinase, isoenzyme 4	0.010	0.003	0.015	0.002	0.015	0.002	0.439	
sdha	succinate dehydrogenase complex, subunit A, flav	1.839	0.095	1.828	0.077	1.726	0.094		-0.062
sdhb	succinate dehydrogenase complex, subunit B, iro	1.539	0.101	1.660	0.094	1.549	0.099	0.042	
sdhc	succinate dehydrogenase complex, subunit C, inte	0.203	0.013	0.238	0.014	0.196	0.004	0.16	-0.186
sucla2	succinate-Coenzyme A ligase, ADP-forming, beta	0.717	0.090	1.084	0.038	1.071	0.063	0.414	
suclg1	succinate-CoA ligase, GDP-forming, alpha subunit	1.009	0.074	1.199	0.071	1.093	0.084	0.128	
	**Respiratory Complex and Other Mitochondrial Proteins**								
Atp5a1	ATP synthase, H+ transporting, mitochondrial F1	7.758	0.992	11.800	0.421	11.164	0.572	0.462	-0.059
Atp5b	ATP synthase, H+ transporting mitochondrial F1 c	9.251	1.437	13.405	0.626	12.683	0.781	0.407	
cd36	CD36 antigen	0.392	0.074	0.300	0.021	0.299	0.040	-0.18	-0.034
ckm	creatine kinase, muscle	0.005	0.001	0.007	0.002	0.004	0.001	0.216	-0.506
ckmt1/2	creatine kinase, mitochondrial 2	1.044	0.049	0.926	0.041	0.977	0.101		
Coq6	coenzyme Q6 homolog (yeast)	0.040	0.002	0.047	0.002	0.044	0.003	0.121	
etfa	electron transferring flavoprotein, alpha polypept	3.230	0.195	3.758	0.270	3.355	0.161	0.147	-0.106
etfb	electron transferring flavoprotein, beta polypepti	3.222	0.223	3.356	0.194	3.129	0.203		-0.05
etfdh	electron transferring flavoprotein, dehydrogenase	0.736	0.048	0.716	0.047	0.676	0.057		-0.063
Glud1	glutamate dehydrogenase 1	2.332	0.172	2.497	0.087	2.270	0.145		-0.063
got1	glutamic-oxaloacetic transaminase 1, soluble	0.561	0.049	0.619	0.004	0.646	0.039		0.09
got2	glutamatic-oxaloacetic transaminase 2, mitochon	2.196	0.183	2.496	0.117	2.328	0.150	0.104	
gpi	glucose phosphate isomerase 1	0.483	0.030	0.526	0.014	0.534	0.023		
Ndufs1	NADH dehydrogenase (ubiquinone) Fe-S protein	1.391	0.080	1.359	0.064	1.329	0.049		
Ndufv1	NADH dehydrogenase (ubiquinone) flavoprotein	0.773	0.058	0.781	0.036	0.763	0.036		

phb	prohibitin	0.544	0.035	0.586	0.053	0.551	0.014		
phb2	prohibitin 2	0.466	0.033	0.502	0.011	0.487	0.023		
Rhot1	Ras Homolog Family Member T1	0.206	0.022	0.194	0.009	0.188	0.009		-0.051
Rhot2	Ras Homolog Family Member T2	0.028	0.008	0.051	0.002	0.049	0.003	0.897	-0.046
Samm50	sorting and assembly machinery component 50 h	0.676	0.038	0.658	0.029	0.638	0.019	-0.024	-0.028
slc25a11	solute carrier family 25 (mitochondrial carrier oxo	0.683	0.107	0.953	0.027	0.932	0.054	0.391	
Slc25a4	solute carrier family 25 (mitochondrial carrier, ad	1.065	0.098	1.132	0.049	1.138	0.069	0.078	
Slc25a4/5/	solute carrier family 25 (mitochondrial carrier, ad	15.157	3.363	24.365	0.514	24.334	0.957	0.658	
Tufm	Tu translation elongation factor, mitochondrial	0.605	0.099	1.076	0.082	1.010	0.066	0.605	
Uqcrc1	ubiquinol-cytochrome c reductase core protein 1	3.766	0.198	3.714	0.153	3.685	0.139		
	**Fatty Acid Beta Oxidation and Biosynthesis**								
Acaa1a/b	acetyl-Coenzyme A acyltransferase 1A/1B	0.321	0.090	0.615	0.041	0.538	0.059	0.906	-0.152
Acaa2	acetyl-Coenzyme A acyltransferase 2 (mitochondr	1.453	0.314	2.739	0.170	2.693	0.171	0.785	
Acad11	acyl-Coenzyme A dehydrogenase family, member	0.586	0.080	0.462	0.031	0.465	0.058	-0.211	
Acadl	acyl-Coenzyme A dehydrogenase, long-chain	1.327	0.114	1.282	0.085	1.214	0.075		
Acadm	acyl-Coenzyme A dehydrogenase, medium chain	4.374	0.517	3.687	0.192	3.905	0.450	-0.13	
Acads	acyl-Coenzyme A dehydrogenase, short chain	0.828	0.075	0.812	0.075	0.732	0.074		-0.115
Acadvl	acyl-Coenzyme A dehydrogenase, very long chain	0.967	0.054	0.993	0.069	1.015	0.057		
Acot13	acyl-CoA thioesterase 13	1.890	0.218	1.807	0.102	1.769	0.144		
Acox1	acyl-Coenzyme A oxidase 1, palmitoyl	1.228	0.308	0.667	0.139	0.571	0.148	-0.519	-0.169
acsl1	acyl-CoA synthetase long-chain family member 1	1.419	0.113	1.469	0.227	1.625	0.168		0.125
Bdh1	3-hydroxybutyrate dehydrogenase, type 1	1.265	0.311	1.833	0.123	1.907	0.201	0.545	
Cpt1a	carnitine palmitoyltransferase 1a, liver	1.006	0.139	1.055	0.071	1.052	0.105		
Cpt1b	carnitine palmitoyltransferase 1b, muscle	0.199	0.018	0.175	0.021	0.197	0.026		
Cpt2	carnitine palmitoyltransferase 2	0.525	0.042	0.519	0.040	0.509	0.060		
crat	carnitine acetyltransferase	0.301	0.019	0.294	0.016	0.295	0.015		
Crot	carnitine O-octanoyltransferase	0.214	0.066	0.100	0.023	0.085	0.022	-0.634	-0.121
decr1	2,4-dienoyl CoA reductase 1, mitochondrial	2.576	0.217	2.268	0.164	2.156	0.162	-0.124	-0.051
Ech1	enoyl coenzyme A hydratase 1, peroxisomal	0.950	0.078	0.977	0.030	0.990	0.063		
echs1	enoyl Coenzyme A hydratase, short chain, 1, mito	0.535	0.123	0.808	0.042	0.777	0.048	0.601	
Eci1	enoyl-Coenzyme A delta isomerase 1	0.549	0.042	0.700	0.033	0.665	0.016	0.229	
Eci2	enoyl-Coenzyme A delta isomerase 2	0.095	0.017	0.111	0.009	0.099	0.007	0.174	
Ehhadh	enoyl-Coenzyme A, hydratase/3-hydroxyacyl Coe	6.645	1.272	3.968	0.160	4.369	0.563	-0.395	
Fabp3	fatty acid binding protein 3, muscle and heart	0.133	0.010	0.152	0.009	0.143	0.013	0.103	

Fabp4	fatty acid binding protein 4, adipocyte	3.936	0.989	3.452	0.174	3.296	0.445		-0.08
gpi	glucose phosphate isomerase 1	0.494	0.028	0.563	0.022	0.557	0.020		
Hadh	hydroxyacyl-Coenzyme A dehydrogenase(trifunct	4.660	0.430	4.465	0.356	3.842	0.359		-0.174
Hadha	hydroxyacyl-Coenzyme A dehydrogenase/3-ketoa	0.971	0.090	1.236	0.131	1.269	0.098	0.213	
Hadhb	hydroxyacyl-Coenzyme A dehydrogenase/3-ketoa	1.356	0.123	1.632	0.124	1.700	0.157	0.206	
HmgcL	3-hydroxy-3-methylglutaryl-Coenzyme A lyase	1.096	0.080	1.232	0.115	1.110	0.118	0.054	
Hmgcs1	3-hydroxy-3-methylglutaryl-Coenzyme A synthase	0.137	0.023	0.106	0.014	0.091	0.013	-0.228	-0.146
Hmgcs2	3-hydroxy-3-methylglutaryl-Coenzyme A synthase	0.167	0.039	0.268	0.072	0.254	0.039	0.454	0.06
Hsd17b4	hydroxysteroid (17-beta) dehydrogenase 4	1.502	0.190	1.193	0.052	1.154	0.093	-0.195	-0.044
	**Peroxisomal**								
Abcd3	ATP-binding cassette, sub-family D (ALD), membe	0.547	0.162	0.800	0.066	0.746	0.059	0.636	
cat	catalase	6.397	1.091	4.750	0.598	4.041	0.637	-0.254	-0.174
Ephx2	Epoxide Hydrolase 2	2.582	0.515	2.197	0.515	1.652	0.497	-0.192	-0.309
lonp1	lon peptidase 1, mitochondrial	0.181	0.053	0.354	0.024	0.359	0.028	1.05	
lonp2	lon peptidase 2, peroxisomal	0.029	0.007	0.029	0.002	0.030	0.003	0.149	
mdh1	malate dehydrogenase 1, NAD (soluble)	7.224	0.555	7.324	0.387	7.119	0.422		
Pecr	peroxisomal trans-2-enoyl-CoA reductase	2.900	0.619	2.286	0.455	1.926	0.459	-0.239	-0.193
prkaca	protein kinase, cAMP dependent, catalytic, alpha	0.080	0.002	0.081	0.003	0.082	0.004		
slc25a20	solute carrier family 25 (mitochondrial carnitine/a	0.665	0.042	0.673	0.041	0.634	0.021		-0.047
	**Antioxidants**								
akr1b1	aldo-keto reductase family 1, member B3 (aldose	3.241	0.907	1.705	0.501	3.947	1.535	-0.695	0.661
aldh2	aldehyde dehydrogenase 2, mitochondrial	2.730	0.107	3.670	0.240	3.384	0.270	0.246	
cat	Catalase	6.289	1.067	4.822	0.601	4.021	0.593	-0.226	-0.188
gpx1	glutathione peroxidase 1	0.959	0.100	1.063	0.100	2.052	0.114		0.725
gpx4	glutathione peroxidase 4	0.412	0.037	0.405	0.022	0.381	0.028		
gsr	glutathione reductase	0.352	0.026	0.383	0.029	0.372	0.034		
gsta3	glutathione S-transferase, alpha 3	1.233	0.071	1.563	0.076	1.582	0.101		
gstm1	glutathione S-transferase, mu 1	7.389	0.397	6.351	0.278	7.714	0.416		0.118
gstp1	glutathione S-transferase, pi 1	1.754	0.185	1.889	0.206	1.750	0.140		
msra	methionine sulfoxide reductase A	1.389	0.147	1.380	0.122	1.305	0.146		-0.069
prdx1	peroxiredoxin 1	3.785	0.586	4.631	0.359	4.239	0.329		
prdx2	peroxiredoxin 2	0.648	0.043	0.669	0.028	0.627	0.006		

prdx3	peroxiredoxin 3	1.194	0.116	1.227	0.070	1.157	0.088		
prdx5	peroxiredoxin 5	9.091	0.909	8.365	0.869	7.715	0.679		-0.115
prdx6	peroxiredoxin 6	0.914	0.052	0.839	0.034	0.921	0.044		
sod1	superoxide dismutase 1, soluble	2.243	0.206	1.898	0.163	1.818	0.138		
sod2	superoxide dismutase 2, mitochondrial	0.883	0.117	0.940	0.064	0.904	0.082		
txn1	thioredoxin 1	1.641	0.216	1.917	0.148	1.788	0.189		
txnrd1	thioredoxin reductase 1	0.244	0.023	0.250	0.012	0.251	0.010		
	**Proteostasis**								
Clpp	caseinolytic mitochondrial matrix peptidase prote	0.291	0.029	0.384	0.026	0.351	0.028	0.291	-0.09
Clpx	caseinolytic mitochondrial matrix peptidase chap	0.031	0.013	0.129	0.012	0.106	0.014	1.918	-0.217
Cryab	crystallin, alpha B	0.067	0.018	0.053	0.021	0.115	0.047	-0.431	0.607
hsp90b1	heat shock protein 90, beta (Grp94), member 1	0.735	0.143	1.194	0.065	1.089	0.035	0.506	
hspa1a	heat shock protein 1A	2.709	0.290	2.618	0.127	2.785	0.129		
hspa5	heat shock protein 5	0.605	0.057	0.608	0.031	0.625	0.029		
hspa9	heat shock protein 9	1.721	0.205	1.788	0.123	1.728	0.130		
hspd1	heat shock protein 1 (chaperonin)	1.096	0.423	4.295	0.487	3.540	0.447	1.866	-0.194
lonp1	lon peptidase 1, mitochondrial	0.181	0.053	0.354	0.024	0.359	0.028	1.05	
lonp2	lon peptidase 2, peroxisomal	0.029	0.007	0.029	0.002	0.030	0.003	0.149	

ƚ **The effects of significantly changed protein (in log scale) are highlighted in red for Aging and blue for Transgenic effect**; missing values are non-significant
