# Supplementary material for: Glutathione peroxidase‐1 overexpression reduces oxidative stress, and improves pathology and proteome remodeling in the kidneys of old mice
Source: Aging Cell. 2020 May 13;19(6):e13154. doi: 10.1111/acel.13154 (PMC7294784; doi:10.1111/acel.13154)
Supplement: Supplementary file 3 — Methods S1 [file ACEL-19-e13154-s003.docx]

**Supplementary Methods:**

**Mouse studies**

All animal experiments were approved by the University of Iowa Animal Care and Use

Committee. Mice overexpressing Gpx1 (Gpx1 Tg) were originally obtained from Dr. YS Ho[^1^](#_ENREF_1) and have been maintained at our institution for more than 20 years. These mice have been bred for more than 20 generations with C57BL/6J mice before study, so that they were considered C57BL/6J strain. All mice were housed in an Association for Assessment and Accreditation of Laboratory Animal Care–accredited facility. Both male and female mice were included in the study. Young mice (4-5 months old) and old mice (21-23 months old) were used. The age groups were chosen in accordance with the accepted principles for experiments on the biology of aging in mice.[^2^](#_ENREF_2)

**Immunohistochemistry**

Kidneys sections were first treated in citrate buffer for antigen retrieval, and then blocked for endogenous peroxidase (3% hydrogen peroxide for 8 minutes) and biotin (Vector Labs, Burlingame, CA). Sections were blocked by 10% normal goat serum in DAKO diluent buffer for 1 hour prior to incubation with primary antibody 4ºC overnight. After washing, sections were incubated with HRP-conjugated secondary antibody (DAKO rabbit Envision HRP system) for 30 minutes at room temperature. After washing with DAKO 1x buffer (2x 5 min), diaminobenzidine (DAKO DAB Plus) staining was performed. After rinsing in DAKO 1x buffer, DAB Enhancer was added. After a rinse in dH2O, sections were counterstained with hematoxylin (Surgipath). Slides were mounted with coverslips and images were taken using a microscope. The primary antibodies and conditions used were goat anti-Gpx 1 (R&D AF-3798, 1:200) and rabbit polyclonal anti-nitrotyrosine (Millipore #06-284,1:1000)

**Immunofluorescence**

Frozen sections (5 μm) were briefly fixed in 4% paraformaldehyde for 10 minutes, washed and permeabilized with 0.1% Triton-x, washed then blocked with 1% BSA with 0.1% Tween-20 for 1 hour, then stained with primary antibodies: goat anti-Gpx 1 (R&D AF-3798, 1:200) and rabbit anti VDAC-1 (Abcam 15895, 1:100) at 4ºC overnight. After multiple washing, secondary antibody AlexaFluor 568 donkey anti goat (1:200, BD

Biosciences) and AlexaFluo 488 goat anti rabbit (1:200, BD Biosciences) were incubated for 90 minutes, then washed and stained with Hoesct 33342 for 5 minutes. The images were taken using Leica confocal SP8.

**Western Blot**

Kidney tissues were homogenized in a glass homogenizer, in 0.5 ml RIPA buffer with protease inhibitors (Roche). Homogenates were sonicated, and centrifuged at 10,000xg for 10 minutes. Supernatants were collected, and protein concentrations were determined using the BCA method (Pierce). Equal amounts of protein were loaded to a NuPAGE 4-12% Bis-Tris gel (Novex). Electrophoresis was performed at 4^0^C in a MiniGel tank (Invitrogen) at 120V for 2 hours. Proteins were blotted at 4^0^C onto a PVDF membrane (Bio-Rad) using the same tank and a blot module (Invitrogen), at 20V for 3-5 hours. Blots were stained with Ponceau S to confirm equal loading and were blocked in 5% non-fat milk in PBS-Tween 20 (0.05%) (PBS-T). Blots were then incubated with primary antibodies (1:500 in 5% milk-PBS-T) at 4^0^C for overnight (~12 hours). Blots were washed in PBS-T, and incubated with HRP-conjugated secondary antibody (1:10,000 in 5% milk-PBS-T) at room temperature for 1 hour. Blots were washed with PBS-T, exposed to West Femto chemilluminascent reagent, and imaged in a Bio-Rad Imager. Bands were quantified using Image J (NIH).

**Assessment of glomerulosclerosis, interstitial fibrosis and cortical thickness**

Kidney sections (2 μm) were stained with PAS, Jones and Masson trichrome stain. Using PAS stain, we assessed every glomerulus within a kidney section and ranked them on a scale describing the hallmarks of aging-specific glomerulosclerosis from 0 to 3: normal with no injury (score 0); mild mesangiosclerosis (score 1), defined by mesangial expansion in less than 50% with limited Bowman capsule thickening; moderate mesangiosclerosis (score 2) defined by mesangial expansion greater than 50%, with extensive loss of capillary loop structure and podocytes and more extensive Bowman capsule thickening; severe glomerulosclerosis (score 3), defined by complete or nearly complete (>75%) glomerulosclerosis with extensive adhesion to Bowman capsules, shrinkage of glomerular tufts with little or no visible/patent capillaries. The percentage of glomeruli in each score is presented separately for glomeruli in the outer cortex and juxtamedullary glomeruli. All glomeruli are scored.

Masson trichrome staining was used to assess the degree of interstitial fibrosis. Three representative images at low power from each kidney section were used for quantification of the percentage of blue area relative to total tissue area by ImageJ (fiji version; National Institutes of Health, Bethesda, MD).

Cortical thickness was measured at three different sites for each kidney section, as a perpendicular distance from renal capsule to medullary regions, which are marked by arrays of tubules with frequent intratubular pink PAS-positive proteinaceous casts.

**Measurement of oxidative damage and redox status**

The levels of F2-isoprostanes in kidneys were determined by a previously described method [^3^](#_ENREF_3) with minor modifications. Briefly, 100 mg of tissue was homogenized in 10 ml of ice-cold Folch solution (CHCl3: MeOH, 2:1) containing butylated hydroxytoluene (BHT). The mixture was incubated at room temperature for 30 min. 2 ml of 0.9% NaCl was added and mixed well. The homogenate was centrifuged at 3,000g for 5 min at 4 °C. The aqueous layer was discarded while the organic layer was secured and evaporated to dryness under N2 at 37 °C. F2-isoprostanes were extracted and quantified by gas chromatography-mass spectrometry using the internal standard [2H4]8-Iso-PGF2α, which was added to the samples at the beginning of extraction to correct yield of the extraction process. Esterified F2-isoprostanes were measured using gas chromatography–mass spectrometry. The level of F2-isoprostanes in the tissues was expressed as nanograms of 8-Iso-PGF2α, per gram of tissue.

GSH and GSSG were extracted from tissue homogenate by treatment with 5% metaphosphoric acid. Proteins were precipitated upon incubation on ice (20 min) and then pelleted by centrifugation (10 min at 16,000 *g*). The supernatant was filtered (0.45-μm syringe filters) before analysis of GSH and GSSG by HPLC and electrochemical detection (ESA HPLC system, ESA Coularray electrochemical detector 5600A set at 750 mV). GSH and GSSG were eluted through a C18 column (Phenomenex Luna C18(2), 100 A, 3 μm, 150×4.6 mm) at 0.5 ml/min using an isocratic mobile phase consisting of 25 mM NaH2PO4, 0.5 mM 1-octane sulfonic acid, 4% acetonitrile, pH 2.7. GSH and GSSG concentrations were calculated employing GSH and GSSG standard curves constructed from peak areas, as previously described[^4^](#_ENREF_4).

**Statistical Analysis of Glomerular Injury Score**

To assess the significance of the two factors, namely, aging effect (i.e. young WT vs old WT) and transgenic GPX-1 effect in old (i.e. OTG vs OWT), we apply a proportional odds linear regression (POLR) model for analyzing the data. The response variable is the Glomerular score which can take values from 0, 1, 2, and 3. The higher score of this ordinal variable indicates increasing severity, without the assumption that the difference between score 0 and 1 is equal to 1 and 2 or 2 and 3. The POLR model implies that the log odds of $GS>k$ given the two factors YO and TG is equal to ${\beta_{1}\times YO+\beta_{2}\times TG-\zeta}_{k}$ for $k=0,1,2,,$ where $\beta_{1},\beta_{2}, \zeta_{1},\zeta_{2},\zeta_{3}$ are parameters. The parameter $\beta_{1}$ is the difference in the log odds of $GS>k$ between young mice and old mice, i.e., it is the age effects, and this difference is common across different $k=0,1,2$; hence the name of the proportional odds linear regression model. Positive $\beta_{1}$ indicates that aging tend to be associated with increasing GS. Similarly, $\beta_{2}$ is the transgenic effect. The proportional odds linear regression model is a sub-model of a more general model known as cumulative link regression model, which are same except using different link functions. For the proportional odds linear regression, the link function is the logistic function.

**Shotgun Proteomic Analysis of Glomeruli:**

**Laser Capture Microdissection of Glomeruli**

Formalin-fixed and paraffin-embedded tissues were sectioned, 10-*μ*m thick, onto DIRECTOR laser microdissection slides (Expression Pathology), and stained with PAS. All glomeruli were microdissected by Leica LMD 7000 Laser Microdissection System and collected into 0.5-ml microcentrifuge tube caps containing normal saline, and the samples were stored in –80°C until further processing. The glomerular dimensions were recorded for all of the microdissected glomeruli.

**LCM Glomerular Proteomic Sample Preparation**

Twelve samples were processed for shot-gun proteomics analysis, four from each group (YWT, OWT, OTG), with at least 100 glomeruli processed as above. Glomerular proteins were reduced, alkylated, and digested using filter-aided sample preparation (FASP) based on our previous method with a minor modification[^5^](#_ENREF_5). Briefly, glomeruli were lysed and solubilized in 100 μL of lysis buffer containing 3.5% SDS, 100 mM Tris-HCl pH 7.6, protease and phosphatase inhibitor (Sigma, St. Louis, MO). The suspensions were heated at 98°C on the thermomixer for 90 minutes with 15 second vortex every 15 minutes. The glomerular lysate were sonicated in a water bath for one hour, and then treated with 50 mM dithiothreitol (Sigma, St. Louis, MO) at 60°C for 30 minutes to reduce disulfide bonds. Free sulfhydryls were alkylated using FASP in 150 mM iodoaceamide in 8M urea (Sigma, St. Louis, MO), 100 mM Tris-HCl pH 8.5 at room temperature for 30 minutes. Proteins were then digested with 100 μl 0.02 μg/μl sequencing grade porcine trypsin (Promega, Madison, WI) in 50 mM triethylammonium bicarbonate (TEAB). Digested peptides were cleaned by StageTip (Thermo, Rockford, IL) and dried on a SpeedVac for a LC/MS-MS run.

**Liquid Chromatography and Mass Spectrometry**

Each sample was resuspended in buffer A, and separated by reverse phase XSelect CSH C18 2.5 um resin (Waters, Corporation, Milford, MA) on an in-line 150 x 0.075 mm column using an UltiMate 3000 RSLC nano system (Thermo, Rockford, IL). Peptides were eluted using a 60 min gradient from 97:3 to 60:40 buffer A:B ratio [Buffer A = 0.1% formic acid, 0.5% acetonitrile; buffer B = 0.1% formic acid, 99.9% acetonitrile]. Eluted peptides were ionized by electrospray (2.15 kV), followed by MS/MS analysis using higher-energy collisional dissociation (HCD) on an Orbitrap Fusion Lumos mass spectrometer (Thermo, Rockford, IL) in the top-speed data-dependent mode. MS data were acquired using an FTMS analyzer in the profile mode at a resolution of 240,000 over a range of 375 to 1500 m/z. Following HCD activation, MS/MS data were acquired for the top 12 peaks from each MS scan using the ion trap analyzer in the centroid mode and normal mass range with precursor mass-dependent, normalized collision energy between 28.0 and 31.0. Proteins were identified by searching the UniProtKB database using the Andromeda search engine in MaxQuant (Max Planck Institute of Biochemistry, Martinsried, Germany; version 1.6.0.16) with a parent ion tolerance of 3 ppm, a fragment ion tolerance of 0.5 Da. The database was searched using a decoy database with the reverse sequences in order to calculate the false discovery rate, which was determined to be 1%. Protein identifications were accepted for protein inference and quantification if they could be established with an FDR less than 1%[^6^](#_ENREF_6). Other search parameters were as follows: trypsin digestion with up to 2 missed cleavages; fixed modification of carbamidomethyl of cysteine; variable modifications of oxidation on methionine and acetyl on N-terminus; selected match between runs with 0.7 min match time window and 10 min alignment time window; and selected label-free quantitation with intensity-based absolute quantification (iBAQ) with a minimum ratio of 1[^7^](#_ENREF_7).

**Statistical Analysis of Shotgun Proteomics Data**

The contaminant and reversed peptide were first removed from the raw Maxquant data, to establish a raw file that was normalized by median value ignore zeros of each sample. Proteins with only one data of each sample group were excluded unless replicate values were observed from other sample group. The missing iBAQ intensity values were replaced with a half of the minimum value among all samples of each protein to facilitate further analysis. The data were then log2-transformed for normality. One sample from each group was excluded due to technical error (low median intensity) during the FASP digestion. Differentially expressed proteins between groups were determined by applying t test with Q-value calculated to adjust for false discovery rate. A total of 77 significant proteins (p < 0.1) between group OWT and YWT whose ratio were used to plot a heatmap.

**Ingenuity Pathway Analysis**

In order to further understand the biological events in relation to the aging effect on the glomerulus, a total of 77 significantly different proteins (p < 0.1) with at least 10% changes between group OWT and YWT were analyzed by Ingenuity Pathway Analysis version 42012434 (Ingenuity ® Systems, www.ingenuity.com, Mountain View, CA). Right-tailed Fisher’s exact test was used to calculate the p value and p value < 0.05 was considered significant in the canonical pathway. A total of 7 of aging related canonical pathways were select with p value < 0.0125 and Z-score ≥ 1.34 or ≤ −1.63, which was considered significant activation or inhibition respectively. The protein involved in these pathways whose ratio between different groups was also plotted on a heat map. Proteins involved in glomerulus related diseases or dysfunctions were also selected to compare their ratio between groups.

**Targeted Proteomics Analysis of Renal Tubules / Medulla**

Eighteen kidneys (6 from each YWT, OWT and OTG) from renal medulla were homogenized in RIPA buffer containing protease inhibitor cocktail. Total proteins of 100 µg from each sample were used for targeted proteomics analysis. Total proteins were mixed with 200 µL 1% SDS, 20 µL of BSA internal standard, heated for 15 min, and then precipitated with 1 mL acetone. The dried protein pellet was reconstituted in 100 µL Laemmli sample buffer and 20 µL (20 µg) was used to run a short (1.5 cm) SDS-PAGE gel. The gels were fixed and stained. Each sample was cut from the gel as the entire lane and divided into smaller pieces. The gel pieces were washed to remove Coomassie blue, reduced, alkylated, and digested overnight with trypsin. The mixture of peptides was extracted from the gel, evaporated to dryness in a SpeedVac and reconstituted in 150 µL of 1% acetic acid for analysis.

The analyses were carried out on a TSQ Quantiva triple quadrupole mass spectrometry system. The HPLC was an Ultimate 3000 nanoflow system with a 10 cm x 75 µm i.d. C18 reversed phase capillary column. 5 µL aliquots were injected and peptides were eluted with a 60 min gradient of acetonitrile in 0.1% formic acid.

The mass spectrometer was operated in the selected reaction monitoring mode. For each protein, the method was developed to measure 2 ideal peptides. Assay for multiple proteins were bundled together in larger panels. Data were analyzed using the program Skyline to determine the integrated peak area of the appropriate chromatographic peaks. The response for each protein was calculated as the geometric mean of the peptide areas. These values were normalized to the response for the BSA standard. The samples were also analyzed on a Thermo QEx system in the LC-full scan MS mode. The total ion current in those analyses is an indication of the amount of material present in the sample for normalization.

Additional ‘universal detection’ runs, high resolution accurate mass (HRAM) were also performed using an orbitrap system (ThermoScientific QEx plus), as an additional type of data that could be re-interrogated when needed.

**Statistical Analysis of Targeted Proteomics Data**

To analyze the effect of aging and GPX-1 transgenic (Tg) on the abundance of proteins,

let $\mathcal{G}$ be a pathway with $G$ proteins and $g$ be an arbitrary protein in $\mathcal{G}$, with a model for the data set:

$$Y=\beta+\sum_{g\in\mathcal{G}} \{\beta_{g}I_{g}+\beta_{g:old}I_{g:old}+\beta_{g:TG}I_{g:TG}\}+\epsilon,$$

where $Y$ is the log-transformed amount of protein and $\beta$, $\beta_{g}$, $\beta_{g:old}$ and $\beta_{g:TG}$ are the overall mean amount, protein-specific effect relative to the overall mean, protein-specific age effect and protein-specific transgenic effect, respectively, and all on the logarithmic scale. For a given protein $g$ in a pathway $\mathcal{G}$, the indicator variables are set as

$I_{g}= \left\{ \begin{aligned} 1 observation from protein g \\ 0 otherwise \end{aligned} \right.$;

$I_{g:old}= \left\{ \begin{aligned} 1 observation from protein g and from an old mouse \\ 0 otherwise \end{aligned} \right.$;

$I_{g:TG}= \left\{ \begin{aligned} 1 observation from protein g and from a transgenic mouse \\ 0 otherwise \end{aligned} \right.$.

In this model, the coefficient vector $\boldsymbol{\beta}=(\beta,\beta_{1},\beta_{1:old},\beta_{1:TG},\ldots,\beta_{G},\beta_{G:old},\beta_{G:TG})^{T}$ can be naturally grouped by proteins. Specifically, for $g\in\mathcal{G}=\{1,2,\ldots,G\}$, let $\boldsymbol{\beta}_{g}=(\beta_{g},\beta_{g:old},\beta_{g:TG})^{T}$ be the group coefficient vector for protein $g$, then the overall coefficient vector $\boldsymbol{\beta}=(\beta,\boldsymbol{\beta}_{1}^{T},\ldots,\boldsymbol{\beta}_{G}^{T})^{T}$. Note that there are two levels of sparsity in this model. At the group level, if $\boldsymbol{\beta}_{g}=\mathbf{0}$ for a protein $g$, it means that the protein has no effect. At the individual level, for a significant protein $g$ so that $\boldsymbol{\beta}_{g}\neq\mathbf{0}$, it is possible that $\beta_{g:old}=0$ or $\beta_{g:TG}=0$, which means that though this protein is significant, there is no age or transgnic effect for this protein. Our objective is then to select significant proteins at the group level and significant protein/age/transgenic effect at the individual level. This is called bi-level selection.

Through extensive simulation studies, we choose to apply the composite MCP penalty proposed by Breheny & Huang (2009) to perform bi-level selection for this data set. Specifically, the composite MCP penalized linear regression finds the estimation of $\boldsymbol{\beta}$ by the following minimization problem:

$$\hat{\beta}=arg min_{\beta}\{\frac{1}{2n}||y-X{\beta||}_{2}^{2}+\sum_{g=1}^{G} p_{\lambda,\gamma_{O}}\left( \sum_{k=1}^{K_{g}} p_{\lambda,\gamma_{I}}\left( \left| \beta_{\mathrm{gk}} \right| \right) \right)\}$$

where

$$p_{\lambda,\gamma}\left( \theta\right)=\left\{ \begin{aligned} \lambda\theta-\frac{\theta^{2}}{2\gamma}\quad\quad if \theta\leq\gamma\lambda\\ \frac{1}{2}\gamma\lambda^{2}\quad\quad if \theta>\gamma\end{aligned} \right.$$

is the MCP penalty function. Here $n$ is the sample size, $\mathbf{y}$ is the response vector, $X$ is the design matrix, $K_{g}=3$ is the number of coefficients in group $g$, $\lambda$ is the tunning parameter controlling the magnitude of penalty and $\gamma$ is a user specified parameter controlling the shape of the penalty function. In our implementation, we used 10-fold cross validation to select the best value of $\lambda$ and fix $\gamma_{I}=3$ and $\gamma_{O}=K_{g}\gamma_{I}\lambda/2$ as suggested by Breheny & Huang (2009). The analysis is performed using the R package grpreg written by Patrick Breheny.

References:

1. Cheng WH, Ho YS, Ross DA, Han Y, Combs GF, Jr., Lei XG. Overexpression of cellular glutathione peroxidase does not affect expression of plasma glutathione peroxidase or phospholipid hydroperoxide glutathione peroxidase in mice offered diets adequate or deficient in selenium. *J Nutr* 1997;127:675-680

2. Miller RA, Nadon NL. Principles of animal use for gerontological research. *Journals of Gerontology. Series A, Biological Sciences and Medical Sciences*. 2000;55:B117-123

3. Roberts LJ, Morrow JD. Measurement of f(2)-isoprostanes as an index of oxidative stress in vivo. *Free radical biology & medicine*. 2000;28:505-513

4. McLain AL, Cormier PJ, Kinter M, Szweda LI. Glutathionylation of alpha-ketoglutarate dehydrogenase: The chemical nature and relative susceptibility of the cofactor lipoic acid to modification. *Free radical biology & medicine*. 2013;61:161-169

5. Wisniewski JR, Zougman A, Nagaraj N, Mann M. Universal sample preparation method for proteome analysis. *Nature methods*. 2009;6:359-362

6. Nesvizhskii AI, Keller A, Kolker E, Aebersold R. A statistical model for identifying proteins by tandem mass spectrometry. *Analytical chemistry*. 2003;75:4646-4658

7. Schwanhausser B, Busse D, Li N, Dittmar G, Schuchhardt J, Wolf J, Chen W, Selbach M. Global quantification of mammalian gene expression control. *Nature*. 2011;473:337-342

8. Breheny, P., & Huang, J. (2009). Penalized methods for bi-level variable selection. *Statistics and Its Interface*, *2*(3), 369–380.
